# Supplementary material for: Genome-wide prediction of DNase I hypersensitivity using gene expression
Source: Nat Commun. 2017 Oct 19;8:1038. doi: 10.1038/s41467-017-01188-x (PMC5715040; doi:10.1038/s41467-017-01188-x)
Supplement: Supplementary file 1 — Supplementary Information [file 41467_2017_1188_MOESM1_ESM.pdf]

Supplementary Methods

Factors affecting cross-cell-type prediction accuracy

In order to investigate what factors may influence cross-cell-type prediction accuracy (i.e., r_C), we first grouped DHSs into two categories: "noisy loci" and "non-noisy loci" based on the true DH values measured by DNase-seq in the test cell types. Note that DHSs analyzed by BIRD are identified from the training data such that each DHS is active in at least one training cell type. Therefore, all these DHSs carry signals in the training data. However, since test cell types are not used to select these loci, a subset of DHSs may be inactive in all test cell types. For this subset of DHSs, the DH measurements in the test cell types are primarily noise. For such loci, the cross-cell-type correlation between the predicted and true DH levels in the test data is expected to be low since the correlation between a random noise and an uncorrelated variable is expected to be zero. For this reason, we identified DHSs with DH values (at log2 scale; same below) smaller than 2 in all 17 test cell types and labeled them as "noisy loci". After excluding the noisy loci, the remaining DHSs were labeled as "non-noisy loci". For the noisy loci, the mean r_C is small as expected (**Fig. 3a**, mean = 0.16). Conceptually, the low r_C at a noisy locus does not necessarily imply that the prediction model for that locus is inaccurate. It simply reflects the fact that the data in test cell types are primarily noise and do not contain enough biological variation for evaluating the model's ability to predict variation of biological signals across cell types. It is possible that a locus is active in a subset of training cell types and has a very good prediction model. However, the good cross-cell-type prediction performance of a model can only be observed when the test data are not all noise. When the test data are all noise, whether the model is good or not in terms of predicting cross-cell-type biological variation is not observable. Moreover, in real applications, it is not useful to study the cross-cell-type variation or differential DH of a locus in a set of new cell types if DH levels at this locus in these new cell types are all noise. For these reasons, we separated "noisy loci" from "non-noisy loci" in our subsequent analysis and evaluation.

For the non-noisy loci, we examined how their r_C may be influenced by the following factors which were computed separately in the training data and the test data using the true DH levels measured by DNase-seq.

1) Signal range (max-min spread): for each locus, the difference between its maximum and minimum DH values in all test cell types was calculated to characterize its signal range in the test data. Similarly, for each locus we also calculated the signal range in the training cell types. Signal range characterizes the magnitude of DH signal changes among different cell types.
2) Signal variability (coefficient of variation, i.e., CV): for each locus, we calculated the CV of its DH values in test and in training cell types separately. CV is the ratio of the standard deviation to the mean of the DH values. It characterizes the variability of DH across different cell types relative to the average DH level at a locus. As CV decreases, the cross-

cell-type DH signals will behave more and more like a constant. When CV is zero, the DH signal will become a constant across cell type.

3) Mean chromatin accessibility (mean DH): for each locus, the mean DH level in all test cell types was calculated to characterize its average chromatin accessibility. Similarly, we also calculated the mean DH level for each locus in training cell types.
4) Cell-type-specificity (no. of active or inactive cell types): for each locus, the number of test cell types in which the locus is active (i.e., DH level on log2 scale was larger than 2) was counted. The loci were then grouped into five categories: active in ≤ 1 cell type, active in 2 cell types, inactive in ≤ 1 cell type, inactive in 2 cell types, and others (i.e. active in ≥ 3 cell types and inactive in ≥ 3 cell types). Similarly, loci were also categorized based on their cell-type-specificity in the training data.

Supplementary Fig. 9a-f examines the relationship between the r_C and max-min spread, CV, and mean DH. Each dot in the plot represents a non-noisy locus. The red curves are the loess fit of the data. The Pearson's correlation coefficients are shown on top of the plots. These data show that max-min spread and CV had clear influences on r_C. Low max-min spread and low CV were linked to reduced r_C. The mean DH, on the other hand, did not show strong correlation with r_C.

Supplementary Fig. 9g-h examines the relationship between the r_C and cell type specificity. The distribution of r_C for loci in each cell-type-specificity category is shown. This analysis shows that highly cell-type-specific loci (active in ≤ 1 cell type or inactive in ≤ 1 cell type) tend to have smaller r_C.

Based on the above findings, next we concentrated on analyzing max-min spread, CV and cell-type-specificity. From all non-noisy loci, we identified those with small max-min spread (defined as max-min spread statistic below its 1st quartile) and labeled them as loci with "low max-min spread". The remaining loci were labeled using "high max-min spread". This was done for test data and training data separately. Similarly, we identified non-noisy loci with "low CV" (defined as CV below its 1st quartile) and "high CV" (all remaining non-noisy loci). For cell type specificity, non-noisy loci active in ≤ 1 cell type or inactive in ≤ 1 cell type were labeled as loci with "high cell-type-specificity", and the remaining loci were labeled using "low cell-type-specificity". **Fig. 3a** shows the distribution of r_C for loci in each category. The figure shows that loci with low max-min spread, low CV and high cell-type-specificity on average had lower r_C compared to those with high max-min spread, high CV and low cell-type-specificity.

The Venn diagrams in **Fig. 3b-d** show the percentage of loci with low cross-cell-type prediction accuracy (defined as the 177,816 DHSs with r_C<0.25) that were found to be noisy loci in test data, or loci with low max-min spread, low CV or high cell-type-specificity in test or training data. **Fig. 3b** shows that the majority (85%) of loci with low r_C can be explained by these factors.

In real applications, true DH values in the test cell types are not available. Thus, we repeated the above analyses by using the predicted DH values to replace true DH values in the test cell types.

Noisy loci, max-min spread, CV and cell type specificity were all recalculated using the predicted DH values. Similar results were obtained (**Supplementary Fig. 10**).

Factors correlated with r_C may be used to screen for loci with high cross-cell-type prediction accuracy. For example, in **Fig. 3e** we categorized loci into two groups: (1) "filtered loci", which are noisy loci in test cell types, and loci with low max-min spread, low CV or high cell-type-specificity in either test or training cell types, and (2) "retained loci", which are all the other loci. The loci were categorized based on their true DH values in training cell types and predicted DH values in test cell types. This figure shows the r_C distribution for each category as well as the r_C distribution for all loci. If one chooses to focus on "retained loci", the mean r_C would be 0.6 (compared to the mean of 0.5 for all loci, and 0.43 for loci that are filtered out), and 74% and 30% of loci would have $r_C > 0.5$ and >0.75 respectively.

We note that the definition of noisy loci and test data factors (i.e., loci with low max-min spread, low CV or high cell-type-specificity in test data) are dependent on the test cell types. In real applications, they will be defined based on the cell types on which BIRD is applied to make predictions. For simplicity, these cell types are also called test cell types below. Different applications may involve different test cell types. Thus, the set of loci labeled as noisy loci, low max-min spread, low CV or high cell-type-specificity in test cell types varies from one application to another. As a result, the loci removed from the analysis by the above filters will be different in different studies. For example, consider a locus that is active in a subset of training cell types and therefore has a good prediction model. The locus may be filtered out as a "noisy locus" in some applications where the data in test cell types are all noise and predicting cross-cell-type variation at the locus is not practically useful, and it may be retained as "non-noisy locus" in other applications where the data in test cell types contain some real biological variation and there is a need for studying differential DH at the locus.

Comparing BIRD with ChromImpute

Among the cell types analyzed in this study, DH profile in 10 cell types (GM12878, HMEC, HSMM, HSMMtube, HUVEC, Monocytes, NH-A, NHDF-Ad, NHEK, NHLF) have also been predicted previously by ChromImpute[1]. We compared BIRD and ChromImpute predictions in these 10 common cell types. Unlike BIRD predictions which were based on gene expression data alone, ChromImpute predictions were based on multiple functional genomic data types, particularly ChIP-seq data for multiple histone modifications including H3K4me1, H3K4me3, H3K36me3, H3K27me3, H3K9me3, H3K27ac and H3K9ac, which were selected by ChromImpute as the most informative predictors. For ChromImpute, both the predicted (i.e., imputed) DH signal and the true DH signal (i.e., $-\log_{10}$ p-value of DNase-seq counts relative to expected background calculated by MACS[2]) were downloaded from the Roadmap Epigenomics compendium[3] and converted to 200 bp resolution using the "Convert" program provided by ChromImpute. For BIRD, a leave-one-out approach was applied to make predictions. In other words, BIRD was trained by 56 cell types

(test cell type excluded) with both DNase-seq and exon array data and then applied to predict DH for the test cell type. The mean DH profile of the 56 training cell types was also obtained as a control (indicated by "Mean"). We compared DH signals predicted by different methods at the 1,108,603 DHSs obtained from all 57 ENCODE cell types. Of note, the ChromImpute-reported signals were based on $-\log_{10}$ *p*-value ("pval-signal") and the BIRD-reported signals were based on log-transformed normalized read counts ("read-signal"). In order to conduct a fair comparison, we performed four types of analyses. First, we used the true pval-signal calculated by MACS as the gold standard for evaluating ChromImpute predictions, and we used the true read-signal from DNase-seq data as the gold standard for evaluating BIRD and Mean predictions. We calculated the cross-locus Pearson's correlation between the predicted and true pval-signal for ChromImpute, and the cross-locus Pearson's correlation between the predicted and true read-signal for BIRD and Mean, and compared them in **Fig. 3h**. Second, we replaced the Pearson's correlation by Spearman's rank correlation and repeated the above analysis again (**Supplementary Fig. 15a**). Third, we used the true pval-signal calculated by MACS as the gold standard (which should favor ChromImpute because ChromImpute is trained by the pval-signal) to evaluate different methods. Here since the gold standard and the signals from different methods could be at different scales and did not necessary follow a linear relationship, we only calculated the cross-locus Spearman's rank correlation between the gold standard and predicted signals. We then compared different methods in **Supplementary Fig. 15b**. Fourth, we used the true read-signal as the gold standard (which should favor BIRD) and calculated the cross-locus Spearman's rank correlation between the gold standard and predicted signals. Different methods were compared using this gold standard in **Supplementary Fig. 15c**. Similarly, we have also calculated the cross-cell-type correlation r_C using these four types of analyses. The results were shown in **Fig. 3i** and **Supplementary Fig. 15d-f**. In all four analyses, BIRD and ChromImpute had comparable prediction performance. Note that BIRD prediction was only based on gene expression data and the model was trained using 56 cell types, whereas ChromImpute prediction was based on multiple data types and the model was trained using more cell types (i.e., data from 126 epigenomes, each epigenome with multiple data types). In the comparisons of cross-locus correlation (**Fig. 3h**, **Supplementary Fig. 15a-c**), there were three cases (i.e., Monocyte, GM12878, and NHEK) for which ChromImpute performed well above BIRD. For these three cases, the ChromImpute training data (after excluding these three test cases) contained highly similar cell types "Primary monocytes from peripheral blood", "Primary B cells from peripheral blood", and "Foreskin Keratinocyte Primary Cells" (similar to Monocyte, GM12878, and NHEK respectively). By contrast, the 57 cell types used in the BIRD analysis only contained one Monocyte cell line (**Supplementary Data 1**). When it was used as the test cell type in the leave-one-out cross-validation, no other monocyte samples were contained in the training data. Similarly, GM12878 was the only lymphoblastoid cell line, and NHEK was the only epidermal keratinocytes cell line in our 57 cell types. Thus, BIRD predictions for these three cases were based on models trained using much less similar cell types.

Next, we compared the ability of BIRD and ChromImpute to predict differential DH between two sample types using the 10 test cell types (45 pairs in total). For each pair of the cell types, both BIRD and ChromImpute were used to predict differential DH, and the correlation between the predicted and true DH differences was computed and compared. The analysis was applied to all loci and differential loci (i.e., DHSs with |true log2-scale DH difference between the compared cell types|>1, after filtering out loci with log2 DH level smaller than 2 in both cell types) respectively. Cell type pairs were divided into two groups based on the median value of the similarity (i.e., Pearson's correlation of the true DH profiles) between two compared cell types. BIRD and ChromImpute were compared in each group. Similar to the previous analyses, four different types of analyses were used to evaluate the predictions. The results were shown in **Fig. 3j** and **Supplementary Fig. 15g-i**. **Fig. 3j** shows Pearson's correlation between the predicted and true differential signals of the same type (i.e., read-signal vs. read-signal, or pval-signal vs. pval-signal) across all loci and differential loci. **Supplementary Fig. 15g** shows Spearman's rank correlation between the predicted and true differential signals of the same type. **Supplementary Fig. 15h-i** shows Spearman's rank correlation between the predicted differential signals and the true differential signals defined either by read-signal or pval-signal (Spearman's correlation was used here since the comparison involves computing correlation between different signal types).

We note that BIRD prediction was only based on gene expression data while ChromImpute prediction used ChIP-seq data of multiple histone modifications which more directly measure the regulome and has been shown to largely overlap with DNase I hypersensitive sites[4, 5]. As gene expression data are far more widely available compared to histone modification ChIP-seq data, BIRD can have a much broader range of applications.

Predicting transcription factor binding sites

We first applied BIRD to predict TFBSs for 9 TFs (ELF1, GABPA, MAZ, NFYB, NRF1, SP1, TCF3, USF1, and YY1) in GM12878. BIRD was trained using the same 40 training cell types used in **Figs 2** and **3**. The training data did not contain GM12878. The trained models were used to predict DH at the 912,886 DHSs obtained from the 40 training cell types. The predictions were based on exon array data generated by three different laboratories including University of Washington (UW; GEO accession number: GSM472901, GSM472902, GSM472931), Duke University (Duke; GEO accession number: GSM993481, GSM993482, GSM993483), and University of Chicago (Chicago; GEO accession number: GSM245656). These exon array samples were normalized together with the training exon array samples as described above. For each TF, DNA motif obtained from TRANSFAC[6] or JASPAR[7] (**Supplementary Data 3**) was computationally mapped to the human genome using CisGenome[8] (using default likelihood ratio ≥ 500 cutoff). DHSs (i.e., 912,886 genomic bins) that overlapped with motif sites were retained for subsequent analyses. These motif-containing DHSs were ranked in decreasing order based on the predicted DH level. As a comparison, motif-containing DHSs were also ranked using three other methods: the true DH

level at each DHS from the corresponding DNase-seq data ("True"), the DH level predicted based on the mean DH profile of the 40 training cell types ("Mean"), and the highest motif mapping score of each DHS (i.e., the maximal CisGenome-reported log likelihood ratio score of all motif sites mapped in a DHS) ("Motif").

To evaluate the prediction performance of different methods, reproducible peaks from ChIP-seq data for each test TF in the test cell type were downloaded from ENCODE (http://hgdownload.cse.ucsc.edu/goldenPath/hg19/encodeDCC/wgEncodeAwgTfbsUniform/). These reproducible peaks were defined by ENCODE using irreproducible discovery rate[9] (IDR) <0.02. Reproducible ChIP-seq peaks overlapped with motif sites were used as the gold standard. The prediction performance of different methods was compared by three types of plots. First, the sensitivity was plotted as a function of the false discovery rate (FDR) (i.e., sensitivity-FDR curve, **Fig. 4a**, **Supplementary Fig. 18**). Sensitivity was computed as the percentage of the gold standard peaks that were recovered by the predicted TFBSs. FDR was calculated as the percentage of predicted TFBSs that were not overlapped with the gold standard. Second, we plotted the true positive rate versus false positive rate, also known as the receiver operating characteristic (ROC) curve (**Fig. 4b**, **Supplementary Fig. 19**). For ROC, true positive rate was computed as [number of predicted TFBSs that were overlapped with the gold standard]/[total number of motif-containing DHSs that were overlapped with the gold standard]. False positive rate was computed as [number of predicted TFBSs that were not overlapped with the gold standard]/[total number of motif-containing DHSs that were not overlapped with the gold standard]. Third, the number of predicted TFBSs was plotted as a function of FDR (i.e., FDR curve, **Fig. 4c**, **Supplementary Fig. 20**). To generate curves in the plots, each statistic was evaluated at the top *N* ranked predicted sites (*N* = 50, 100, 150, …, 950, 1000, 2000, 3000, …, up to the maximal number of predicted sites) and then plotted to obtain the performance curves. We have also calculated the area under the sensitivity-FDR curve (AUSFC) and the area under the ROC curve (AUROC) and compared different methods accordingly (**Fig. 4d-e**).

Next, we performed similar analyses on 3 TFs (ELF1, GABPA and MAZ) in K562 (**Supplementary Figs 25-26**). Since K562 was not in the 57 cell types initially used in this study, the prediction models used for K562 were trained by all 57 cell types for 1,108,603 DHSs. Predictions were made using exon array data generated by three different laboratories: UW (GEO accession number: GSM472910, GSM472926, GSM472927), Duke (GEO accession number: GSM993551, GSM993553, GSM993555), and Kaohsiung Medical University (KMU; GEO accession number: GSM613529, GSM613530, GSM613531, GSM613532). For predictions based on "Mean", the mean DH profile was based on 57 cell types.

Comparing BIRD with PIQ and CENTIPEDE for predicting TFBSs

To benchmark the TFBS prediction results from BIRD, we compared BIRD with two state-of-the-art methods PIQ[10] and CENTIPEDE[11]. Both PIQ and CENTIPEDE predict TFBSs based on the true

DNase-seq data and TF motifs. To perform a fair comparison, for each test TF, we used PIQ (default setting) and CisGenome to map motif sites and obtained motif sites that can be reproducibly mapped by both algorithms. BIRD, PIQ and CENTIPEDE were then evaluated based on this common set of motif sites. BIRD makes predictions for each motif-containing DHS. PIQ was run using its default settings to generate a prediction for each motif site. CENTIPEDE was run based on the DNase-seq signals extracted from a flanking region of 100 bp from each side of a motif site. For PIQ and CENTIPEDE, after predictions were made for each motif site, we scored each DHS using the maximal prediction score from all motif sites contained in the DHS. DHSs were then ranked based on the scores produced by each method. Prediction performances of different methods were then compared using TF ChIP-seq data following a similar protocol as before. The comparison results were shown in **Supplementary Figs 21-24 and 27-28**.

Predicting MYC binding sites in P493-6 B cell lymphoma

To further demonstrate TFBS prediction by BIRD in a realistic setting, we also applied BIRD to a non-ENCODE cell line, P493-6 B cell lymphoma, to predict MYC binding sites. This dataset did not have corresponding DNase-seq data. Thus, PIQ, CENTIPEDE and TFBS prediction based on true DH were not applicable. We compared BIRD with the mean DH and motif only methods.

We trained BIRD using all 57 cell types for 1,108,603 DHSs and then applied it to predict the DH profile of the P493-6 B cell lymphoma using exon arrays in a GEO dataset generated by a non-ENCODE lab[12] (GEO accession number: GSM798329, GSM798330, GSM798331). Using the predicted DH, we predicted MYC binding sites. MYC motif (TRANSFAC accession number: M00799) was computationally mapped to the genome by CisGenome (using default likelihood ratio $\geqslant 500$ cutoff). MYC binding sites were then predicted by ranking motif-containing DHSs using BIRD-predicted DH. The predictions were evaluated using MYC ChIP-seq data[13] in P493-6 cells. MYC ChIP-seq data were obtained from GEO (GEO accession number: GSM1234501, GSM1386347). Reads were aligned to human genome (hg19) using bowtie[14] and the uniquely mapped reads were retained for peak calling. This dataset does not have replicate samples and therefore one cannot use the IDR procedure to define peaks. ChIP-seq peaks were identified using CisGenome two-sample (IP vs. control) peak calling at the FDR cutoff of 0.01. ChIP-seq peaks that contained MYC motif sites were used as the gold standard.

Supplementary Fig. 29 compares the prediction performance of different methods in terms of sensitivity-FDR curve, ROC curve, and the number of predicted TFBSs at different FDR levels. At the 10%, 25%, and 50% FDR level, BIRD predictions gave a sensitivity of 0.37, 0.68, and 0.91 respectively, as compared to 0, 0, and 0 by the motif only approach, and 0.08, 0.20, and 0.55 by the mean DH approach (**Supplementary Fig. 29a**). We note that accurate prediction of MYC binding sites can be complicated by the fact that the motif bound by MYC, known as E-box motif, is also recognized by many other TFs (e.g., USF1). Thus, motif sites with high predicted DH levels can be bona fide binding sites of other TFs but labeled as false positives by MYC ChIP-seq data.

For example, at 80% sensitivity level, the FDR of BIRD was 34%. The 34% predictions classified as false positives may be real binding sites of other E-box binding TFs. Considering this complication, BIRD performed reasonably well. At the 10%, 25%, and 50% FDR level, BIRD generated 5000, 12,000, and 24,000 predicted MYC binding sites, which were substantially more than the number of the predicted sites by the mean DH (1000, 3000 and 14,000) and motif only (0, 0 and 0) methods (**Supplementary Fig. 29c**). The areas under the sensitivity-FDR curve and ROC curve (AUSFC and AUROC) for BIRD (AUSFC=0.59, AUROC=0.91) were also substantially better than the mean DH (AUSFC=0.35, AUROC=0.78) or the motif only (AUSFC=0.04, AUROC=0.61) approach. As two examples, **Supplementary Fig. 29d** show predictions in two genomic regions. In both regions, the DH levels predicted by BIRD at MYC motif sites were consistent with MYC binding activities measured by MYC ChIP-seq. As a comparison, the mean DH approach did not perform well for predicting MYC ChIP-seq. For the motif only approach, many motif sites identified by this approach were not bound by MYC (e.g., the first two motif sites in the second genomic region). Using the motif information alone, it was difficult to distinguish these sites from motif sites bound by MYC. However, by incorporating BIRD-predicted DH information, the difference between the bound and unbound motif sites became clear.

Analysis of three known MYC targets using PDDB

To generate **Fig. 6a-c**, we searched PDDB by entering keywords “b cell lymphoma” and “embryonic stem cell” in the “Cell Type” searching field in the user interface. Among the query results, GSM798329 is a P493-6 B cell lymphoma sample and GSM993504 is a H9 human embryonic stem cell sample (the other two H9 samples GSM993505 and GSM993506 showed similar results, **Supplementary Fig. 31**). Predicted DH profiles at the promoters of three genes *FBL*, *LIN28A* and *BLMH* were retrieved and visualized for these samples.

Analysis of SOX2 binding sites using PDDB

SOX2 binding sites in H9 human embryonic stem cells were obtained by analyzing a published ChIP-seq dataset (GEO accession number: GSM1139040, GSM1139041). CisGenome[8] two-sample analysis was used to generate the binding peak list at the FDR cutoff of 0.01. DHSs overlapping (n=6931) with the peak regions were retained as SOX2 binding sites for subsequent analyses (**Supplementary Data 4**).

To generate **Fig. 6d-e**, predicted DH values for 6931 SOX2 binding sites and 10,000 randomly selected DHSs (“random DHSs”) were retrieved from PDDB for all 2000 exon array samples. For each sample, we computed $\delta = \bar{y}_{\mathrm{B}} - \bar{y}_{\mathrm{R}}$ to characterize the overall DH enrichment level at the SOX2 binding sites. Here $\bar{y}_{\mathrm{B}}$ is the average predicted DH value of the 6931 SOX2 binding sites, and $\bar{y}_{\mathrm{R}}$ is the average predicted DH value of the 10,000 random DHSs. Samples were rank-ordered based on δ. The heatmap in **Fig. 6d** shows how the predicted DH varied across the sorted

samples. In the heatmap, each row is a SOX2 binding site, and each column is a sample. Rows were sorted based on the difference between the average DH from the 10% highest ranked samples and the average DH from the 10% lowest ranked samples. To display the cross-sample variation clearly, colors in the heatmap reflect the standardized DH values (i.e., values within each row were standardized to have zero mean and unit standard deviation (SD) before color coding) to minimize the locus effects. **Fig. 6e** shows the overall DH enrichment level δ for each sample in **Fig. 6d**.

To generate **Fig. 6f-g**, predicted DH values at the 6931 SOX2 binding sites (obtained from undifferentiated stem cells) were retrieved from PDDB for differentiating H7 stem cells collected at day 2, 5 and 9 after the initiation of differentiation. At each differentiating day, there were two replicate samples (day 2: GSM1033352, GSM1033353; day 5: GSM1033346, GSM1033347; day 9: GSM1033348, GSM1033349) whose predicted DH profiles were averaged and then displayed in **Fig. 6f**. As controls, the true DNase-seq data for undifferentiated H7 stem cells ("0 day") and H7 cells differentiated for 14 days (both the 0 day and 14 day samples were included in the 57 ENCODE cell types used by BIRD to train the prediction model) from ENCODE were also displayed in the same heatmap. Rows in the heatmap are SOX2 binding sites. They were sorted based on the difference between the DH in day 0 and DH in day 14. DH values within each row were standardized to have zero mean and unit SD for color coding. **Fig. 6g** shows the unstandardized DH values for both the 6931 SOX2 binding sites and 10,000 random DHSs. The values were predicted for day 2, 5 and 9 and were based on true DNase-seq for day 0 and 14. The figure shows that the overall DH level at SOX2 binding sites decreased as stem cell differentiation progressed. By contrast, such a decreasing pattern was not observed at random DHSs.

Analysis of MEF2A binding sites using PDDB

MEF2A ChIP-seq uniform peaks in GM12878 were downloaded from ENCODE (http://hgdownload.cse.ucsc.edu/goldenPath/hg19/encodeDCC/wgEncodeAwgTfbsUniform/). Among the 2000 PDDB samples, we retrieved 1061 samples whose *MEF2A* gene expression level was above the average *MEF2A* expression level across all PDDB samples (**Supplementary Fig. 32a**). These 1061 *MEF2A*-expressing samples were used to group MEF2A binding sites obtained from GM12878 into different subclasses.

Before the analysis, the MEF2A binding motif (JASPAR motif accession number: MA0052.2) was mapped to the human genome using CisGenome[8] (likelihood ratio ≥ 500). MEF2A ChIP-seq peaks that contained the MEF2A motif were retained, and DHSs that overlapped with these peaks were retrieved. The retrieved DHSs were further filtered to exclude noisy loci (loci with predicted DH level smaller than 2 in all samples). We then retrieved loci with high variability (coefficient of variation of the predicted DH level across the 1061 PDDB samples > 0.4). After the preprocessing, 2011 DHSs were obtained for subsequent analyses (**Supplementary Data 5**).

Using the predicted DH for the 1061 *MEF2A*-expressing PDDB samples, we identified subclasses of the 2011 DHSs by grouping them into 9 clusters using k-means clustering. Before clustering, predicted DH levels for each locus were standardized across the 1061 samples to have zero mean and unit SD. The cluster number (*K*=9) was determined as follows. First, we grouped the DHSs into *K* (*K* = 1, 2, …, 40) clusters. Then, for each clustering result, we calculated the total within-cluster sum of squared error $SSE(K) = \sum_l \sum_m \left(\hat{y}_{lm} - \bar{y}_{c_l m}\right)^2$ where $\hat{y}_{lm}$ is the standardized predicted DH for locus l and sample m, c_l is the cluster that contains locus l, and $\bar{y}_{c_l m}$ is the mean predicted DH of all loci in cluster c_l. Next, we calculated the forward difference of the 40 *SSE*s (i.e., *SSE*(*K+1*)- *SSE*(*K*)) and scaled it by the *SSE* from *K*=1. Finally, the first *K* with scaled *SSE* difference less than 0.01 was selected as the final cluster number (**Supplementary Fig. 32b**). To shed light on biological functions of each cluster, each DHS was annotated with its closest RefSeq gene using CisGenome. We then performed functional annotation analysis on each cluster using the Database for Annotation, Visualization and Integrated Discovery[15, 16] (DAVID), where the background was set to be all RefSeq genes. From DAVID results, we obtained enriched functional terms with FDR ≤0.1 and Fold Enrichment ≥ 2. These enriched functions are listed in **Supplementary Data 6**.

In addition to clustering DHSs, we also grouped the 1061 samples into 9 clusters. The cluster number was determined in a similar fashion as above (**Supplementary Fig. 32c**). Samples in each cluster are listed in **Supplementary Data 7**.

Fig. 6h-i show the row-standardized DH level for each locus (row) in each sample (column). A few representative cell types in each sample cluster are shown on the top. For each DHS cluster, a few enriched functions identified by DAVID are shown on the right. These figures demonstrate how PDDB can be used to further classify binding sites obtained from a ChIP-seq experiment (i.e., MEF2A binding sites in GM12878 in this example) into functional subgroups. MEF2A is known to play functional roles in neural differentiation[17, 18], B cell developmemt[19] and muscle development[20]. Suppose such knowledge was not available, the analyses in **Fig. 6h-i** can be used to provide clues on these functional connections. For instance, DHSs-cluster-5 showed higher DH level in neuron and brain related samples (sample-cluster-9) (**Fig. 6i**). Consistent with this, the DAVID analysis shows that “regulation of neuron projection development”, “regulation of neuron differentiation”, “axonogenesis”, and “regulation of neurogenesis” genes were enriched in DHSs-cluster-5. DHS-cluster-6 showed higher DH level in lymphoblastoid and B cell samples (sample-cluster-8) (**Fig. 6i**). Consistent with this, the DAVID analyses identified enrichment of “immune response” genes in DHS-cluster-6. DHS-cluster-7 showed higher DH level in muscle related samples (sample-cluster-7) (**Fig. 6i**). Muscle is a tissue type with high metabolism rate. Correspondingly, the DAVID analysis identified enrichment of functions related to “metabolic processes”, “cell motion” and “cell migration” in DHS-cluster-7. Of note, for each DHS cluster, PDDB has connected its activity to many biological contexts not covered by ENCODE. For instance, sample-cluster-7 contained coronary artery smooth muscle and cardiac precursor cell, sample-cluster-9 contained entorhinal cortex and motor neuron, etc. These sample types were not

available in ENCODE. This illustrates how PDDB can help one explore a wide variety of biological contexts whose regulome may not be available in other existing regulome databases.

Differentiation of iPSCs to dopaminergic neurons

The exon array data used for this analysis were generated from a differentiation system where the human iPSC cell line ND27760 was differentiated into dopaminergic neurons. The data are available from the GEO (accession number: GSE93012). The data were generated as follows. The iPSC line ND27760 (passages 25–30) maintained in Ying Lab was the same line described in our previous publication[21] which was originally derived from human skin fibroblasts by cell reprogramming. The iPSCs were maintained as feeder-free cultures in mTESR1 medium (StemCell Technologies, Vancouver, BC, Canada, http://www.stemcell.com) in 5% CO2/95% air conditions at 37°C and were passaged using dispase (Life Technologies). The iPSC line has been authenticated using short tandem repeat (STR) analysis. Karyotype analysis of G-banded metaphase chromosomes was performed to confirm the chromosomal integrity of these iPSCs. The iPSC line has been tested to confirm absence of mycoplasma contamination. Immunocytochemistry for pluripotent cell markers (*NANOG*, *OCT4*, *TRA-1-60*, and *SSEA-3*) and embryoid body differentiation have been performed to characterize the pluripotency of this iPSC line. These were described in our previous publication[21].

A previously reported dual-SMAD inhibition protocol[22] was used to differentiate human iPSCs into dopaminergic neurons. iPSCs were plated (35×10^3–40×10^3 cells per cm^2) and grown for 11 days on matrigel (BD) in knockout serum replacement medium (KSR) containing DMEM, 15% knockout serum replacement, 2 mM L-glutamine and 10 µM β-mercaptoethanol. KSR medium was gradually shifted to N2 medium starting from day 5 of differentiation. On day 11, media was changed to Neurobasal medium with B27 supplement and L-Glutamine (Invitrogen) supplemented with CHIR (until day 13) and with BDNF (brain-derived neurotrophic factor, 20 ng/ml, R&D), ascorbic acid (0.2 mM, Sigma), GDNF (glial cell line-derived neurotrophic factor, 20 ng/ml; R&D), TGFβ3 (transforming growth factor type β3, 1 ng/ml; R&D), dibutyryl cAMP (0.5 mM; Sigma), and DAPT (10 µM; Tocris) for 9 days. On day 20, cells were dissociated using Accutase (Sigma-Aldrich) and replated (300×10^3–400×10^3 cells per cm^2) on dishes coated with polyornithine (15 µg/ml) and laminin (1 µg/ml) in differentiation medium (Neurobasal medium plus B27 supplement, BDNF, ascorbic acid, GDNF, dibutyryl cAMP, TGFβ3 and DAPT) until the desired maturation stage for RNA preparation. Dopaminergic neurons were harvested after being replated and matured in vitro for 30 days. RNAs were extracted from iPSCs and dopaminergic neurons using RNeasy kit (Qiagen) and were subjected to Human Exon 1.0 ST Array (Affymetrix). The experiment was repeated twice, yielding two exon array samples for each condition (i.e., before or after differentiation).

ChIP-qPCR protocol

Primers were designed using Primer3[23] (http://biotools.umassmed.edu/bioapps/primer3_www.cgi). All primers have been subjected to *in silico* specificity screen using BLAST in human genome database. Primer sequence can be found in **Supplementary Data 8**.

iPSCs and iPSC-derived dopaminergic neurons obtained using the above protocol were subjected to ChIP for H3K4me1 by using the MAGnify ChIP system (Invitrogen) following the manufacturer's protocol. Chromatin was immunoprecipitated by using mouse anti-Histone H3K4me1 antibody (Active Motif, Catalog No: 39635) and Dynabeads magnetic beads (Invitrogen). Mouse IgG served as the control. ChIP-enriched DNA was subjected to quantitative PCR (qPCR) using CFX Connect Real-Time System (BIO-RAD). The specificity of PCR products is controlled by both PCR melting curves and PCR product sequencing. For each condition (i.e., before or after differentiation), the qPCR signal for a locus was obtained from three technical replicates and the fold enrichment was computed as $y = 2^{-(Ct_{avg}H3K4me1\ IP\ -Ct_{avg}IgG)}$ where Ct_{avg} represents mean Ct values from the three replicates. The differential qPCR signal between two conditions for a locus was characterized as the difference in y between the two conditions (i.e., y_{neuron} - y_{iPSC}).

Predicting differential DH during neuron differentiation

To predict DH of iPSCs and iPSC-derived dopaminergic neurons, gene expression from different replicates of the same differentiation stage were averaged and BIRD was applied to predict DH before and after differentiation. To predict differential DH, we first obtained DHSs that were active in either iPSCs or iPSC-derived neurons. This was done by calculating the mean and standard deviation of the predicted DH (at log2 scale) of all DHSs in each cell type and only retaining DHSs with predicted DH larger than the mean plus one standard deviation in each cell type. For each retained DHS, the differential DH δ was calculated as the difference in the predicted value between the two cell types. DHSs were ranked based on the predicted differential DH.

To test prediction performance, we stratified DHSs according to the absolute value of the predicted DH difference ($|\delta|$) and randomly sampled 12 high-ranked DHSs ($|\delta|>2$), 10 middle-ranked DHSs ($1<|\delta|\leq2$), and 4 low-ranked DHSs ($0.1<|\delta|\leq1$). As negative controls, 5 non-differential DHSs ($|\delta|<0.1$) were randomly chosen (**Supplementary Data 8**). For each of these DHSs, ChIP-qPCR analysis for histone modification mark H3K4me1 was done, and the difference in H3K4me1 between two cell types were calculated as described in the ChIP-qPCR protocol above. All tested DHSs were sorted based on the absolute value of the H3K4me1 difference $|\delta_{K4}|$. DHSs were labeled as validated if their $|\delta_{K4}|$ were larger than the maximal $|\delta_{K4}|$ of the 5 non-differential DHSs (i.e., the dashed line in **Fig. 7a**) and the direction of the predicted DH difference was consistent with the direction of the qPCR difference (i.e., the sign of δ and the sign of δ_{K4} were consistent). To generate **Fig. 7c**, we plotted the predicted DH difference δ versus the ChIP-

qPCR measured H3K4me1 difference at log2 scale (i.e., dCt_{neuron} - dCt_{iPSC} where dCt for a condition is calculated as $dCt = -(Ct_{avg} H3K4me1\ IP\ - Ct_{avg} IgG)$.

Based on the ChIP-qPCR, DHSs with predicted $|\delta|>1$ had good empirical validation rate. Using $|\delta|>1$ as cutoff, BIRD identified 76,495 differential DHSs. To test if differential DHSs predicted by BIRD are associated with differential genes, we first performed differential gene expression analysis by comparing the 2 iPSC exon array samples with the 2 iPSC-derived neuron exon array samples using limma[24]. Genes with adjusted p-value < 0.01 were considered as differential genes (N=4452) and the remaining genes were considered as non-differential genes (N=14,072). We then annotated each DHS with its closest RefSeq gene using CisGenome. We calculated the percentage of differential DHSs among all the DHSs located in the neighborhood (defined as ±10kb, ±5kb, or ±1kb regions from the transcription start site) of each differentially expressed gene. Similarly, we calculated the percentage of differential DHSs among all DHSs located in the neighborhood of each non-differential gene. **Supplementary Fig. 35** compares the mean percentage of differential DHSs in differential genes versus the mean percentage of differential DHSs in non-differential genes. For each neighborhood definition (i.e., ±10kb, ±5kb, or ±1kb TSS regions), we tested whether the mean percentages were different for differential and non-differential genes using one-sided Wilcoxon rank-sum test. For all cases, we obtained p-values < 10^{-15}.

The results show that differential DHSs predicted by BIRD were enriched in flanking regions of differentially expressed genes. Among DHSs located in the ±10kb, ±5kb, and ±1kb regions from the transcription start sites of differentially expressed genes, on average 5.3%, 4.7%, and 3% were differential DHSs, respectively. This represents an enrichment of 1.5, 1.7, and 2.1-fold (all p-values < 10^{-15} by one-sided Wilcoxon rank-sum tests) respectively compared to the percentage of differential DHSs (3.5%, 2.8%, and 1.4%) in non-differential genes (**Supplementary Fig. 35**).

We further analyzed the biological functions of the differentially expressed genes associated with differential DHSs. Each differential ($|\delta|>1$) DHS was annotated with its closest RefSeq gene using CisGenome. From the annotated genes, we extracted differentially expressed genes. Functional annotation analysis was run on these genes using DAVID[15, 16], where the background was set to be all RefSeq genes in exon arrays. Enriched functional terms with FDR ≤0.1 and Fold Enrichment ≥2 reported by DAVID are listed in **Supplementary Data 9**. Consistent with the neuronal differentiation nature of the system, differentially expressed genes associated with differential DHSs were enriched in neuron development and neuron differentiation functions (**Supplementary Data 9**).

To identify enriched TF binding motifs in the differential DHSs, we classified differential DHSs ($|\delta|>1$) into up- ($\delta>0$) or down-regulated ($\delta<0$) DHSs after differentiation. For computational efficiency, the top 3000 ranked DHSs in each class were used in the subsequent analysis. For each class, CisGenome was used to analyze the enrichment of the 1044 human and mouse motifs obtained from TRANSFAC[6] and JASPAR[7]. Given a class of differential DHSs, CisGenome first

generates a list of matched genomic control regions whose distances to genes' transcription start sites match those of differential DHSs. It then computes the enrichment level of each motif as the ratio of the motif's occurrence rate (i.e., # of motif sites per base pair non-repeat sequence) in differential DHSs to its occurrence rate in matched control regions. For each motif, statistical significance of the enrichment was evaluated using one-sided Fisher's exact test (using the numbers of motif sites and the numbers of non-repeat base pairs in differential DHSs and control regions). *P*-values were adjusted using the BH procedure to obtain FDR. Motifs with FDR < 0.05 and enrichment level >2 and with at least 50 motif sites in differential DHS regions were reported as enriched motifs (**Supplementary Data 10)**.

Using predictions to improve regulome data analyses

Among the 17 test cell types used in **Figs 2** and **3**, 16 had duplicate DNase-seq samples and were used for testing whether BIRD can be used to improve DNase-seq data analysis (**Fig. 8a-c**). One cell type ("HPACE") was excluded from this analysis since it did not have replicate samples. Each sample had a data quality score provided by ENCODE[25]. For each test cell type, the sample with higher quality score was reserved to serve as the "truth", and the sample with lower quality score was used as the "observed" data (obs-only). BIRD models trained using the 40 training cell types were applied to predict DH for 912,886 DHSs in each test cell type as described above. For each cell type, the predicted DH profile and the "observed" DH profile were averaged to provide a consolidated DH profile (BIRD+obs). Both obs-only and BIRD+obs can be used to estimate the true DH profile. Their performance was evaluated by computing the cross-locus Pearson's correlation between the truth and the estimated DH profile. Red dots in **Fig. 8c** compare the correlation coefficients obtained by the two methods. Dots above the 45° line represent test cell types in which BIRD+obs outperformed obs-only. As a comparison, the same analysis was repeated after replacing the BIRD predicted DH profile with the mean DH profile of the 40 training cell types (Mean+obs). Blue dots in **Fig. 8c** compare Mean+obs versus obs-only.

To test whether BIRD can be used to improve ChIP-seq data analysis (**Fig. 8d-f**), similar analyses were performed for 9 TFs (ELF1, GABPA, MAZ, NFYB, NRF1, SP1, TCF3, USF1, and YY1) in GM12878 and 3 TFs (ELF1, GABPA and MAZ) in K562 cell lines. ChIP-seq data for these test TFs were downloaded from the ENCODE website (http://hgdownload.cse.ucsc.edu/goldenPath/hg19/encodeDCC/wgEncodeHaibTfbs/ and http://hgdownload.cse.ucsc.edu/goldenPath/hg19/encodeDCC/wgEncodeSydhTfbs/). For each test ChIP-seq dataset, one replicate sample was reserved as the "truth", the other replicate sample was used as the "observed" data. Here, the data quality of each replicate sample was unknown, therefore we used "replicate 1" labeled by ENCODE as test and used "replicate 2" labeled by ENCODE as truth. For each TF, DNA motif obtained from TRANSFAC[6] or JASPAR[7] (**Supplementary Data 3**) was computationally mapped to the human genome using CisGenome[8] (using default likelihood ratio ≥ 500 cutoff). DHSs overlapped with the motif sites were retained for the following analyses. BIRD models trained

using the 40 (or 57) training cell types were applied to predict DH at these sites in GM12878 (or K562). The predicted DH and the observed DH were averaged to obtain the consolidated signal at the retained sites. The correlation between the truth and obs-only at the retained sites was compared with the correlation between the truth and BIRD+obs in **Fig. 8f** (red dots for GM12878, green triangles for K562). For comparison, we also compared Mean+obs with the obs-only (blue dots for GM12878, yellow triangles for K562) in **Fig. 8f**. BIRD+obs outperformed obs-only in most test cases (9 out of 12 TFs).

Supplementary Note 1. Comparing BIRD with other prediction algorithms

To evaluate BIRD, we first compared the locus-level model $\mathrm{BIR}(\overline{\mathrm{X}}, \mathrm{Y})$, which is a building block of BIRD, with a number of prediction algorithms described below.

Lasso[26]: Lasso was performed using the R package "glmnet"[27] where λ was selected by using the minimum mean cross-validated error in model fitting.

Linear regression with stepwise predictor selection[28] *(SPS)*: SPS was implemented based on a greedy forward stepwise variable selection procedure to select *N* predictors that gave the minimum mean squared error in multiple linear regression. For each genomic locus, it first chooses the predictor from all predictors that results in the best linear regression fit to the DH of that locus. In each subsequent iteration, one predictor is added to the regression model by choosing the predictor that provides the greatest reduction in mean squared error of the regression. The procedure stops when *N* predictors are included in the model. The final regression model is used for prediction.

K-nearest neighbors[29] *(KNN)*: KNN was performed using the R package "FNN"[30]. Given a test cell type, the KNN algorithm finds *N* training cell types that are closest to the test cell type according to the similarity in gene expression profile (similarity defined using Euclidean distance). The predicted value for the test cell type was calculated as the average of the DH from its *N* nearest neighbors.

Random forests[31] *(RF)*: RF was performed using the R package "`randomForest`"[32] with default parameters (e.g., number of tree to grow is set to 500 and the minimum depth of tree is set to 5).

Group lasso[33]: Group lasso was performed using the R package "grpreg"[34]. Predictors were clustered using k-means clustering as in BIRD. A group penalty is then applied to the predictors clustered together. For different values of λ, the penalty tuning parameter, the clustered predictors will be selected in or out of the model as a group. The tuning parameter λ was selected by finding the value that minimizes cross-validation squared error loss from 5-folds cross validation in model fitting.

Composite MCP[35]: Composite MCP is another group selection method that is based on a group version of MCP[36] instead of lasso. It was performed using the R package "grpreg"[34]. Groups of predictors were defined using k-means clustering as in BIRD. For different values of λ, γ_1 and γ_2, the penalty tuning parameters, the clustered predictors will be selected in or out of the model as a group. The tuning parameter λ was selected by finding the value that minimizes cross-validation squared error loss from 5-folds cross validation in model fitting. The tuning parameters γ_1 and γ_2 were set to three, the default in "grpreg".

Fused lasso[37]: Fused lasso models were estimated using the R package "penalized"[38]. This approach assumes that predictors are ordered and neighboring predictors have similar regression coefficients. Complete linkage hierarchical clustering was performed on the predictors to order the predictors. The tuning parameters were selected as above using minimum mean cross-validation error from cross validation in model fitting.

Principal component regression[39] (PCR): Principal components (PCs) of the gene expression data were computed. PCs transformed the original predictor vector (i.e., 18,000+ genes) into a lower dimensional predictor vector whose dimension was bounded by the number of training cell types (which is much smaller than the number of genes). Then the Lasso model, as described above, was applied using the principal components as predictors.

The prediction performance of each prediction algorithm was evaluated on a set of randomly chosen DHSs (*n*=9128, representing approximately 1% of the 912,886 DHSs obtained from the 40 training cell types used in the main article). Five-fold cross-validation was performed using 40 cell types. For all methods except for fused lasso and principal component regression (PCR), genes were clustered into *K* clusters. For the group variable selection methods including group lasso and composite MCP, the clusters define the group structure of predictors. For the other methods, the clusters are used to reduce predictor dimension and cluster means are used as predictors. For each of these methods, different *N* (i.e., # of selected predictors) and *K* (# of clusters) settings were tested. Unless otherwise specified, *N* was set to 1, 2, …, 8, and *K* was set to 100, 200, 500, 1000, 1500, 2000 and no clustering. The optimal (*N*, *K*) was determined based on which (*N*, *K*) combination yielded the maximum mean r_C in 5-fold cross-validation in the training cell types. Here we use r_C to determine the optimal (*N*, *K*) because cross-cell-type prediction is the most difficult prediction task. For lasso, we only tested different *K*, since *N* was determined automatically by the lasso algorithm. For SPS, we only considered *K* up to 2000, and *K* with no clustering was not considered because SPS required extremely long computational time. For KNN, *N* represented the number of nearest neighbors. For RF, *N* was determined automatically and therefore we only tested different *K*. Due to heavy computation, *K*=2000 and no clustering were not considered for RF. For group lasso and composite MCP, we only tested different *K* since *N* was determined automatically. For fused lasso, we were not able to run it directly using the 18,000+ predictors due to its model and computational complexity. Thus, we also clustered genes into *K* clusters and used cluster means as predictors to run fused lasso. Here *K* was set to 100, 200 and 500, since for computational reasons we failed to run fused lasso using *K* equal to 1000

or larger. Fused lasso determines *N* automatically. Principal component regression uses principal components instead of clustering to reduce the predictor dimension. Thus, the cluster number *K* is irrelevant here. *N* was determined automatically by lasso which used all principal components as its input.

In addition to prediction accuracy, we also compared the computational time of different methods. For each method, two types of computational time were recorded: (1) T_1 represents the total time spent in the above analyses on searching for the optimal parameters (*N* and *K*) through cross-validation using 1% (9128) of all loci; (2) T_2 represents the total time spent on building prediction models and making predictions using the optimal (*N*, *K*) for the 1% of all loci. The time for applying each method to the whole genome was then estimated as $T = T_1 + 100\ T_2$. This is an estimate of the time required for optimizing *N* and *K* using 1% of loci and then building models and making predictions for all 912,886 (100%) loci using the optimal *N* and *K*. The reason that we estimate the whole-genome application time rather than recording the actual time is because some methods are very slow and require extremely long time for whole-genome application. Values shown in **Supplementary Fig. 5d-f** represent the log2 transformed time (hours), after adding a pseudocount of 1, for each method.

Supplementary Fig. 5a-c shows the prediction accuracy of different methods. **Supplementary Fig. 5d-f** shows T_1, T_2 and T respectively. In terms of prediction accuracy, $\mathrm{BIR}(\overline{\mathrm{X}}, \mathrm{Y})$ was one of the best. Although SPS provided comparable prediction accuracy (**Supplementary Fig. 5a-c**), it is computationally inefficient and is >100 times slower than $\mathrm{BIR}(\overline{\mathrm{X}}, \mathrm{Y})$ (**Supplementary Fig. 5d-f**). Fused lasso also provided comparable r_L and τ (**Supplementary Fig. 5a,c**). However, it performed worse than $\mathrm{BIR}(\overline{\mathrm{X}}, \mathrm{Y})$ in terms of r_C (**Supplementary Fig. 5b**) and it was significantly (>100,000 times) slower than $\mathrm{BIR}(\overline{\mathrm{X}}, \mathrm{Y})$ (**Supplementary Fig. 5d-f**). Among the other methods, KNN, group lasso, composite MCP and PCR had lower prediction accuracy, and lasso, RF, group lasso, composite MCP and PCR were substantially slower than$\mathrm{BIR}(\overline{\mathrm{X}}, \mathrm{Y})$. KNN is the only method that showed comparable computational efficiency as $\mathrm{BIR}(\overline{\mathrm{X}}, \mathrm{Y})$. However, KNN had lower prediction accuracy based on the 1% tested loci. Balancing the overall prediction accuracy and computational efficiency, $\mathrm{BIR}(\overline{\mathrm{X}}, \mathrm{Y})$ provided the best performance.

Since KNN and $\mathrm{BIR}(\overline{\mathrm{X}}, \mathrm{Y})$ had comparable computational efficiency, we further compared their prediction accuracy using all 912,886 DHSs rather than 1% of random DHSs. To this end, we trained both KNN and $\mathrm{BIR}(\overline{\mathrm{X}}, \mathrm{Y})$ using the 40 training cell types, with optimal parameters determined by five-fold cross-validation using the training data. The trained models were then applied to predict DH in the 17 test cell types. The prediction accuracy is shown in **Supplementary Fig. 6**. Consistent with the results obtained using 1% of random DHSs, $\mathrm{BIR}(\overline{\mathrm{X}}, \mathrm{Y})$ showed higher prediction accuracy than KNN in this whole-genome comparison. This is not surprising because based on the statistical sampling theories, a population characteristic can be unbiasedly estimated using a random sample representative of the population.

Since $\mathrm{BIR}(\overline{\mathrm{X}}, \mathrm{Y})$ provided the best performance among all the compared methods, we chose it as the basic building block for constructing more complex models. Compared to $\mathrm{BIR}(\overline{\mathrm{X}}, \mathrm{Y})$, the final locus-level prediction by BIRD based on model aggregation offers higher prediction accuracy (**Supplementary Fig. 4**). However, the BIRD model requires extra time for clustering the DHSs and making cluster-level predictions. We compared the computational time of $\mathrm{BIR}(\overline{\mathrm{X}}, \mathrm{Y})$ and BIRD based on applying them to the whole genome. Here we did not estimate the time based on 1% of DHSs because clustering DHSs requires all 912,886 loci. For the BIRD model, it took 9.4 hours to train prediction models for all loci using 40 training cell types. This includes the time for choosing the optimal *N* and *K* for $\mathrm{BIR}(\overline{\mathrm{X}}, \mathrm{Y})$ using 1% of loci (these *N* and *K* are then used for both $\mathrm{BIR}(\overline{\mathrm{X}}, \mathrm{Y})$ and $\mathrm{BIR}(\overline{\mathrm{X}}, \overline{\mathrm{Y}})$), clustering DHSs into 1000, 2000 and 5000 clusters, training all locus-level and cluster-level prediction models for all 912,886 loci, and computing the weights for model averaging. The $\mathrm{BIR}(\overline{\mathrm{X}}, \mathrm{Y})$ fitting time was 8.1 hours (including the time for choosing the optimal *N* and *K* using 1% of loci and training all 912,886 locus-level prediction models using the optimal *N* and *K*) and thus the increased prediction accuracy from the BIRD model comes with a less than twenty percent increase in computational time (9.4/8.1=1.2). By contrast, our previous analysis in **Supplementary Fig. 5d-f** shows that SPS, random forest, lasso, group lasso, composite MCP and fused lasso are all at least 10 times slower than $\mathrm{BIR}(\overline{\mathrm{X}}, \mathrm{Y})$, and PCR is 9 times slower than $\mathrm{BIR}(\overline{\mathrm{X}}, \mathrm{Y})$. Thus, the BIRD model is still computationally efficient. Once the prediction models are trained, all fitted model parameters will be stored for future use. Applying the stored BIRD models to make predictions on ~1 million loci in 100 new samples took less than 2 minutes. Thus, the prediction component of BIRD requires little time. Here, all computational time were measured using a computer with 2.5 GHz CPU and 10Gb RAM.

Supplementary Note 2. Analysis of predictors selected by BIRD

To study the relationship between the DHSs and their predictor genes, we analyzed 1,108,603 DHSs in the BIRD model trained by 57 cell types. First, each DHS was annotated with its closest RefSeq gene using CisGenome[8]. Then, according to the distance from the center of a DHS to the transcription start site (TSS) of its closest gene, we defined the promoter DHSs as DHSs located in genomic regions between upstream 1kb to downstream 1kb of TSSs. Each DHS has multiple gene clusters as predictors (this is the union of predictors from the locus-level and pathway-level models), and genes in these clusters were considered as the predictor genes for this DHS.

For each DHS, we examined whether its closest gene was contained in its predictor genes. We then calculated the percentage of DHSs whose closest gene was contained in its predictors. The analysis was performed on all DHSs and on promoter DHSs respectively. As a negative control, we permuted the link between the DHS and its predictors. In other words, we randomly assigned gene clusters as predictors to each DHS, but we kept the number of gene clusters assigned to a DHS unchanged. We then applied similar analysis to compute the percentage of DHSs for which the closest genes were covered by predictors. This random assignment process was run 1000

times. The average percentage and the standard deviation of the percentage were calculated and shown in **Supplementary Fig. 16a-b**. From the 1000 random permutations, we computed the empirical *p*-values using [the number of permutations that generate a percentage ≥ the observed percentage]/1000. The *p*-values were smaller than 0.001 for all DHSs and for promoter DHSs.

For the analysis of enhancer DHSs, FANTOM5[40] human enhancers and their corresponding target genes were obtained (http://enhancer.binf.ku.dk/presets/enhancer_tss_associations.bed). In FANTOM5, enhancers were identified using enhancer RNA obtained from cap analysis of gene expression[41] (CAGE). Target genes of the enhancers were identified based on the pairwise expression correlation between enhancers and genes across different cell types. We identified 28,563 DHSs that overlapped with these enhancers. For each enhancer DHS, we examined whether its target genes were contained in its predictor genes. As a negative control, we randomly assigned the same number of gene clusters as predictors to each enhancer DHS and applied similar analysis. The random assignment was performed 1000 times. Empirical *p*-value was computed similar as above (**Supplementary Fig. 16c**, *p*-value<0.001).

The results show that only 5.2% of all DHSs, 9.8% of promoter DHSs and 13.6% of enhancer DHSs had their closest genes or target genes contained in the predictors. While these represent significant enrichment (7-fold, 13-fold and 7-fold enrichment respectively, permutation test *p*-values < 0.001, **Supplementary Fig. 16a-c**) compared to random expectation, the majority of DHSs did not have their closest genes or target genes chosen as predictors, consistent with the hypothesis that information useful for prediction is not all contained in DHSs' closest or target genes.

Next, we examined pathway-level models to see whether transcription factors that bind to regulatory elements in a pathway are enriched in the pathway's predictors. To this end, we examined the enriched DNA motifs for each DHS pathway and asked whether predictors of the pathway contained TFs that could bind to these motifs. To perform motif enrichment analysis, we obtained 1044 human and mouse motifs from TRANSFAC[6] and JASPAR[7]. Then, the motifs were computationally mapped to the human genome by CisGenome. For each DHS pathway and each motif, the motif enrichment score was calculated using the ratio $r=x/y$, where x = [number of motif sites in target regions/total length of non-repeat sequence in target regions] and y = [number of motif sites in control regions/total length of non-repeat sequence in control regions]. Here, the target regions refer to DHSs in a DHS pathway, and the control regions refer to the control genomic regions randomly chosen by CisGenome to match genomic distributions of the target regions. These control regions were obtained using the "matched genomic controls" function in CisGenome. For each motif, to test whether the motif is significantly enriched in the target regions as compared to the control regions, one-sided Fisher's exact test was applied (using the numbers of motif sites and the numbers of non-repeat base pairs in target regions and control regions). *P*-values were adjusted using Benjamini–Hochberg (BH) procedure to obtain FDR[42]. Motifs that showed FDR < 0.05 and enrichment ratio larger than 2 and had more than 50 motif sites in target regions were labeled as enriched motifs. For each DHS pathway, we then

examined whether its predictor genes contained at least one TF corresponding to the enriched motifs. The percentage of DHS pathways whose predictors contained TFs for enriched motifs was calculated. As a negative control, we randomly assigned gene clusters as predictors to each DHS pathway but kept the number of assigned gene clusters for each pathway unchanged. This random procedure was repeated 1000 times. The empirical *p*-value was then calculated as [the number of permutations that generate a percentage ≥ the observed percentage]/1000. Here, the percentage refers to the percentage of DHS pathways whose predictors contained TFs for enriched motifs. We conducted the analysis after clustering DHSs into 1000, 2000 and 5000 pathways respectively. In all cases, the empirical *p*-values were smaller than 0.001 (**Supplementary Fig. 16d-f**).

For each DHS pathway, we also performed Gene Ontology (GO) analysis on its predictor genes using the R package "topGO"[43]. Each DHS pathway has multiple gene clusters as predictors and each gene cluster contains multiple genes. GO analysis was applied to all genes contained in these gene clusters, and all RefSeq genes in exon arrays were used as background. *P*-values obtained from topGO were adjusted using BH procedure to obtain FDR. Enriched GO terms with FDR < 0.05 were reported.

For each DHS pathway, we annotated it with cell types in which it is active. In order to do so, we first applied one-sided Wilcoxon rank-sum test to evaluate whether the mean DH within the DHS pathway is different from the mean DH value of all other DHSs in each cell type. *P*-values from all cell types were then adjusted using the BH procedure to obtain FDR. For each cell type, we also calculated the DH enrichment of a DHS pathway as the difference (δ) between the average DH value (at log2 scale) within the DHS pathway and the average DH value from all DHSs. According to the FDR and DH enrichment, cell types with FDR < 0.05 and δ>1 were considered as active cell types for each DHS pathway.

We found that TFs that potentially regulate the pathways were enriched in pathways' predictors. For example, consider clustering DHSs into 1000 pathways. We identified enriched DNA motifs for each DHS pathway. For 37.8% of the DHS pathways, at least one TF corresponding to the enriched motifs was contained in the predictors selected by $\mathrm{BIR}(\overline{\mathrm{X}}, \overline{\mathrm{Y}})$. By contrast, if the same number of predictors were randomly assigned to each DHS pathway, only 9.9% of the DHS pathways would have their predictors covering at least one TF for the enriched motifs (37.8%/9.9% = 3.8-fold enrichment). Clustering DHSs into 2000 or 5000 pathways yielded similar results (**Supplementary Fig. 16d-f**). Since we used clusters of co-expressed genes as predictors, having a TF in the predictor implies that genes co-expressed with the TF, which often come from related biological processes or pathways, are also included in the predictors. **Supplementary Fig. 16g-j** and **Supplementary Data 2** show a sample DHS pathway which is active in human skeletal muscle myoblasts (HSMM) and skeletal muscle myotubes differentiated from HSMM (HSMMtube). TFs known to be involved in muscle development such as *MYF6*, *MYOD1* and *MYOG* were found in the pathway's predictor genes (**Supplementary Fig. 16i**), and motifs of these TFs were among the top enriched motifs in the DNA sequences of DHSs in this pathway (**Supplementary Fig. 16h**).

Gene Ontology (GO) analysis of the predictor genes further identified “muscle cell development” and “skeletal muscle tissue development” as enriched GO terms for this pathway (**Supplementary Fig. 16j**). Thus, predictor genes of this pathway were enriched in biological processes consistent with the cell types in which the DHS pathway is active.

The same analyses were also conducted on other pathways. The motif enrichment analysis results, GO analysis results, active cell types for each DHS pathway, and predictor genes for each DHS are all provided as an online resource available at https://zhiji.shinyapps.io/CABS/. Users can input a list of genomic regions (a bed file) and explore the predictors for each DHS within the input regions.

Supplementary Note 3. Relationship between conservation of DHSs and predictor genes

We asked whether BIRD selected predictors for phylogenetically conserved DHSs were also phylogenetically conserved. To answer this question, we first calculated the sequence conservation score for each DHS using CisGenome[8]. For each DHS, the conservation is measured using the mean phastCons[44] score across all positions within the DHS (phastCons 46-way conservation score for vertebrate genomes was used; the score was linearly scaled to 0-255 by CisGenome). We then stratified DHSs into four groups according to the quartiles of their conservation scores.

Next, we measured the sequence conservation and functional conservation for each gene. The sequence conservation of a gene was calculated using the mean phastCons score of its exonic positions. To measure genes’ functional conservation, we obtained Affymetrix human and mouse exon array gene expression data from a collection of matching tissue types (http://www.affymetrix.com/support/technical/sample_data/exon_array_data.affx). For each gene, we obtained human and mouse orthologs from the Mouse Genome Informatics database (download link: http://www.informatics.jax.org/downloads/reports/HOM_MouseHumanSequence.rpt). The functional conservation of the gene was then measured using the Spearman’s rank correlation of its expression between human and mouse orthologs across all matching tissues.

For each DHS, the mean sequence conservation of all its predictor genes (predictor genes are the union of genes from the locus-level and pathway-level models) was computed to measure its predictors’ overall sequence conservation. Similarly, the mean functional conservation of all its predictor genes was computed to measure its predictors’ overall functional conservation. We then compared the predictors’ sequence and functional conservation among the four groups of DHSs with different conservation levels. As a control, we also randomly sampled a quarter of all DHSs and applied the same analysis. **Supplementary Fig. 17a** shows that there was no substantial difference in the predictors’ sequence conservation for DHSs at different conservation levels.

Although predictors for the most conserved DHSs tended to be slightly more conserved in their DNA sequences, the magnitude of the difference is quite small. In terms of predictors' functional conservation, **Supplementary Fig. 17b** shows that there was no clear difference for DHSs at different conservation levels.

Supplementary Note 4. TFBS prediction using improved motif model

TFBS prediction involves integration of multiple types of information. This article is focused on discussing chromatin accessibility. There is still plenty of room for improving TFBS prediction accuracy by optimizing the use of other information types. For example, one lesson learned from the recent ENCODE-DREAM Challenge on in vivo Transcription Factor Binding Site Prediction (https://www.synapse.org/#!Synapse:syn6131484/wiki/402031) is that modeling intra-motif correlation (i.e., correlation among different positions within a motif) can improve TFBS prediction accuracy. To demonstrate that there is room for improving TFBS prediction by BIRD, we predicted ELF1 TFBSs using a more sophisticated ELF1 motif model that accounts for intra-motif correlation. A number of high-throughput technologies such as ChIP-seq and protein binding microarray (PBM)[45] have generated rich TF binding data which can be used to model intra-motif correlation. Previous studies also suggest that for many TFs, modeling intra-motif correlation can increase TFBS prediction accuracy[46-48]. Here we consider a scenario where ChIP-seq data for a TF are available for a few cell types. Using these ChIP-seq data, one can derive an enhanced motif model that accounts for the intra-motif correlation. This enhanced motif model is then be applied to new cell types to predict TFBSs.

We build the enhanced motif model as follows. For a given TF, suppose one has its ChIP-seq data from several training cell types. These training cell types do not need to have DNase-seq data. Starting with an initial motif model that assumes independence among positions within the motif (i.e., a traditional position specific weight matrix (PWM)), our goal is to build an improved motif model that allows intra-motif correlation.

Step 1: The initial motif (PWM) of the TF is mapped to genome using CisGenome (using the default likelihood ratio cutoff of 500). The motif mapping score (log likelihood ratio) for each mapped motif site is recorded.

Step 2: If a motif site overlaps with ChIP-seq peak in at least one training cell type, it is marked as positive. Otherwise it is marked negative. Typically, the number of negative motif sites is much larger than the number of positive motif sites.

Step 3: A positive training set and a negative training set are prepared by randomly sampling motif sites with matching motif scores. To do this, motif sites were stratified into 10 equal-sized groups based on the 10th, 20th, ..., 100th percentiles of their motif mapping scores. For each stratum, we subsample the negative sites so that the number of negative motif sites and the number of positive motif sites are the same, since the number of negative motif sites is usually

much larger than the number of positive motif sites. However, if there are more positive motif sites than negative motif sites, we subsample the positive sites instead. After motif sites are sampled from each stratum, all sampled motif sites are pooled together to form the positive and negative training sets.

Step 4: For each motif site in the training sets, we construct a feature vector that encodes the dinucleotides observed at all pairs of positions. For example, consider a motif site CACGTG. For position 1 and position 2, we use 16 binary indicators (x_{1A2A}, x_{1A2C}, x_{1A2G}, x_{1A2T}, …, x_{1T2A}, x_{1T2C}. x_{1T2G}, x_{1T2T}) to model the 16 possible dinucleotides. Since the observed dinucleotide at positions 1 and 2 is CA, the indicator x_{1C2A} is equal to 1 and all other indicators are equal to 0. Similarly, we model all position pairs (i.e., positions 1-2, 1-3, 1-4, …, 2-2, 2-3, …) and concatenate their binary indicators into one vector. After we convert all motif sites into feature vectors, features (i.e., binary indictors) informative for discriminating positive and negative motif sites are selected. To do so, for each feature x_{iLjM} (i, j represent positions; L, M represent nucleotides), we compute the percentage of motif sites in the positive training set that carry the corresponding dinucleotide (i.e., percentage of positive motif sites with x_{iLjM}=1) and the percentage of motif sites in the negative training set that carry it. The feature is retained if the absolute value of the difference between the two percentages is larger than 0.05. Using all retained features, a random forests (RF) model (number of trees to grow is set to 1000) is trained using the positive and negative training motif sites. When applying this RF model to a new motif site, it will predict the TF binding probability of this site. This prediction is primarily based on the intra-motif correlation because the RF model is trained using positive and negative motif sites with matched PWM scores. The matching removes the information carried by PWM for discriminating positive and negative motif sites.

Step 5: Next, we use the RF model obtained in **Step 4** ("RF1") to enhance the original PWM-based motif model (which is conceptually similar to the idea of boosting). To do so, we use all positive motif sites as the positive training set, and we construct another equal-sized negative training set by randomly sampling negative motif sites. The RF model obtained in **Step 4** (RF1) is used to score all these motif sites. Note that the new negative training set is not matched with the positive training set in terms of PWM score. Therefore, both the intra-motif correlation model RF1 and the initial PWM model contain information for discriminating the positive motif sites and negative motif sites in the new training set (where negative motif sites are random). Using these new training data, we train a model that predicts TF binding by aggregating two models: the PWM model and the RF1 intra-motif correlation model. This is done by training another RF (number of trees = 1000) that uses the PWM score and the RF1 score as two features for each motif site. We use "RF2" to denote this new RF model.

RF2 is our final motif model. It will be applied to rescore all motif sites in the genome obtained in **Step 1**. The new scores will replace the old PWM-based motif mapping scores to rank motif sites. This will not change the set of motif sites, but it will change the motif site ranking. For example, motif sites may move up in the ranking if their intra-motif correlation matches the

correlation structure learned from the training data. By contrast, motif sites with similar PWM scores may receive lower ranking if their intra-motif correlation structure is not consistent with the correlation structure of the positive motif sites in the training data.

For ELF1, we trained this new motif model using ELF1 ChIP-seq data from A549, HepG2 and K562 cells. We then applied it to help us predict ELF1 binding sites in a new cell type GM12878. To do so, we first rescored all ELF1 motif sites using RF2 and re-ranked all motif-containing DHSs based on the new motif scores. Here the motif score for each DHS was defined as the maximal RF2 motif score of all motif sites within the DHS. Next, we also ranked all motif-containing DHSs based on BIRD-predicted DH. We then computed the average of the RF2-motif-based rank and the BIRD-predicted-DH-based rank. All motif-containing DHSs were rescored and ranked using this average rank. This new scoring approach is denoted using BIRD+RF2. **Supplementary Fig. 30** shows that the new scoring approach BIRD+RF2 outperformed the original BIRD prediction (denoted as BIRD) which ranked motif-containing DHSs based on the predicted DH value. We asked whether this improvement was due to the use of intra-motif correlation or simply because we added the motif-based rank into the scoring scheme. To answer this question, we repeated the BIRD+RF2 analysis but used PWM motif score to replace RF2 motif score. In other words, we ranked all motif-containing DHSs based on their original PWM motif scores and then rescored each DHS using the average of its PWM-based rank and the BIRD-predicted-DH-based rank (denoted as BIRD+PWM). Motif-containing DHSs were then ranked based on this BIRD+PWM score. BIRD+PWM and BIRD+RF2 were different only in the way they score motif sites. These two methods were the same otherwise. BIRD is different from BIRD+PWM in that BIRD ranks motif-containing DHSs based on the predicted DH only, whereas BIRD+PWM ranks motif-containing DHSs based on a score that combines the predicted-DH-based rank and the motif-score-based rank. **Supplementary Fig. 30** shows that BIRD outperformed BIRD+PWM. Thus, the improvement of BIRD+RF2 over BIRD is due to the use of intra-motif correlation rather than simply adding the motif-based rank into the scoring scheme (because adding PWM-based rank decreased performance). Finally, for top ranked predictions, BIRD+RF2 also outperformed predictions based on ranking motif-containing DHSs using the true DNase-seq data (True). At the 10%, 25% and 50% FDR level, the sensitivity of BIRD+RF2 was 0.51, 0.75 and 0.88. By contrast, the sensitivity of the original BIRD prediction was 0.43, 0.64 and 0.88, and the sensitivity of the prediction based on true DNase-seq was 0.27, 0.64, and 0.94.

This analysis demonstrates that there is plenty of room for improving TFBS prediction accuracy by optimizing the use of other information types. The method used above for incorporating intra-motif correlation requires one to have training data to train the intra-motif correlation model. There are many other types of information such as phylogenetic conservation, motifs of collaborating TFs, and genomic locations relative to genes that may improve TFBS prediction. However, exploring the optimal use of all these non-DH components and building the optimal TFBS prediction pipeline are beyond the scope of this article and will be addressed elsewhere.

Supplementary Note 5. Using predictions for data quality check

We investigated whether the correlation between BIRD-predicted DH and experimental DNase-seq data can be used to check data quality. We downloaded 66 ENCODE DNase-seq samples (**Supplementary Data 11**) not included in our original training and test data (i.e., not in the DNase-seq samples of the 57 cell types mentioned in the main article) in bam format (download link:http://hgdownload.cse.ucsc.edu/goldenPath/hg19/encodeDCC/wgEncodeOpenChromDnase/). The samples used for this analysis and the samples used in the main article for building BIRD models were generated by two different laboratories. They were processed consistently as described in the **Methods** section **"DNase-seq data processing"**. Gene expression exon array data for the same samples were downloaded from GEO (GEO accession number: GSE15805) and processed consistently as described in the **Methods** section **"Gene expression data processing"**.

For each of the 66 DNase-seq samples, we computed the Pearson's correlation between its measured DH and BIRD-predicted DH (using the exon array data from the corresponding cell type) across all DHSs. Here the BIRD models were trained using the 57 cell types used in the main article. We then asked whether this correlation, termed "BIRDcor", can be used as a quality control (QC) metric. To this end, we compared BIRDcor with the QC metrics used by ENCODE for these samples, including the total number of uniquely mapped reads (seq-depth), the fraction of reads that fall in tag-enriched regions (SPOT), the ratio of non-redundant uniquely mapped reads to all uniquely mapped reads (PBC), and correlations between signals from forward and reverse reads (normalized strand cross-correlation (NSC) and relative strand cross-correlation (RSC)). The ENCODE QC metrics for each DNase-seq sample were obtained from https://genome.ucsc.edu/ENCODE/qualityMetrics.html. Each QC metric was used to rank the 66 DNase-seq samples (**Supplementary Data 11**).

In order to compare different QC metrics in terms of their ability to identify poor-quality samples, we first conducted a pairwise comparison between BIRDcor and each ENCODE QC metric. For each pairwise comparison, we compiled a list of poor-quality samples and used them as a gold standard for evaluation. To compile this list, we excluded BIRDcor and the ENCODE QC metric being compared. We then pooled the lowest x% (x=5, 10, and 20) ranked samples identified by each remaining ENCODE QC metric. In other words, we obtained x% samples with the lowest quality for each of the remaining ENCODE QC metric and took their union. We took union because different QC metrics are complementary to each other (see below). The parameter x used to define poor-quality samples was set to 5, 10 and 20 respectively (i.e., pooling the lowest 5%, 10%, and 20% ranked samples). Using these poor-quality samples as gold standard, we compared BIRDcor and the tested ENCODE QC metric in terms of their ability to detect poor-quality samples by computing their receiver operating characteristics (ROC) and sensitivity versus FDR curves (**Supplementary Figs. 36-37**). The results show that BIRDcor had better performance than the ENCODE QC metrics in most of the comparisons. We note that in the sensitivity versus FDR comparisons, all methods had relatively low sensitivity when the FDR was low (**Supplementary Fig. 37**). This is not surprising because different metrics are complementary to each other and

each QC metric can only detect a subset of low quality samples while missing many low quality samples identified by other metrics.

Next, we compared all methods together. For this comparison, gold standard poor-quality samples were defined by pooling the x% (x=5, 10, and 20) samples with the lowest quality identified by each QC metric. In other words, we obtained x% samples with the lowest quality for BIRDcor and each ENCODE QC metric and then took their union. Using these poor-quality samples as gold standard, we then tested the ability of each individual QC metric to detect poor-quality samples. Regardless of the value of x, BIRDcor showed comparable performance to the other QC metrics in terms of both ROC and sensitivity-FDR curve (ROC: **Supplementary Fig. 38a-c**; Sensitivity-FDR: **Supplementary Fig. 38d-f**).

In the above analyses, the gold standard poor-quality samples were defined by pooling x% samples with the lowest quality. We also defined gold standard poor-quality samples in other ways and obtained similar results. For instance, for the PBC, NSC and RSC metric, we defined poor-quality samples in an alternative way using PBC $\leqslant 0.5$, NSC $\leqslant 1.1$, and RSC $\leqslant 1$ as cutoffs (these are cutoffs suggested by ENCODE). For seq-depth and SPOT, ENCODE did not provide suggested thresholds. For these two ENCODE QC metrics and BIRDcor, we obtained the lowest 5%, 10% and 20% ranked samples from each metric. Poor-quality samples identified by different QC metrics were then pooled as before to redefine the gold standard lists of poor-quality samples. Based on the new gold standard definitions, BIRDcor still performed comparable to or better than the other QC metrics (**Supplementary Figs. 39, 40, 41**). Together, these results demonstrate that BIRDcor can serve as a QC metric to help assess data quality.

Supplementary Fig. 42 shows the 7 lowest ($\approx$ lowest 10%) ranked samples identified by BIRDcor and 7 randomly selected samples. Here, each column represents a QC metric. For each QC metric, samples with low quality were highlighted in dark red (rank 1-7, lowest 10%) and light red (rank 8-13, lowest 20%) colors. This figure shows that samples identified by BIRDcor as poor-quality samples are often supported by low QC measurements from one or more other QC metrics. **Supplementary Fig. 42** also shows that different QC metrics are complementary to each other. Poor-quality samples failed to be identified by one QC metric may be identified by others. For instance, Chorion_Rep2 did not look problematic based on seq-depth, SPOT, NSC and RSC, but it was identified by BIRDcor and PBC as a sample with relatively low quality. Some samples did not look problematic based on BIRDcor, but they could be ranked low by other QC metrics. Thus, in practice, we recommend using multiple QC metrics together. These QC metrics often examine samples' quality from different perspectives and use different types of information. Therefore, one would be more confident about the judgement of poor quality if a sample's poor quality is indicated by multiple QC metrics. In this regard, BIRDcor provides an additional piece of information one can use for QC check, and it contributes a new metric to the existing QC toolbox.

Supplementary Note 6. BIRD predictions cover a large fraction of the regulome

Although DHSs in the BIRD prediction model were selected from the training cell types, they cover a large fraction of the regulome. To see this, we gradually increased the number of cell types in the training data and detected DHSs as described in **Methods**. We then plotted the number of unique DHSs discovered in the genome as a function of the number of cell types in the training data. In this analysis, the order in which cell types were added to the training data was random. We repeated this analysis 10 times. **Supplementary Fig. 43a** shows the average and standard deviation of the number of DHSs discovered at different number of cell types. **Supplementary Fig. 43b** further shows how adding a new cell type to training data would contribute to the detection of new DHSs. To generate this figure, we let $Y(n)$ denote the number of DHSs discovered from n training cell types. Each time a new cell type was added to the training data, we counted the number of new unique DHSs (i.e., DHSs not already existing in the training data) introduced by the new cell type $Y(n)- Y(n-1)$. **Supplementary Fig. 43b** plots the fraction of new DHSs contributed uniquely by adding a new cell type, $[Y(n)-Y(n-1)]/Y(n)$, as a function of n (i.e., the number of training cell types). **Supplementary Fig. 43a** shows that the increase in the number of DHSs gradually slowed down as the number of training cell types increased. **Supplementary Fig. 43b** shows that as the number of available cell types in the training data increases, the number of new regulatory elements in any new sample is likely to account for only a small fraction of the regulome. It is expected that more cell types with both regulome and gene expression data will become available in the near future. As such, it is very likely that the DHSs covered by the training data will approach saturation and cover most of the DHSs in the genome.

Supplementary Figures

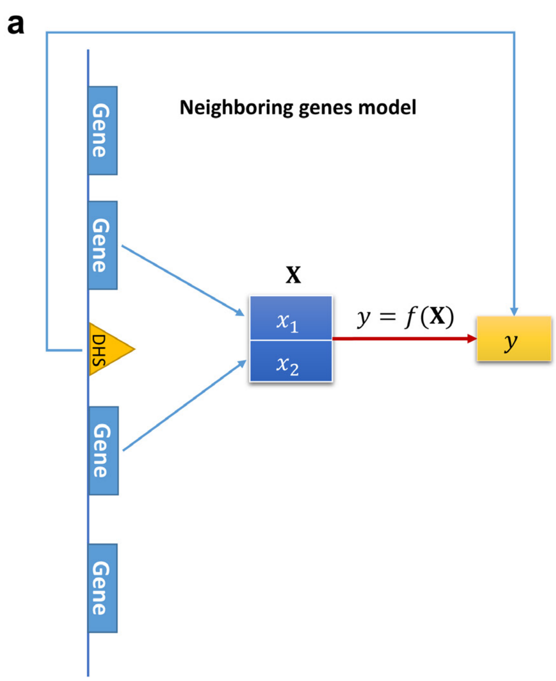

Supplementary Figure 1. Prediction based on neighboring genes versus prediction based on all genes. (**a**) In order to predict DH level of a genomic locus (yellow triangle), the neighboring gene approach extracts neighboring genes of the locus and uses their expression levels as predictors to make predictions. (**b**) Instead of using only neighboring genes, BIRD uses the whole transcriptome (i.e., expression levels of all genes) to build prediction models.

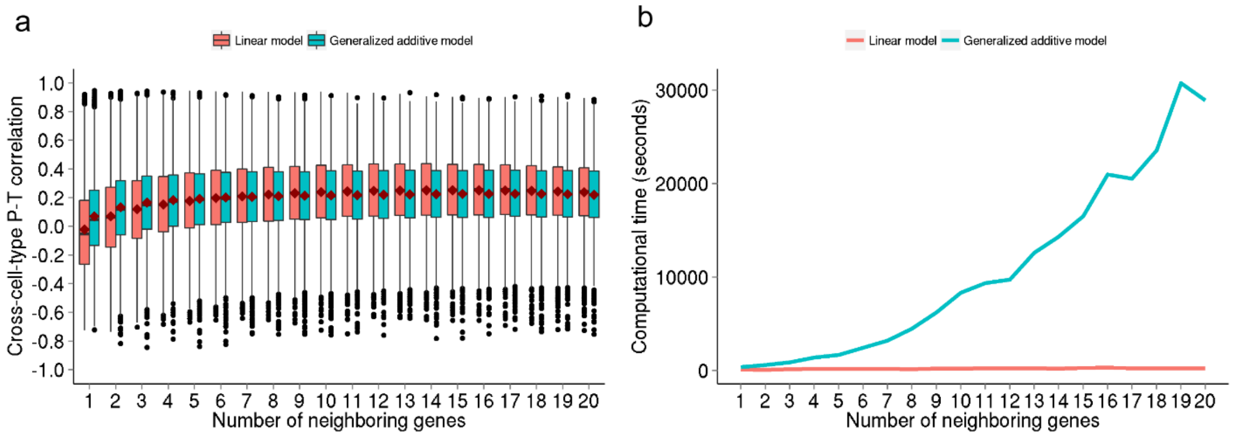

Supplementary Figure 2. Prediction performance by the neighboring gene approach. (**a**) Distribution of cross-cell-type P-T correlation r_C from 1% of randomly chosen loci when different numbers of neighboring genes were used as predictors. Both linear and nonlinear (generalized additive) models were tested. Each boxplot shows the median (central line), interquartile range (IQR, the 1st (Q1) to 3rd (Q3) quartiles, box), and 1.5 × IQR from the Q1 and Q3 (lower and upper whiskers) of the data. The diamond represents the mean. (**b**) Comparison of computational time (seconds) for the linear and nonlinear models using different number of neighboring genes as predictors.

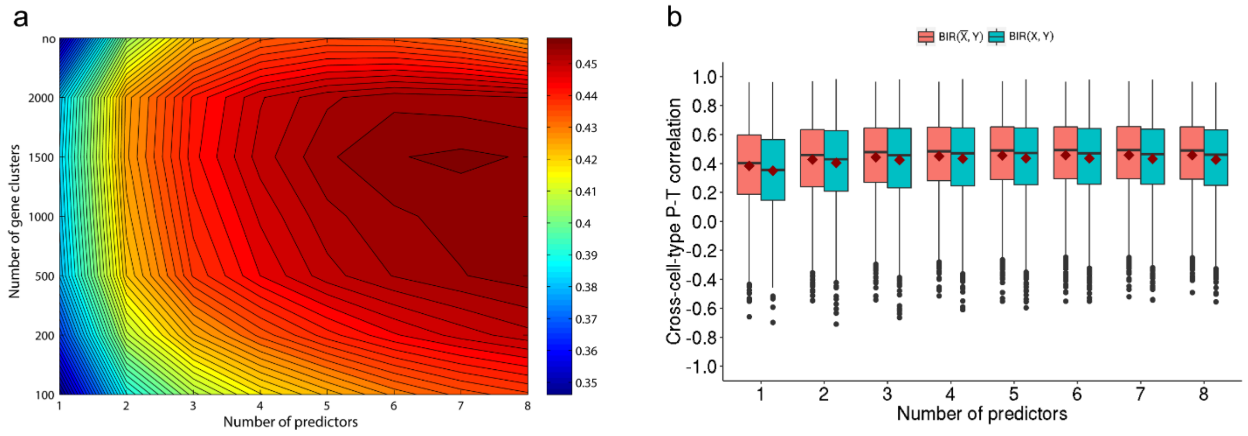

Supplementary Figure 3. Parameter selection for $\mathrm{BIR}(\overline{\mathrm{X}}, \mathrm{Y})$. (**a**) Mean cross-cell-type P-T correlation r_C of 1% random loci in cross-validation (using 40 training cell types) for different combinations of K (the number of gene clusters) and N (the number of predictors). The optimal combination was K=1500 and N=7. On the y-axis, "no" means genes are not clustered (i.e., it corresponds to $\mathrm{BIR}(\mathrm{X}, \mathrm{Y})$) (**b**) Comparison of r_C between $\mathrm{BIR}(\overline{\mathrm{X}}, \mathrm{Y})$ and $\mathrm{BIR}(\mathrm{X}, \mathrm{Y})$ when holding the number of selected predictors (N) the same. For $\mathrm{BIR}(\mathrm{X}, \mathrm{Y})$, N=5 provides the optimal performance. Each boxplot shows the median (central line), interquartile range (IQR, the 1st (Q1) to 3rd (Q3) quartiles, box), and 1.5 × IQR from the Q1 and Q3 (lower and upper whiskers) of the data. The diamond represents the mean.

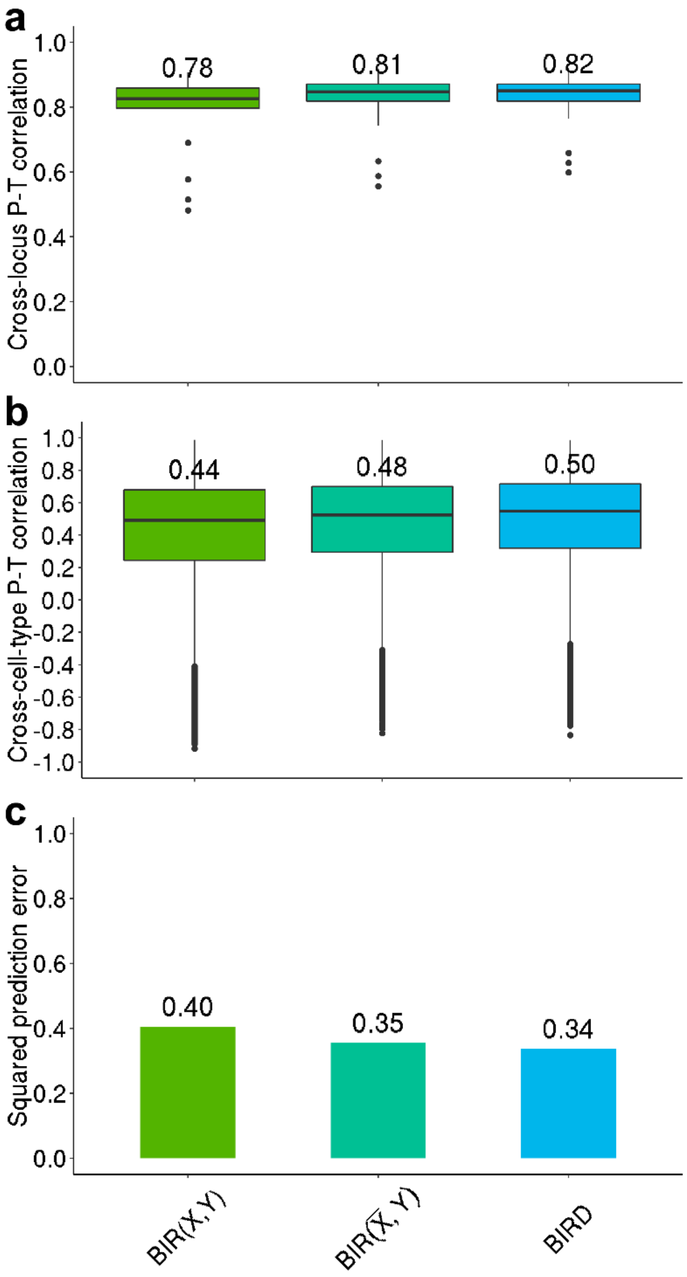

Supplementary Figure 4. Comparison of prediction performance of $\mathrm{BIR}(\mathrm{X},\mathrm{Y})$, $\mathrm{BIR}(\overline{\mathrm{X}},\mathrm{Y})$, and BIRD. $\mathrm{BIR}(\mathrm{X},\mathrm{Y})$ is a special case of $\mathrm{BIR}(\overline{\mathrm{X}},\mathrm{Y})$ where predictors are not clustered. BIRD is the final aggregated model for predicting DH at each locus. (**a**) Cross-locus P-T correlation r_{L}. (**b**) Cross-cell-type P-T correlation r_{C}. (**c**) Squared prediction error τ. In (**a**) and (**b**), each boxplot shows the median (central line), interquartile range (IQR, the 1st (Q1) to 3rd (Q3) quartiles, box), and 1.5 × IQR from the Q1 and Q3 (lower and upper whiskers) of the data. The mean r_{L} and r_{C} for each method are shown.

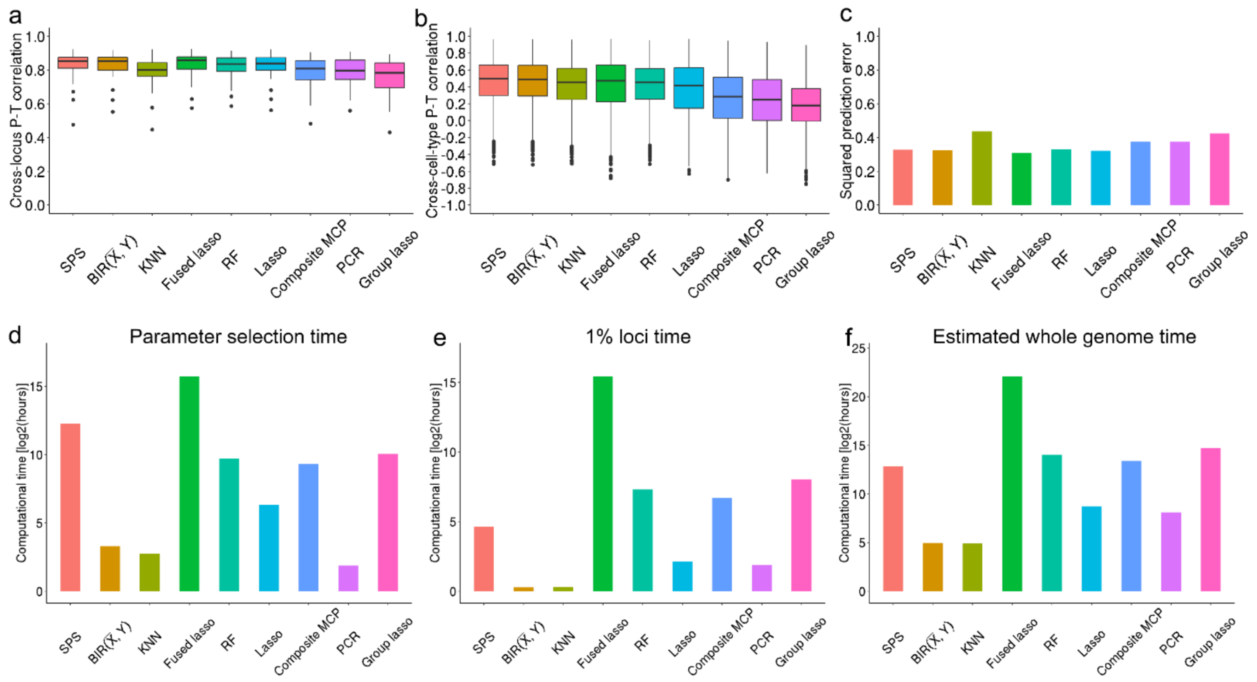

Supplementary Figure 5. Comparison of prediction performance and computational efficiency of different methods based on 1% of random DHSs. Performance accuracy was compared in terms of (**a**) cross-locus P-T correlation r_L, (**b**) cross-cell-type P-T correlation r_C and (**c**) Squared prediction error τ. Computational efficiency was compared in terms of (**d**) the time [log2(hours)] spent on searching for optimal parameters using 1% of loci (T_1), (**e**) the time [log2(hours)] spent on building prediction models and making predictions on 1% of loci using the optimal parameters (T_2), and (**f**) the estimated computational time [log2(hours)] for each method to conduct a genome-wide analysis for performance evaluation ($T = T_1 + 100\ T_2$). In (**a**) and (**b**), each boxplot shows the median (central line), interquartile range (IQR, the 1st (Q1) to 3rd (Q3) quartiles, box), and 1.5 × IQR from the Q1 and Q3 (lower and upper whiskers) of the data.

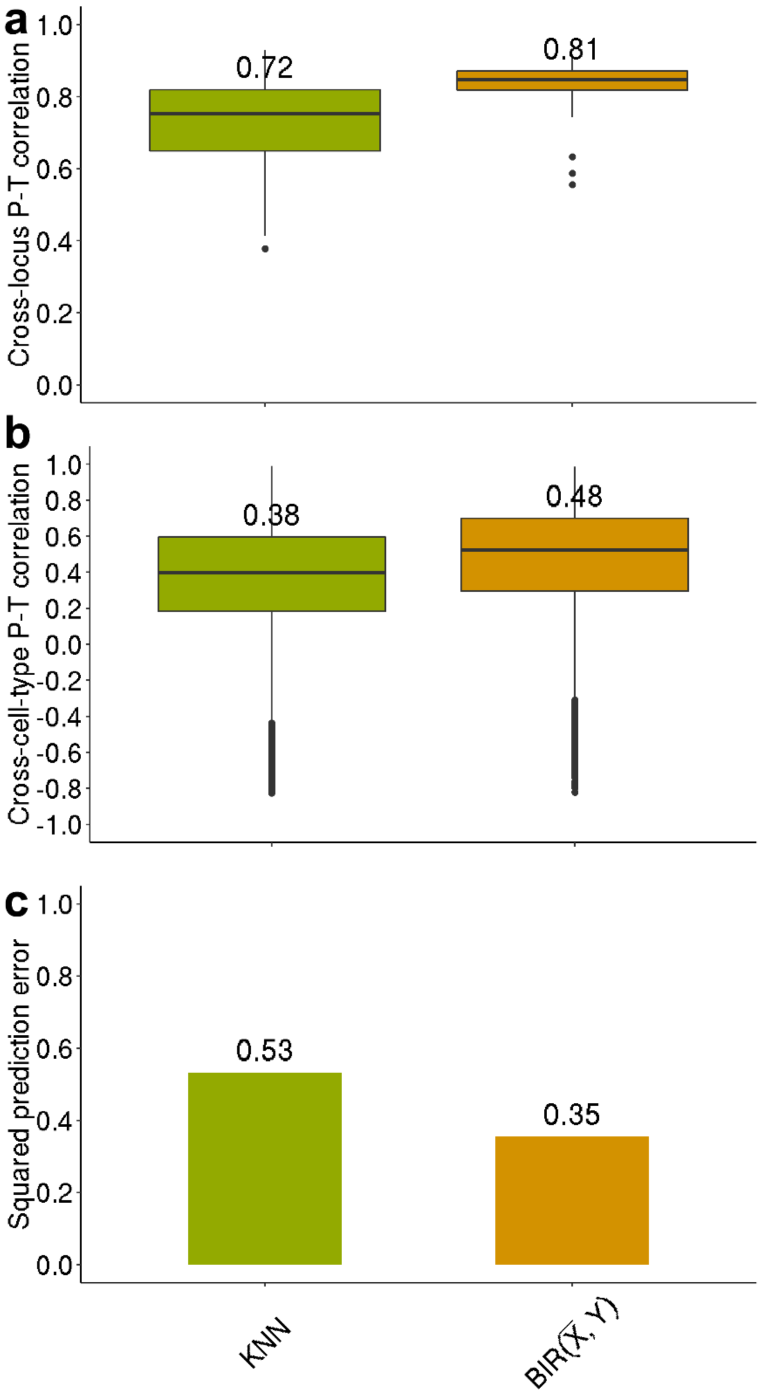

Supplementary Figure 6. Comparison of the whole-genome prediction performance between KNN and $\mathrm{BIR}(\overline{\mathrm{X}}, \mathrm{Y})$. (**a**) Cross-locus P-T correlation r_L. (**b**) Cross-cell-type P-T correlation r_C. (**c**) Squared prediction error τ. In (**a**) and (**b**), each boxplot shows the median (central line), interquartile range (IQR, the 1st (Q1) to 3rd (Q3) quartiles, box), and 1.5 × IQR from the Q1 and Q3 (lower and upper whiskers) of the data. The mean r_L and r_C for each method are shown.

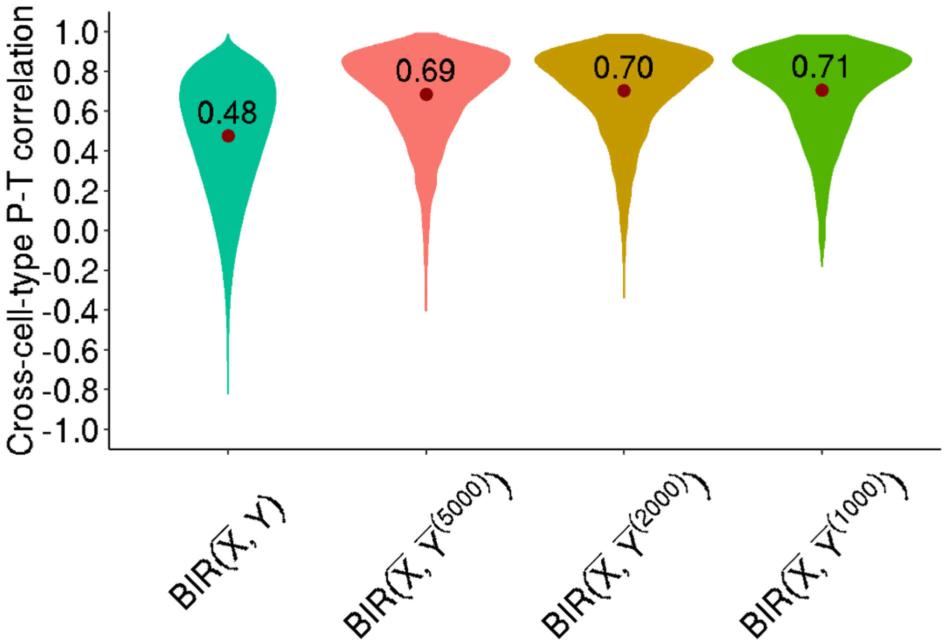

Supplementary Figure 7. Comparison between the locus-level model $\mathrm{BIR}(\overline{\mathrm{X}}, \mathrm{Y})$ and the pathway-level model $\mathrm{BIR}(\overline{\mathrm{X}}, \overline{\mathrm{Y}})$ in terms of cross-cell-type prediction accuracy. For each method, the distribution and mean of cross-cell-type P-T correlation r_C in test cell types are shown. For the pathway-level model, DHSs were clustered into 5000, 2000, and 1000 pathways respectively. $\mathrm{BIR}(\overline{\mathrm{X}}, \overline{\mathrm{Y}})$ had higher r_C compare to $\mathrm{BIR}(\overline{\mathrm{X}}, \mathrm{Y})$, indicating that the pathway-level prediction is more accurate.

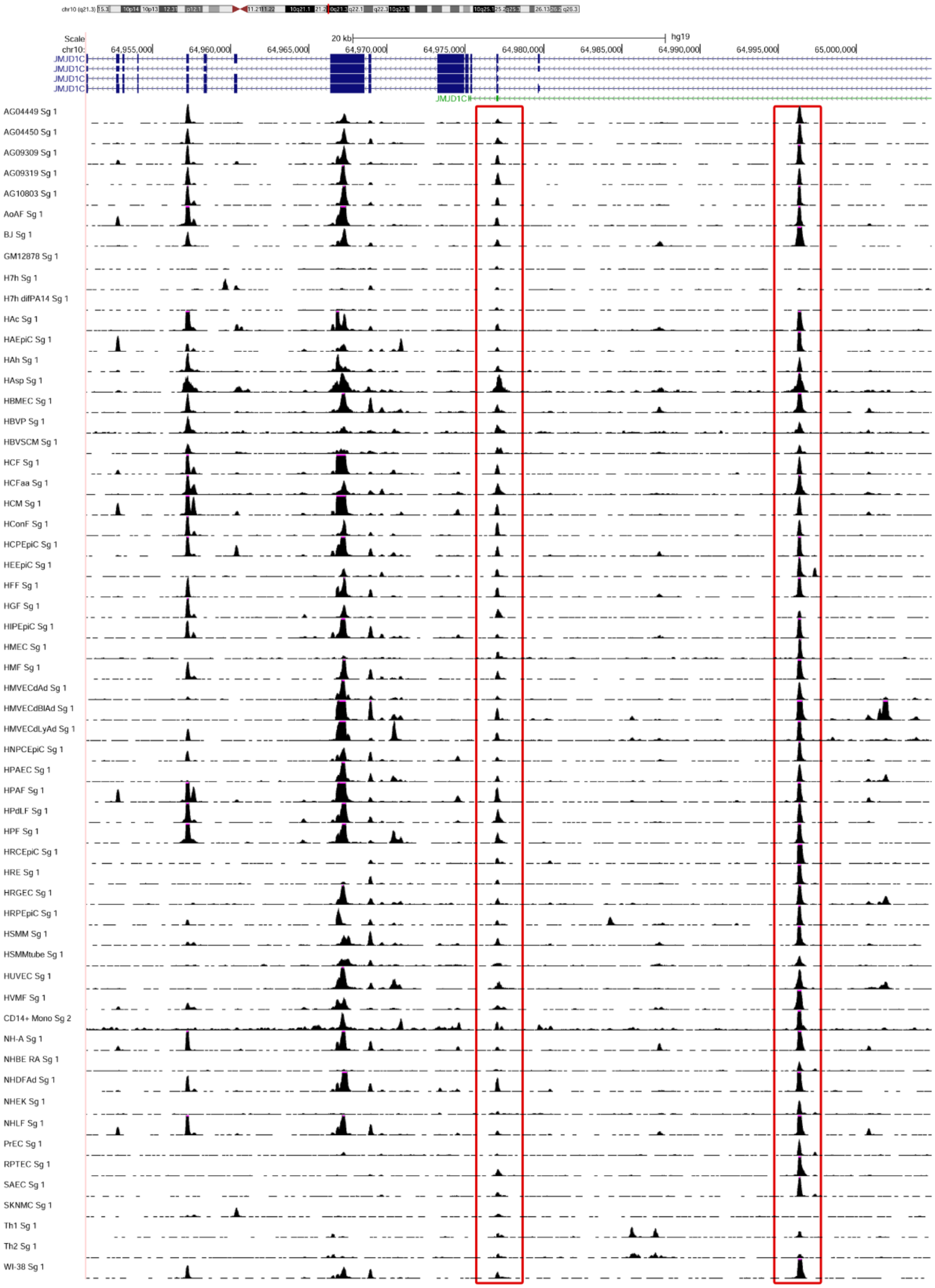

Supplementary Figure 8. DNase-seq signals for 57 cell types in a genomic region to demonstrate the locus effects. The plot shows that DH levels at some loci tend to be consistently higher than the DH levels at other loci in almost all cell types (highlighted regions).

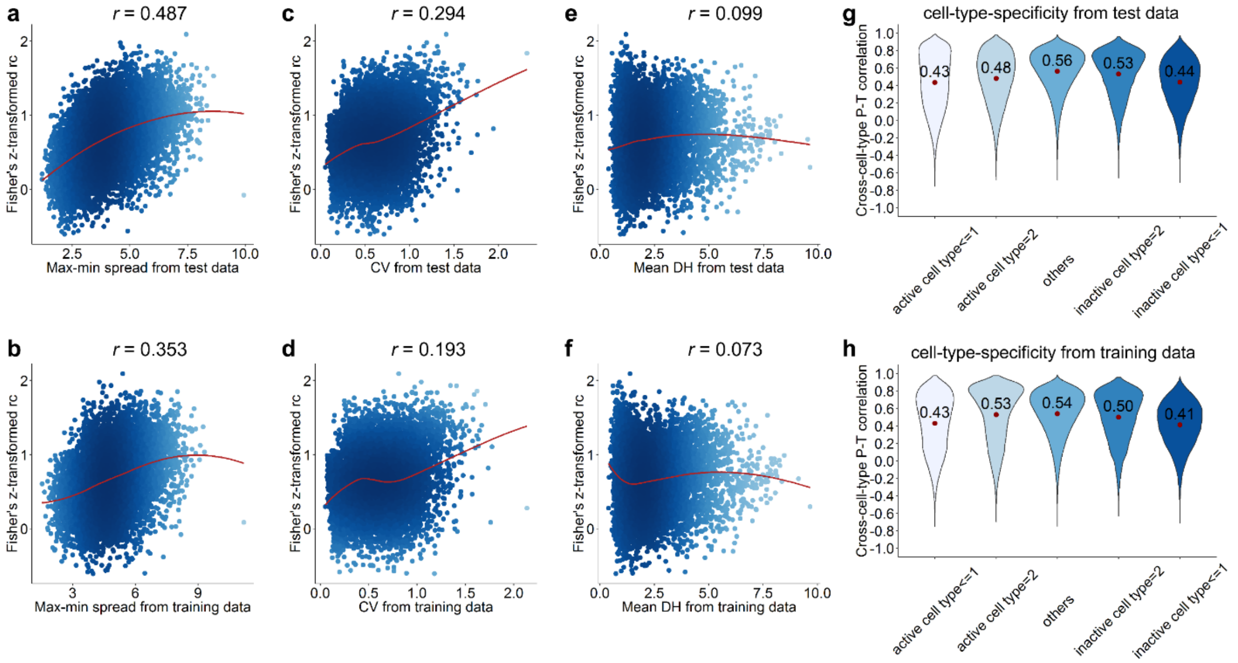

Supplementary Figure 9. Relationship between the cross-cell-type prediction accuracy r_C and different test and training data factors. (**a**)-(**f**) Scatterplots of the Fisher's z-transformed r_C vs. max-min spread (**a**-**b**), coefficient of variation (CV, **c**-**d**), and mean DH (**e**-**f**). For (**a**), (**c**) and (**e**) the max-min spread, CV and mean DH are computed using the true DH values from the test cell types. For (**b**), (**d**) and (**f**), they are computed using the DH values from the training cell types. Each dot in the plot represents a non-noisy locus. The red curves are the loess fit of the data. The Pearson's correlation coefficients are shown on top of the plots. (**g**)-(**h**) Distribution and mean of r_C for loci in different cell-type-specificity categories. The cell-type-specificity is defined using the true DH levels in test data (**g**) and training data (**h**) respectively.

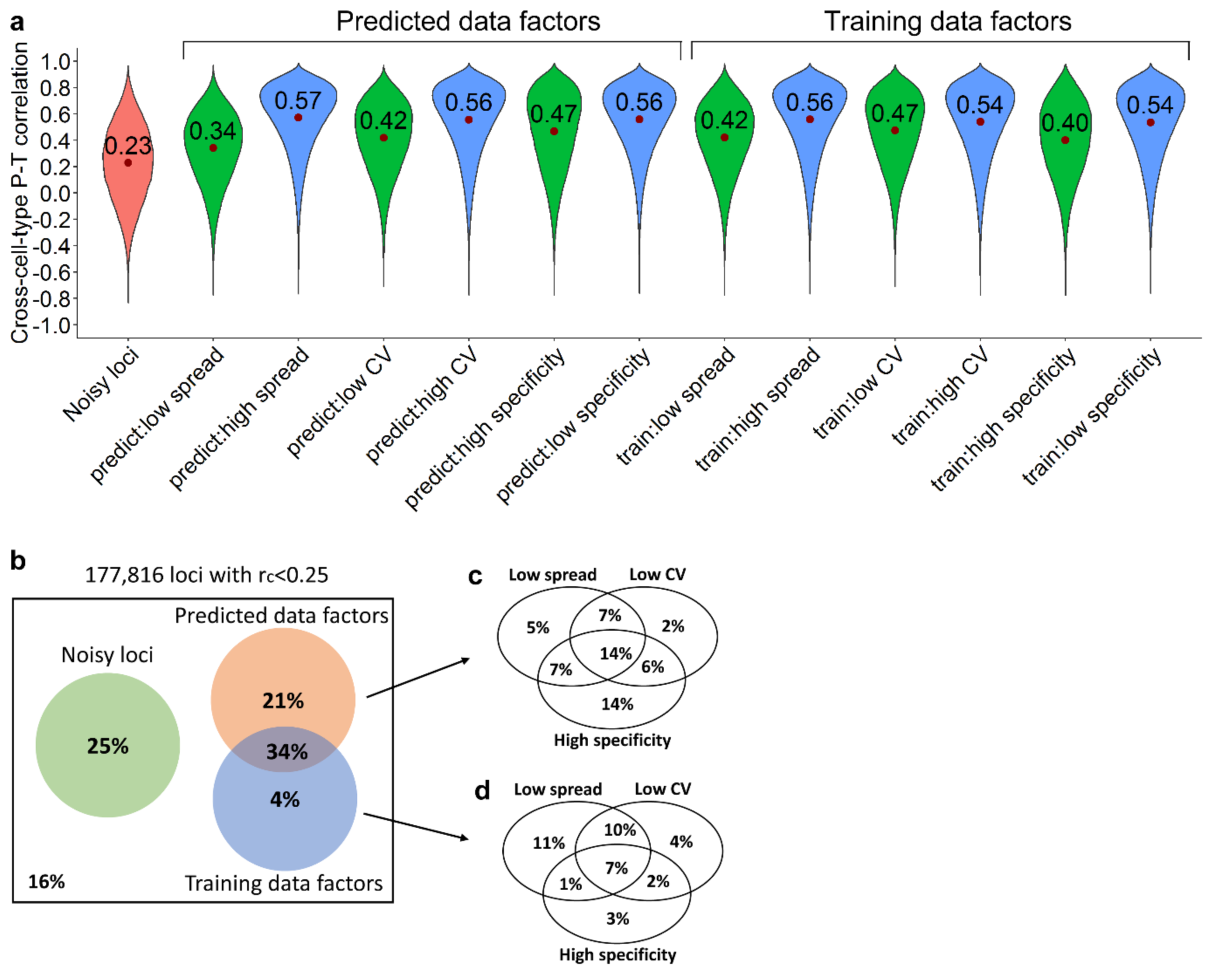

Supplementary Figure 10. Factors affecting a locus' cross-cell-type prediction accuracy (here factors are defined based on the predicted DH values in test cell types and true DH values in training cell types). (**a**) Distribution and mean of r_C for different classes of loci. DHSs were categorized into noisy loci and non-noisy loci in test data using predicted DH values. The non-noisy loci were further divided into two groups based on different factors: low or high max-min spread of DH signals, low or high coefficient of variation (CV), high or low cell-type-specificity. The categorization was done separately using predicted DH values from the test data and true DH values from the training data. (**b**)-(**d**) Percentage of loci with low cross-cell-type prediction accuracy (r_C<0.25) explained by noisy loci, test data factors (low max-min spread, low CV, high cell-type-specificity in test cell types; details shown in (**c**)) and training data factors (low max-min spread, low CV, high cell-type-specificity in training cell types; details shown in (**d**)).

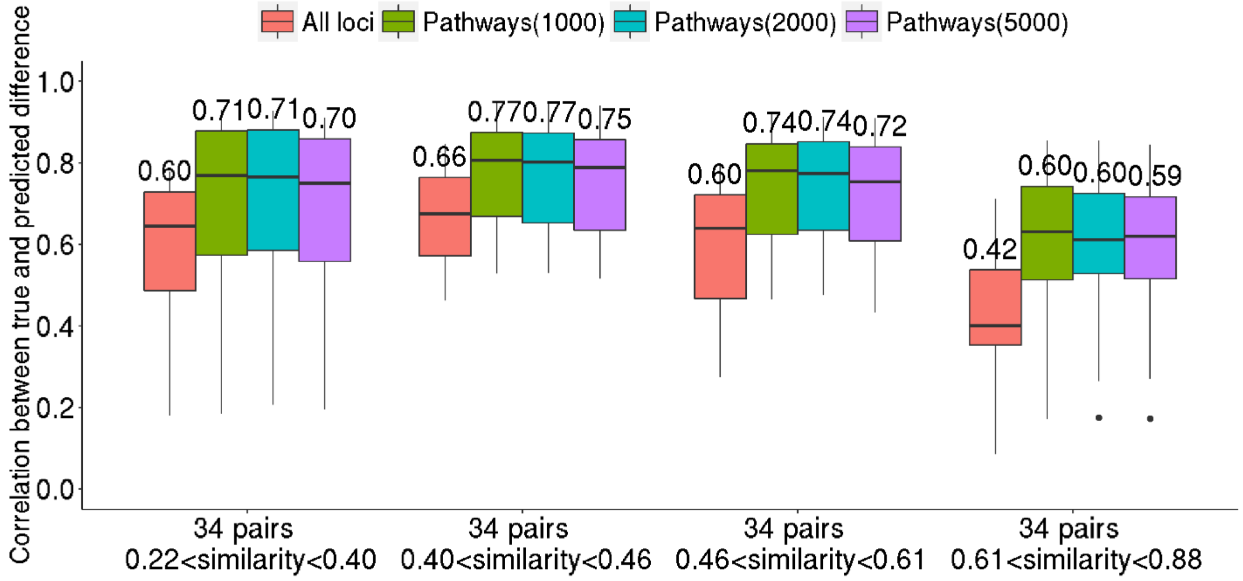

Supplementary Figure 11. Comparison of prediction performance in predicting differential DH in all loci by BIRD and in 1000, 2000 and 5000 DHS pathways by the pathway-level model $\mathrm{BIR}(\overline{\mathrm{X}}, \overline{\mathrm{Y}})$. All pairs of test cell types were stratified based on the quartiles of the similarity between the two compared cell types. For each stratum, the distribution and mean of the prediction-truth Pearson's correlation across all loci or pathways are shown for each method. Each boxplot shows the median (central line), interquartile range (IQR, the 1st (Q1) to 3rd (Q3) quartiles, box), and 1.5 × IQR from the Q1 and Q3 (lower and upper whiskers) of the data.

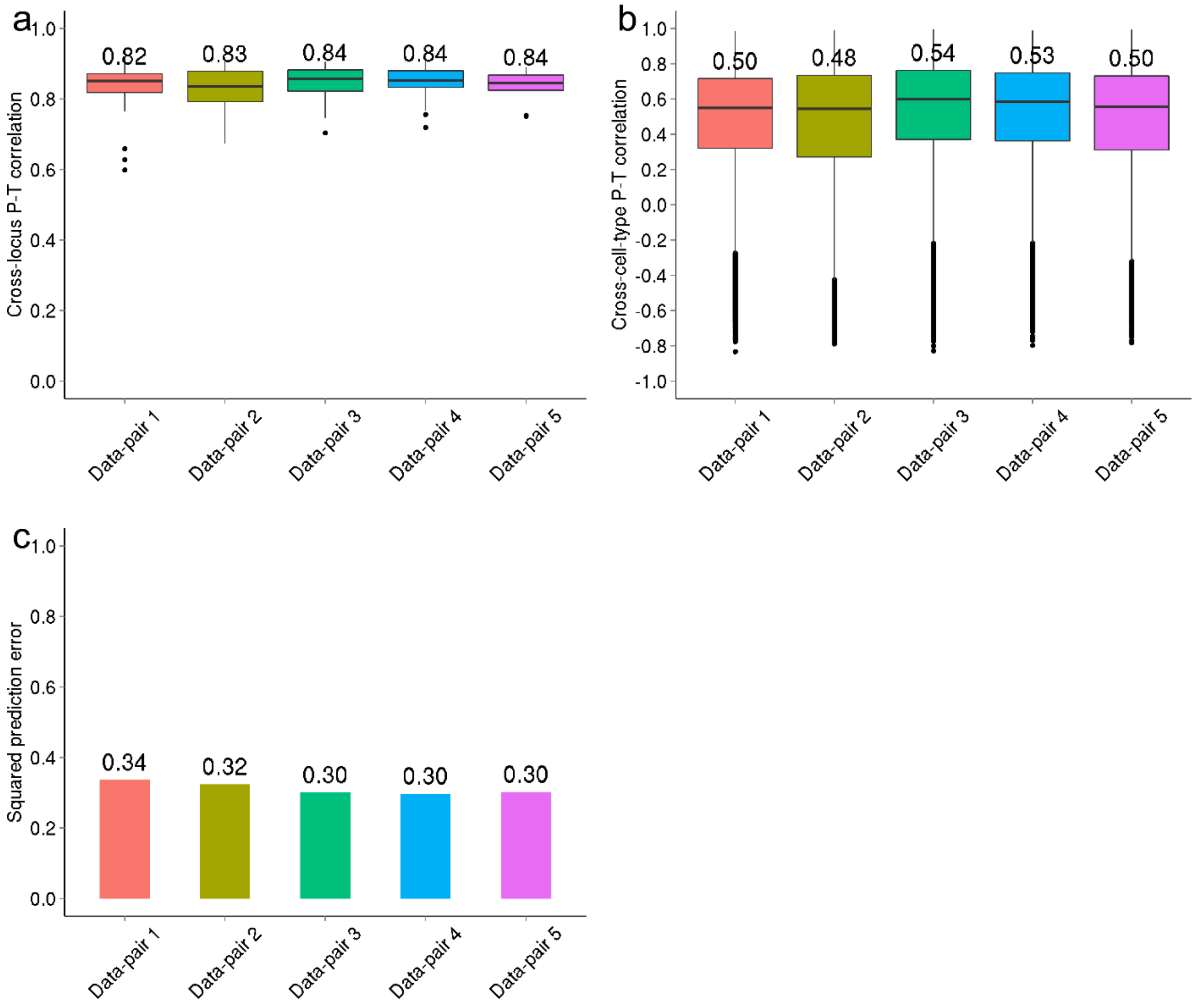

Supplementary Figure 12. Prediction performance of BIRD for five different training-test data partitions in terms of (**a**) cross-locus P-T correlation r_L, (**b**) cross-cell-type P-T correlation r_C, and (**c**) squared prediction error τ. In (**a**) and (**b**), each boxplot shows the median (central line), interquartile range (IQR, the 1st (Q1) to 3rd (Q3) quartiles, box), and 1.5 × IQR from the Q1 and Q3 (lower and upper whiskers) of the data. The mean r_L and r_C for each partition are shown.

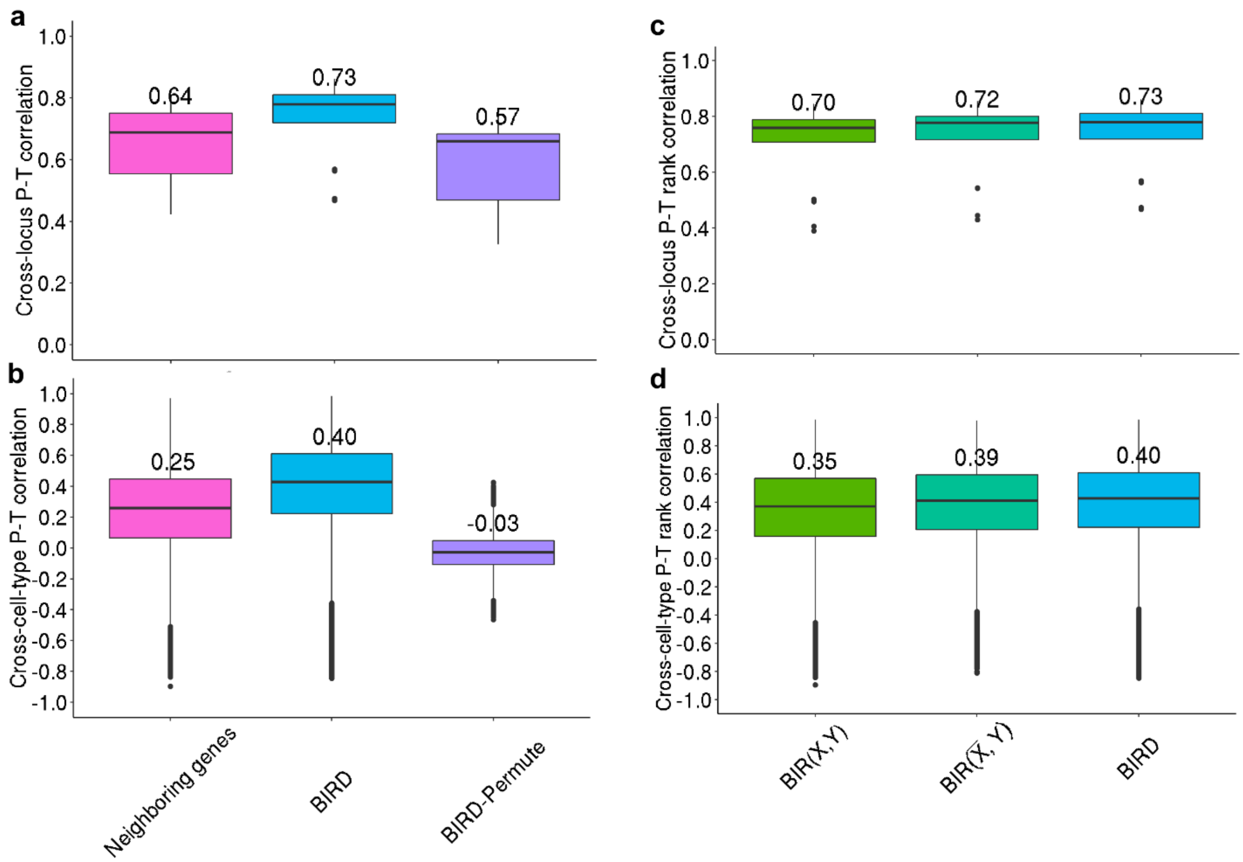

Supplementary Figure 13. Comparison of different methods using Spearman's rank correlation instead of Pearson's correlation.

(**a**)-(**b**) Comparison among the neighboring gene approach, BIRD, and the random model. (**a**) Cross-locus P-T correlation r_L based on Spearman's rank correlation. (**b**) Cross-cell-type P-T correlation r_C based on Spearman's rank correlation.

(**c**)-(**d**) Comparison of $\mathrm{BIR}(\mathrm{X},\mathrm{Y})$ (no clustering of predictors), $\mathrm{BIR}(\overline{\mathrm{X}},\mathrm{Y})$ (with predictor clustering), and BIRD. (**c**) Cross-locus P-T correlation r_L based on Spearman's rank correlation. (**d**) Cross-cell-type P-T correlation r_C based on Spearman's rank correlation.

(**a**)-(**b**) are analogous to **Fig. 2b-d** which were based on Pearson's correlation. (**c**)-(**d**) are analogous to **Supplementary Fig. 4a-b.**

Each boxplot shows the median (central line), interquartile range (IQR, the 1st (Q1) to 3rd (Q3) quartiles, box), and 1.5 × IQR from the Q1 and Q3 (lower and upper whiskers) of the data. The mean r_L and r_C for each method are shown.

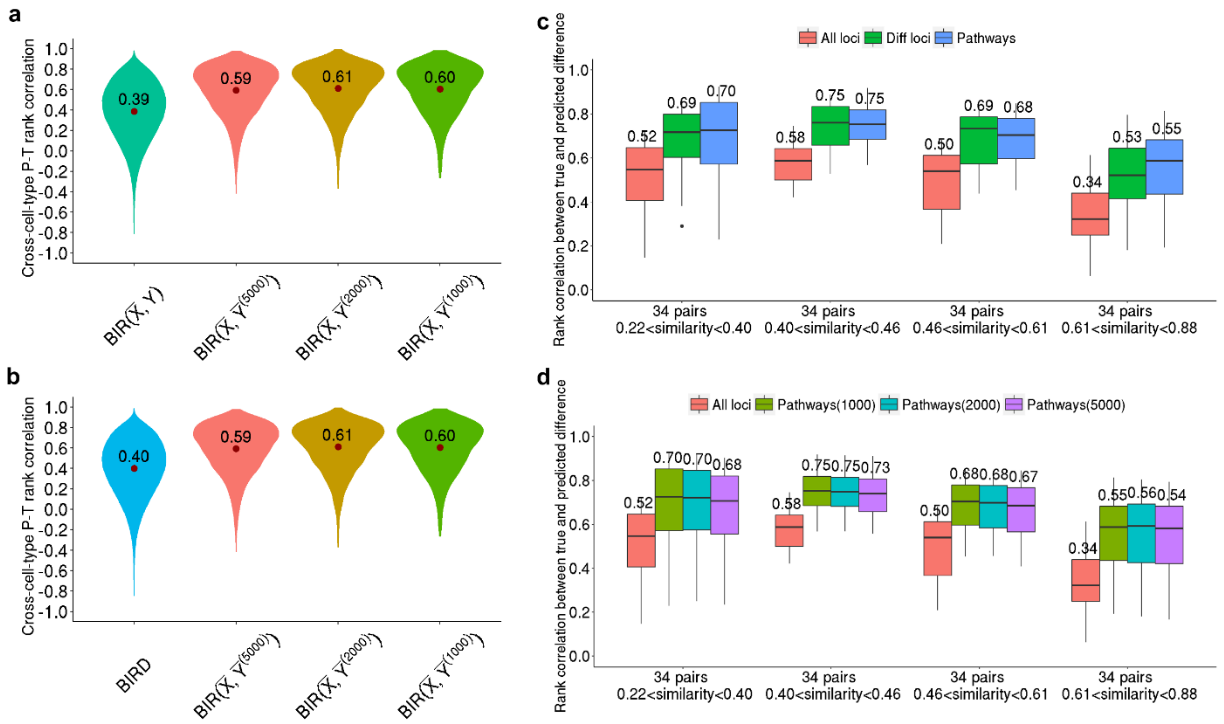

Supplementary Figure 14. Comparison of different methods using Spearman's rank correlation instead of Pearson's correlation (cont'd). (**a**) Comparison between the locus-level model $\mathrm{BIR}(\overline{\mathrm{X}}, \mathrm{Y})$ and pathway-level model $\mathrm{BIR}(\overline{\mathrm{X}}, \overline{\mathrm{Y}})$ in terms of cross-cell-type P-T correlation r_C where r_C is computed using Spearman's rank correlation. (**b**) Comparison between BIRD and $\mathrm{BIR}(\overline{\mathrm{X}}, \overline{\mathrm{Y}})$ in terms of cross-cell-type P-T correlation r_C computed using Spearman's rank correlation. (**c**) Comparison of prediction performance in predicting differential DH in all loci, differential loci, and 1000 pathways based on the prediction-truth correlation computed using Spearman's rank correlation. (**d**) Comparison of prediction performance in predicting differential DH in all loci, 1000 pathways, 2000 pathways, and 5000 pathways based on Spearman's rank correlation. The mean for each method is shown. In (**c**) and (**d**), each boxplot shows the median (central line), interquartile range (IQR, the 1st (Q1) to 3rd (Q3) quartiles, box), and 1.5 × IQR from the Q1 and Q3 (lower and upper whiskers) of the data.

(**a**) is analogous to **Supplementary Fig. 7** which was based on Pearson's correlation. (**b**) is analogous to **Fig. 3f.** (**c**) is analogous to **Fig. 3g.** (**d**) is analogous to **Supplementary Fig. 11.**

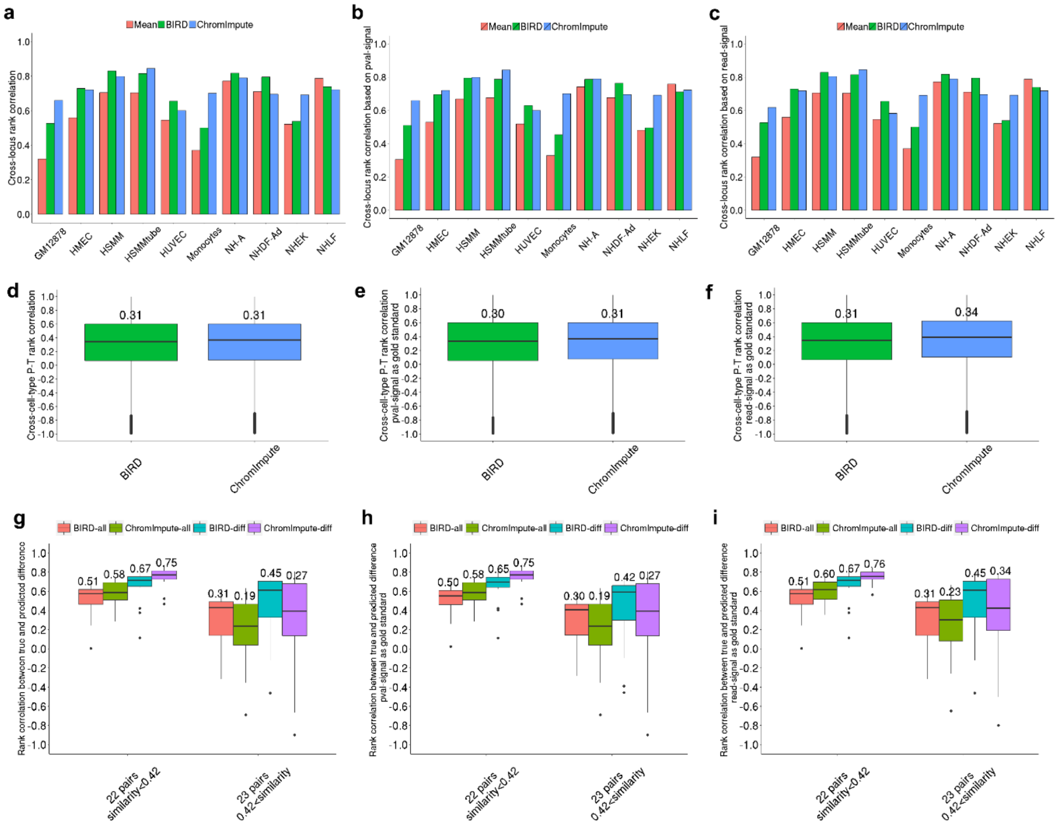

Supplementary Figure 15. Comparison between BIRD and ChromImpute. (**a**) Cross-locus Spearman's rank correlation between BIRD- or Mean- predicted read-signal and true read-signal, and between ChromImpute-predicted pval-signal and true MACS pval-signal. (**b**) Cross-locus Spearman's rank correlation between BIRD- or Mean- predicted read-signal and true MACS pval-signal, and between ChromImpute-predicted pval-signal and true MACS pval-signal. (**c**) Cross-locus Spearman's rank correlation between BIRD- or Mean- predicted read-signal and true read signal, and between ChromImpute-predicted pval-signal and true read-signal. (**d**) Cross-cell-type Spearman's rank correlation r_C between the true read-signal and BIRD-predicted read-signal, and between the true pval-signal and ChromImpute-predicted pval-signal. (**e**) Cross-cell-type Spearman's rank correlation r_C between the true pval-signal and BIRD-predicted read-signal or ChromImpute-predicted pval-signal. (**f**) Cross-cell-type Spearman's rank correlation r_C between the true read-signal and BIRD-predicted read-signal or ChromImpute-predicted pval-signal. (**g**)-(**i**) Spearman's rank correlation between the true and predicted differential DH signal. The true signal is based on the true read-signal for BIRD and true pval-signal for ChromImpute (**g**), true pval-signal (**h**) and true read-signal (**i**) respectively. The 45 test cell type pairs were divided into two groups based on the median of similarity between the two compared cell types. For each group, the distribution and mean of the prediction-truth Spearman's rank correlation across all loci and differential loci are shown. In (**d**)-(**i**), each boxplot shows the median (central line), interquartile range (IQR, the 1st (Q1) to 3rd (Q3) quartiles, box), and 1.5 × IQR from the Q1 and Q3 (lower and upper whiskers) of the data. The mean for each method is shown.

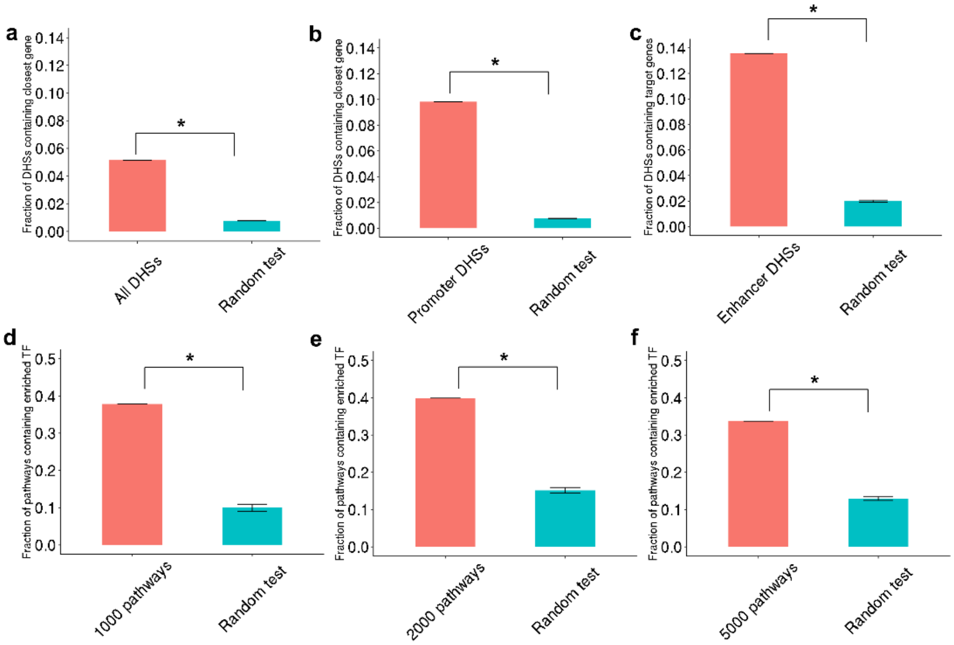

g

Active cell types

cell_type	enrichment	FDR	active
**HSMM**	2.553008	2.77E-192	1
**HSMMtube**	1.412009	6.01E-166	1

h

Top enriched motifs

motif	num of motif	enrichment	FDR
MA0832.1:Tcf21	90	12.565	1.52E-34
MA0691.1:TFAP4	174	6.789	6.71E-49
MA0667.1:**MYF6**	141	6.153	3.39E-37
MA0816.1:Ascl2	246	5.164	9.76E-57
MA0521.1:Tcf12	419	5.029	3.50E-94
MA0091.1:TAL1::TCF3	167	5.015	1.01E-37
MA0665.1:MSC	170	4.989	3.97E-38
MA0499.1:**Myod1**	402	4.903	5.17E-89
MA0500.1:**Myog**	416	4.786	6.85E-90
M00927_AP4_Q6_01	367	3.842	3.88E-64
...	...	...	...

i

Predictor genes

...
MYF6
MYH1
MYH13
MYH3
MYH4
MYH8
MYHAS
MYL1
MYL4
MYLPF
MYO18B
MYOD1
MYOG
...

j

GO analysis of predictor genes

GO ID	GO Term	P-value	FDR
GO:0003008	system process	2.90E-23	4.24E-19
GO:0030049	muscle filament sliding	1.20E-21	5.85E-18
GO:0033275	actin-myosin filament sliding	1.20E-21	5.85E-18
GO:0006941	striated muscle contraction	3.70E-20	1.35E-16
GO:0007517	muscle organ development	7.20E-20	2.11E-16
GO:0061061	muscle structure development	6.20E-19	1.51E-15
GO:0070252	actin-mediated cell contraction	1.60E-17	3.34E-14
GO:0014706	striated muscle tissue development	8.20E-17	1.50E-13
GO:0060537	muscle tissue development	1.90E-16	3.09E-13
GO:0030048	actin filament-based movement	2.70E-16	3.95E-13
GO:0055001	**muscle cell development**	2.40E-14	3.19E-11
GO:0060047	heart contraction	3.00E-14	3.66E-11
GO:0003015	heart process	3.50E-14	3.94E-11
GO:0007519	**skeletal muscle tissue development**	6.00E-14	6.27E-11
GO:0055002	striated muscle cell development	1.30E-13	1.27E-10
GO:0060538	skeletal muscle organ development	1.80E-13	1.64E-10
GO:0003009	skeletal muscle contraction	2.50E-12	2.15E-09
GO:0008015	blood circulation	2.80E-12	2.27E-09
GO:0003013	circulatory system process	3.00E-12	2.31E-09
GO:0044057	regulation of system process	3.80E-12	2.78E-09
...	...	...	...

Supplementary Figure 16. Analysis of predictors selected by BIRD.

(**a**) Fraction of DHSs that contain their closest genes as predictors (total number of all DHSs: *n*=1,108,603). (**b**) Fraction of promoter DHSs that contain their closest genes as predictors (total number of promoter DHSs: *n*=88,887). (**c**) Fraction of enhancer DHSs that contain their target genes as predictors (total number of enhancer DHSs: *n*=28,563). (**d**)-(**f**) Fraction of pathways that contain their enriched TFs as predictors. DHSs are clustered into 1000 pathways (**d**, *n*=1000), 2000 pathways (**e**, *n*=2000), and 5000 pathways (**f**, *n*=5000) respectively. In (**a**)-(**f**), "Random test" shows the expected fraction by chance (**Supplementary Note 2**, the sample size *n* in the Random test is the same as the group being compared). * Permutation test *p*-values < 0.001 (1000 permutations per test). Data are presented as mean ± s.d.

(**g**)-(**j**) Pathway-level prediction model for an example DHS pathway. (**g**) Active cell types of the pathway. (**h**) Top-ranked enriched motifs in the DHSs of the pathway. (**i**) Predictor genes for the pathway. (**j**) Top-ranked GO terms enriched in the predictor genes of the pathway.

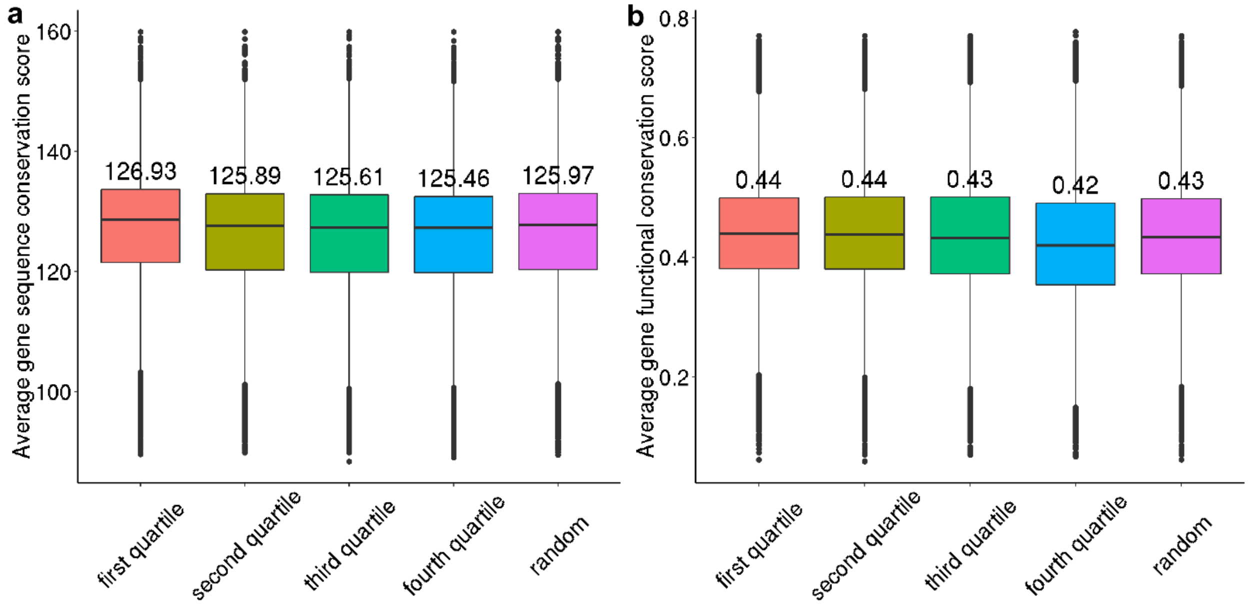

Supplementary Figure 17. Relationship between sequence conservation of DHSs and sequence or functional conservation of their predictor genes. (**a**) DHSs are stratified by the quartiles of their sequence conservation (on the x-axis) and the boxplots represent the sequence conservation of the predictor genes in each group of DHSs. The mean for each group is shown. (**b**) The boxplots represent the functional conservation of the predictor genes in each group of DHSs (see **Supplementary Note 3**). Each boxplot shows the median (central line), interquartile range (IQR, the 1st (Q1) to 3rd (Q3) quartiles, box), and 1.5 × IQR from the Q1 and Q3 (lower and upper whiskers) of the data. The mean for each group is shown.

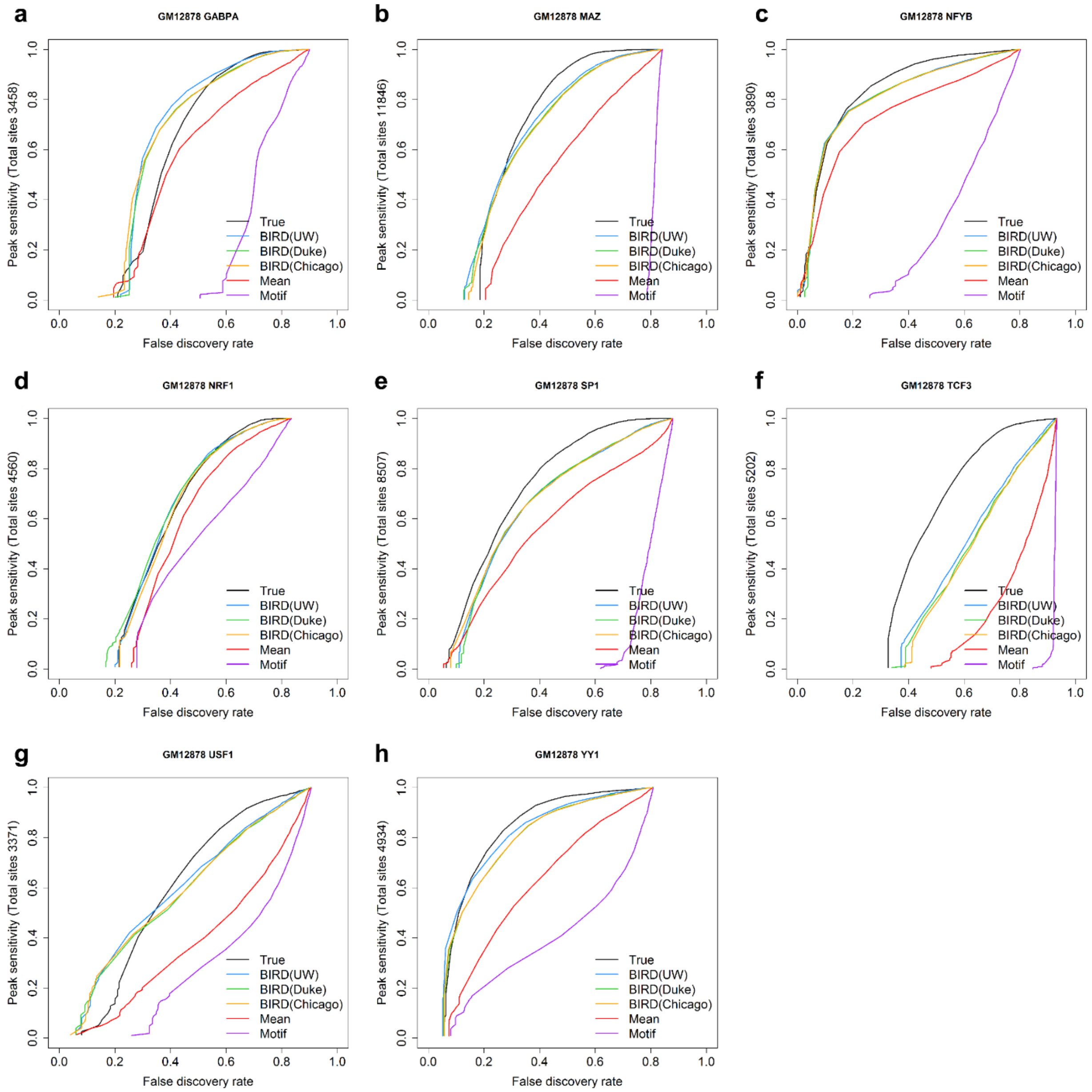

Supplementary Figure 18. Sensitivity-FDR curves for predicting transcription factor binding sites of GABPA, MAZ, NFYB, NRF1, SP1, TCF3, USF1, and YY1 in GM12878 using four different methods: true DNase-seq data ("True"), BIRD, mean DH profile of training cell types ("Mean"), and the motif mapping score ("Motif"). For BIRD, "BIRD(UW)", "BIRD(Duke)" and "BIRD(Chicago)" denote predictions made using exon arrays generated by three different labs.

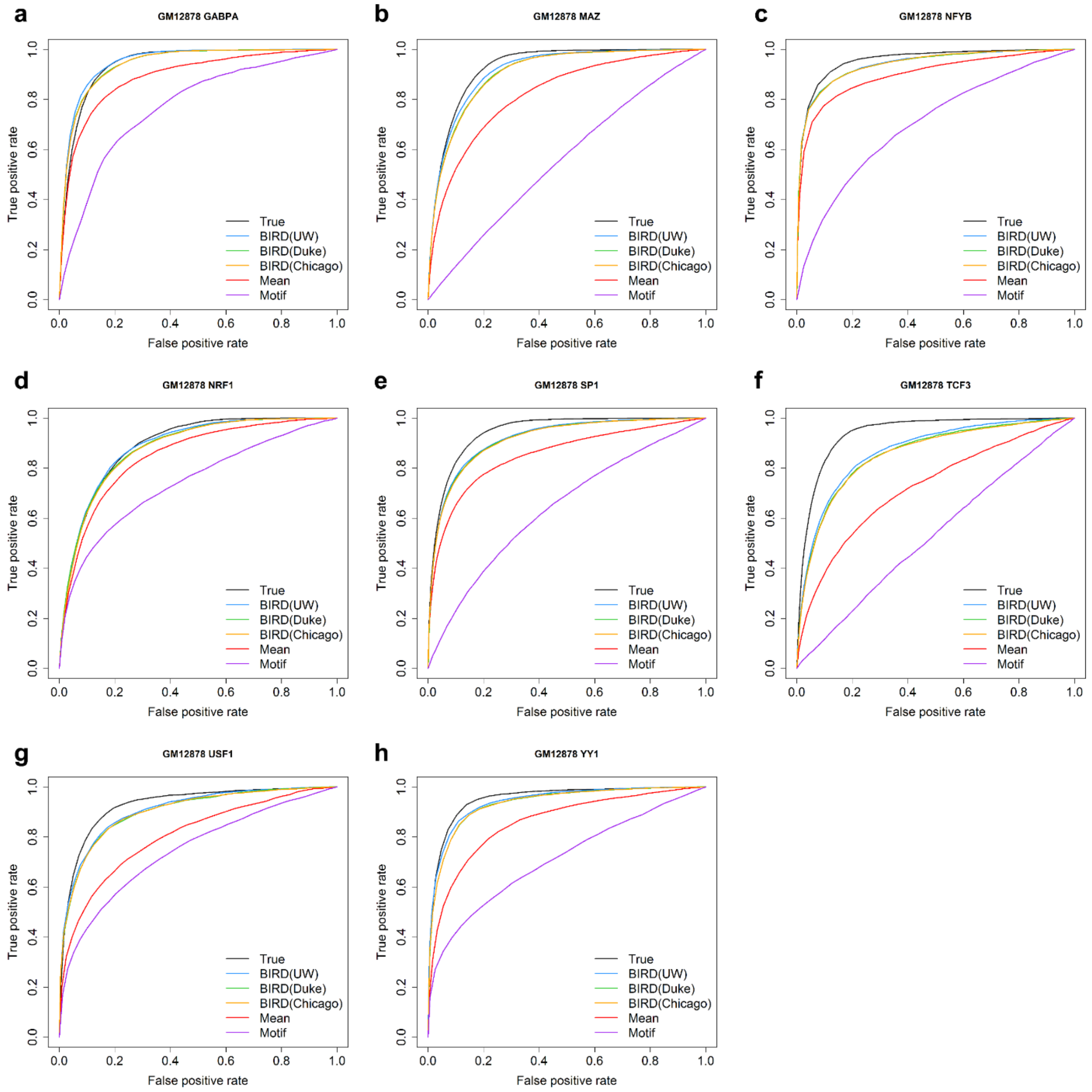

Supplementary Figure 19. ROC curves for predicting transcription factor binding sites of GABPA, MAZ, NFYB, NRF1, SP1, TCF3, USF1, and YY1 in GM12878 using four different methods: true DNase-seq data ("True"), BIRD, mean DH profile of training cell types ("Mean"), and the motif mapping score ("Motif"). For BIRD, "BIRD(UW)", "BIRD(Duke)" and "BIRD(Chicago)" denote predictions made using exon arrays generated by three different labs.

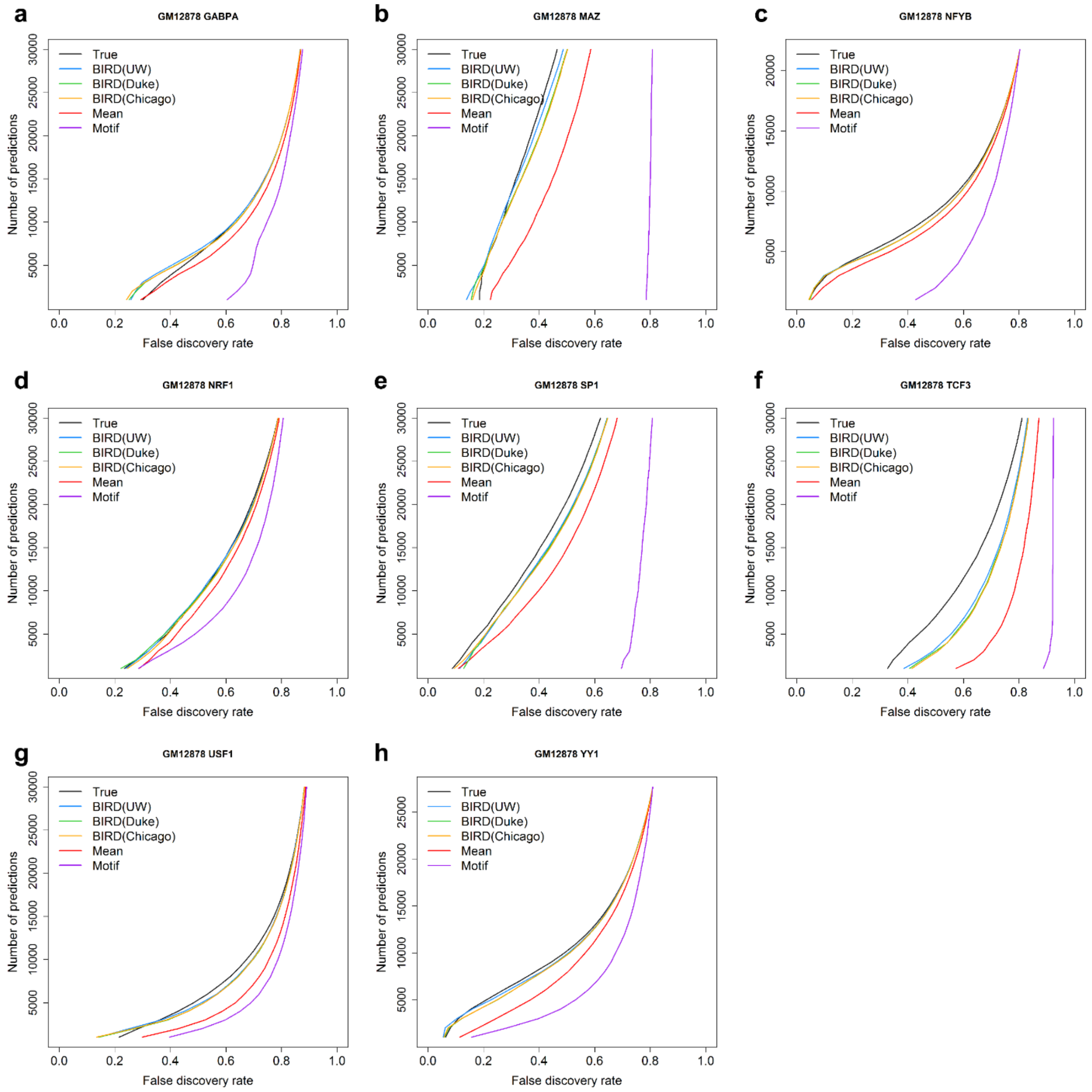

Supplementary Figure 20. The number of DHSs predicted to be binding sites of GABPA, MAZ, NFYB, NRF1, SP1, TCF3, USF1, and YY1 in GM12878 at different FDR levels using four different methods: true DNase-seq data ("True"), BIRD, mean DH profile of training cell types ("Mean"), and the motif mapping score ("Motif"). For BIRD, "BIRD(UW)", "BIRD(Duke)" and "BIRD(Chicago)" denote predictions made using exon arrays generated by three different labs.

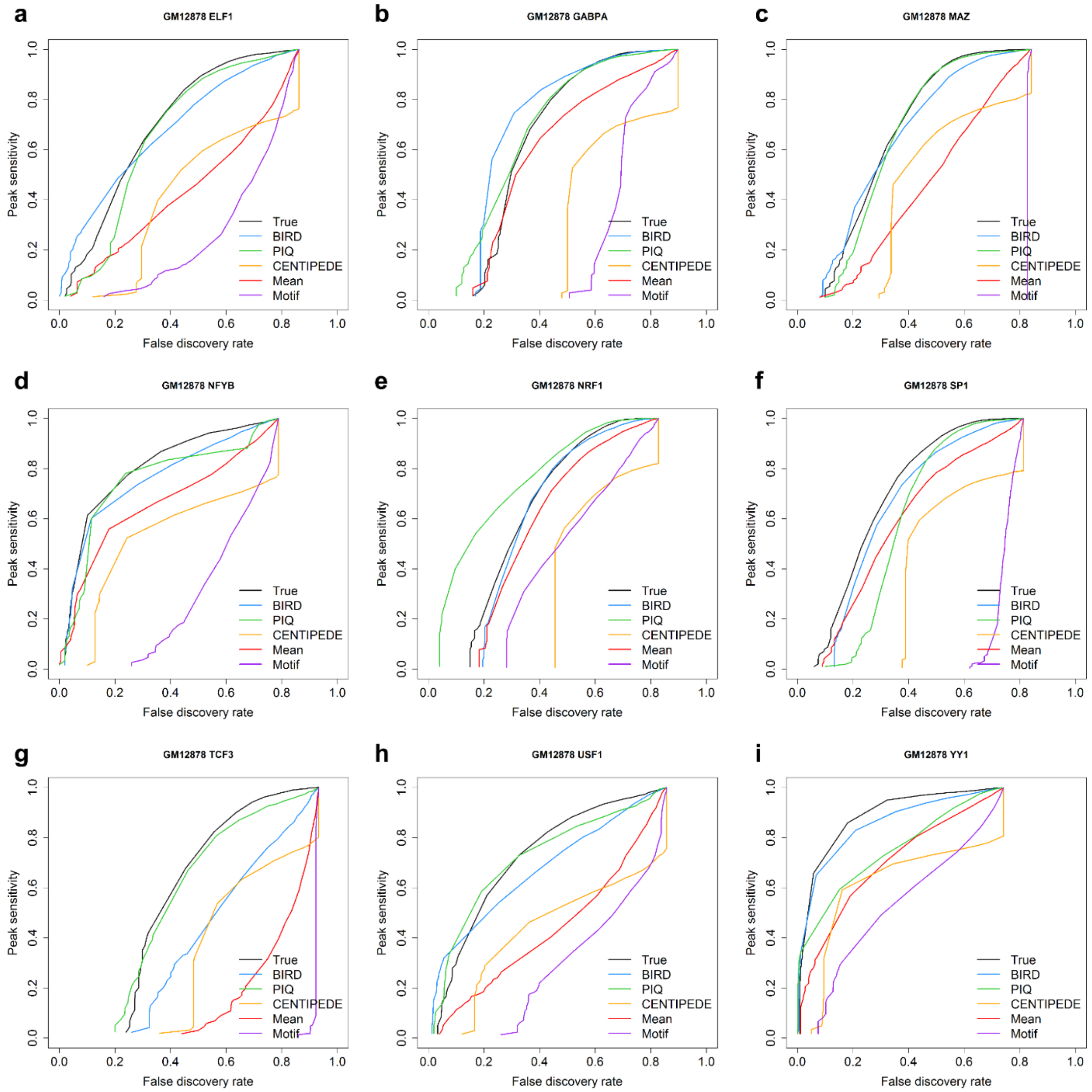

Supplementary Figure 21. Sensitivity-FDR curves for comparing BIRD (based on gene expression from UW), PIQ, and CENTIPEDE for predicting binding sites of ELF1, GABPA, MAZ, NFYB, NRF1, SP1, TCF3, USF1, and YY1 in GM12878. Prediction using the true DNase-seq data (“True”), mean DH profile of training cell types (“Mean”), and the motif mapping score (“Motif”) are also shown.

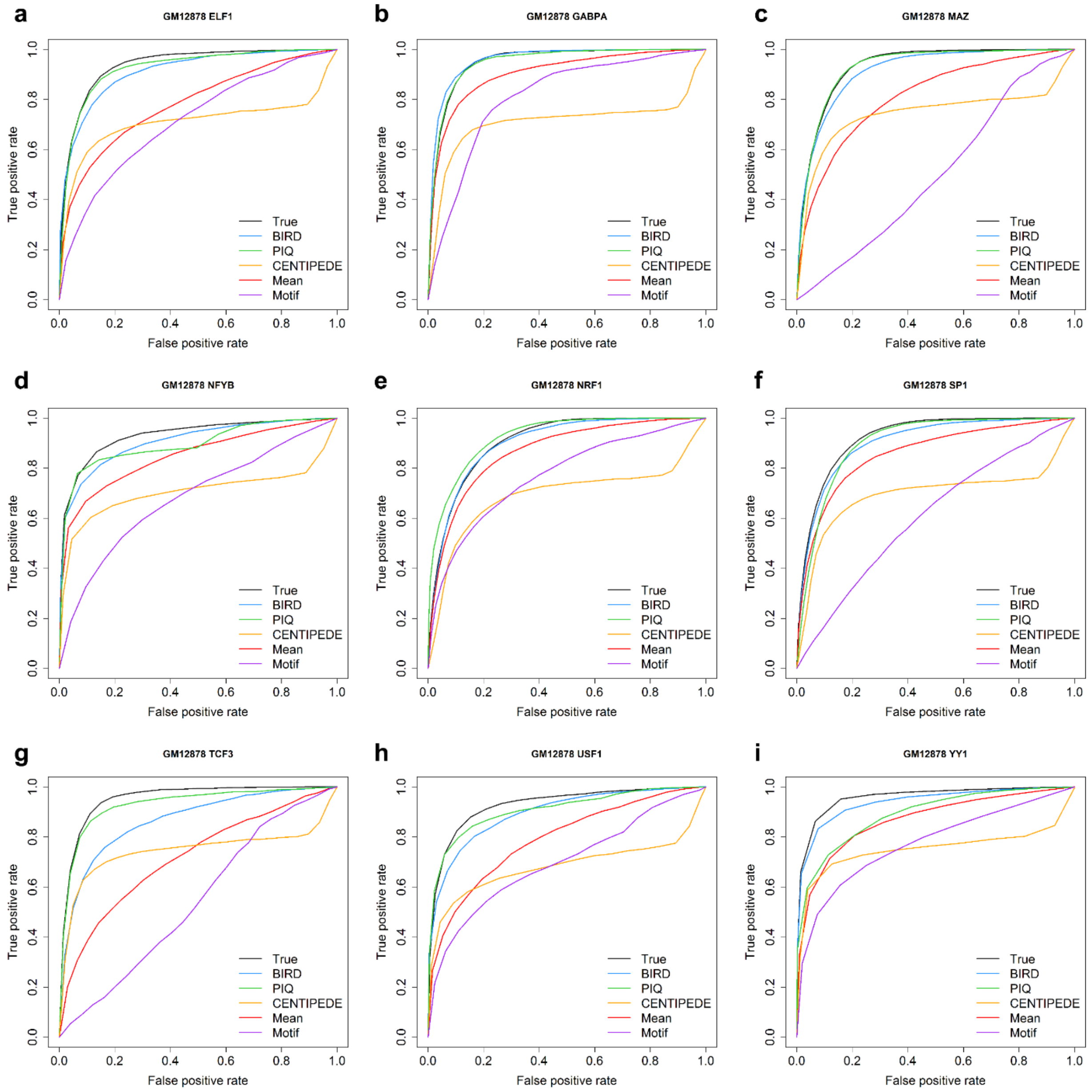

Supplementary Figure 22. ROC curves for comparing BIRD (based on gene expression from UW), PIQ, and CENTIPEDE for predicting binding sites of ELF1, GABPA, MAZ, NFYB, NRF1, SP1, TCF3, USF1, and YY1 in GM12878. Prediction using the true DNase-seq data ("True"), mean DH profile of training cell types ("Mean"), and the motif mapping score ("Motif") are also shown.

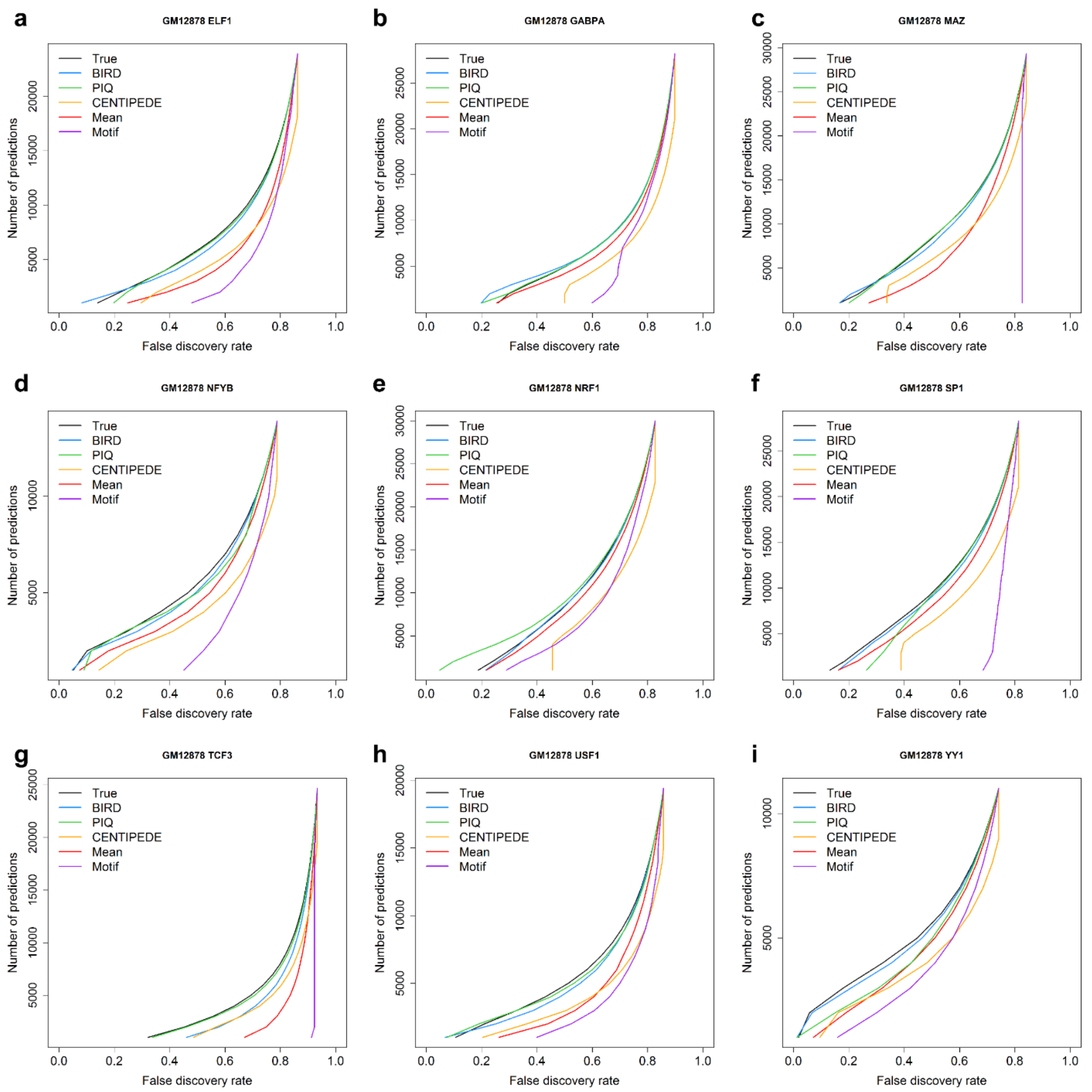

Supplementary Figure 23. The number of DHSs predicted to be TFBSs by BIRD (based on gene expression from UW), PIQ, and CENTIPEDE at different FDR levels for ELF1, GABPA, MAZ, NFYB, NRF1, SP1, TCF3, USF1, and YY1 in GM12878. Prediction using the true DNase-seq data ("True"), mean DH profile of training cell types ("Mean"), and the motif mapping score ("Motif") are also shown.

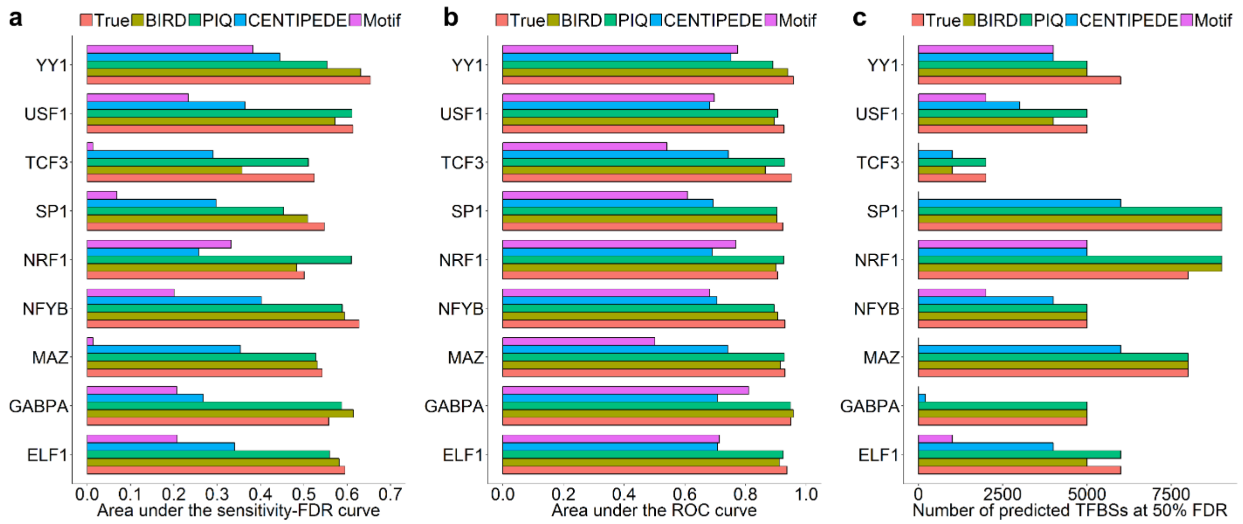

Supplementary Figure 24. Comparison of BIRD, PIQ, and CENTIPEDE in terms of (**a**) area under the sensitivity-FDR curve, (**b**) area under the ROC curve, and (**c**) number of predicted TFBSs at 50% FDR for predicting 9 TFs in GM12878. Results from prediction using the true DNase-seq data ("True") and the motif mapping score ("Motif") are also shown.

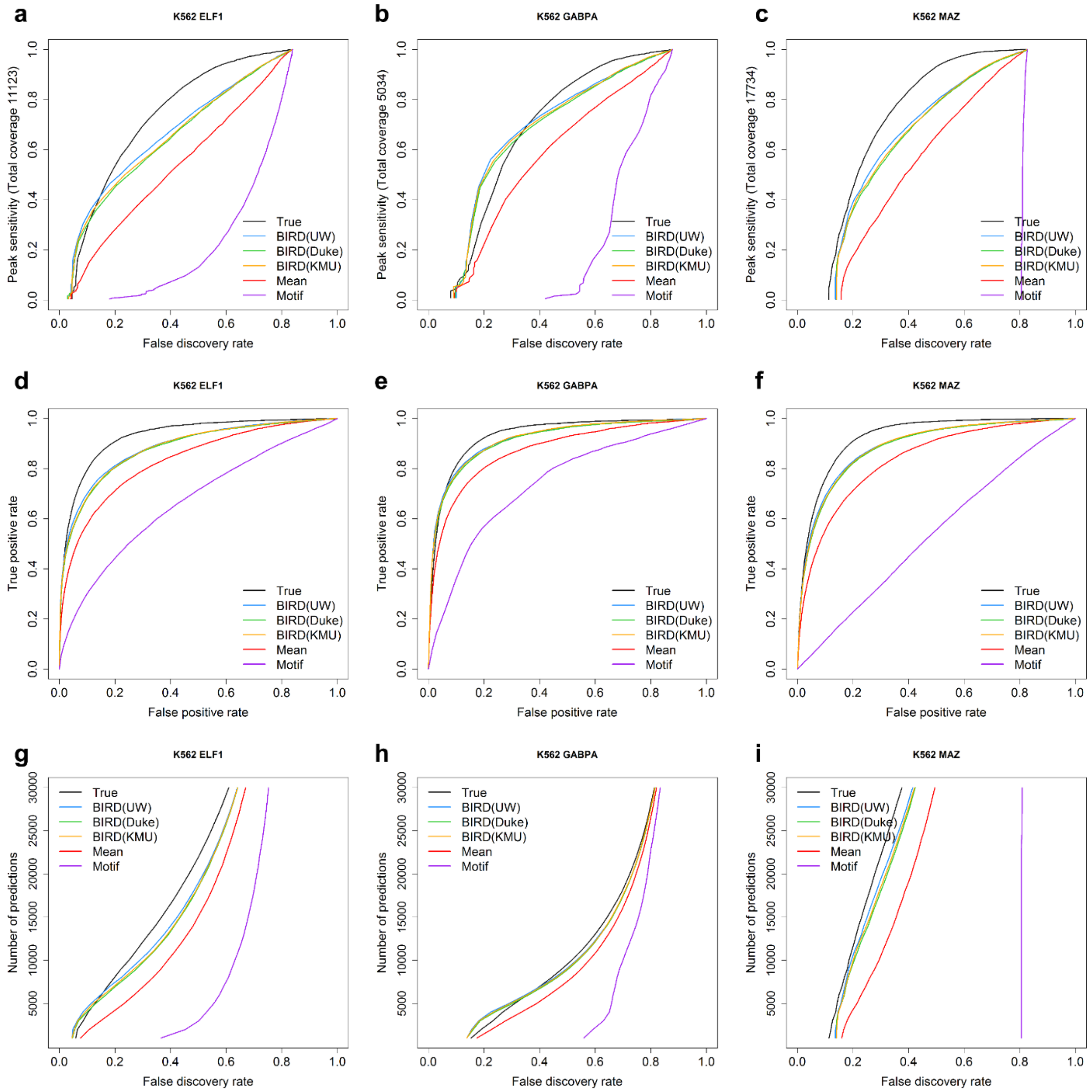

Supplementary Figure 25. Predicting transcription factor binding sites of ELF1, GABPA, and MAZ in K562 using four different methods: true DNase-seq data ("True"), BIRD, mean DH profile of training cell types ("Mean"), and the motif mapping score ("Motif"). For BIRD, "BIRD(UW)", "BIRD(Duke)" and "BIRD(KMU)" denote predictions made using exon arrays generated by three different labs. (**a**)-(**c**) Sensitivity-FDR curves. (**d**)-(**f**) ROC curves. (**g**)-(**i**) The number of DHSs predicted to be TFBSs at different FDR levels.

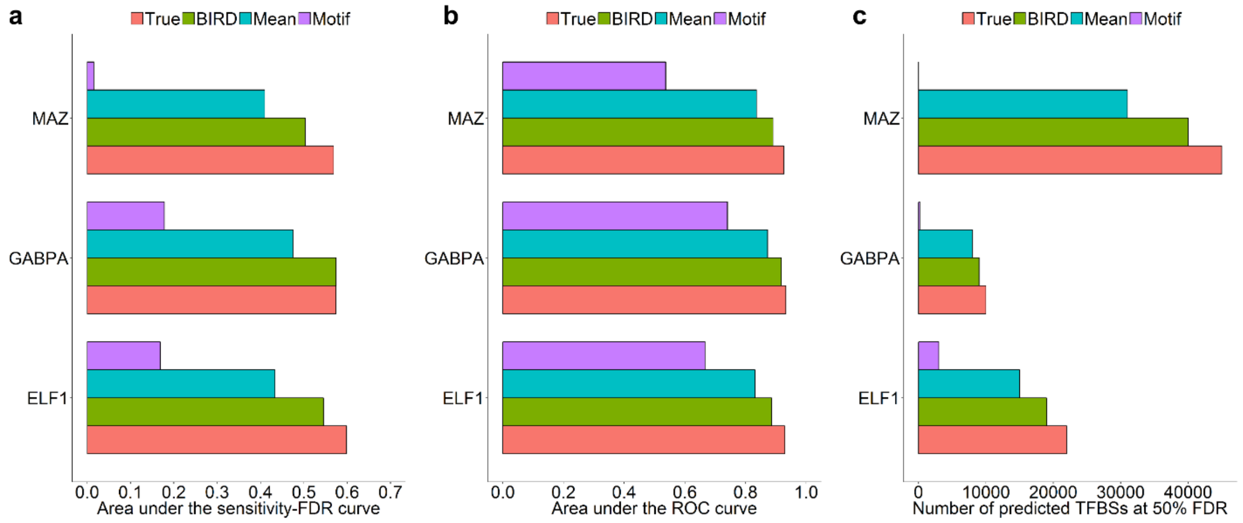

Supplementary Figure 26. Prediction performance for predicting 3 TFs in K562 of four different methods: true DNase-seq data ("True"), BIRD (based on gene expression from UW), mean DH profile of training cell types ("Mean"), and the motif mapping score ("Motif"). (**a**) Area under the sensitivity-FDR curve. (**b**) Area under the ROC curve. (**c**) Number of predicted TFBSs at 50% FDR.

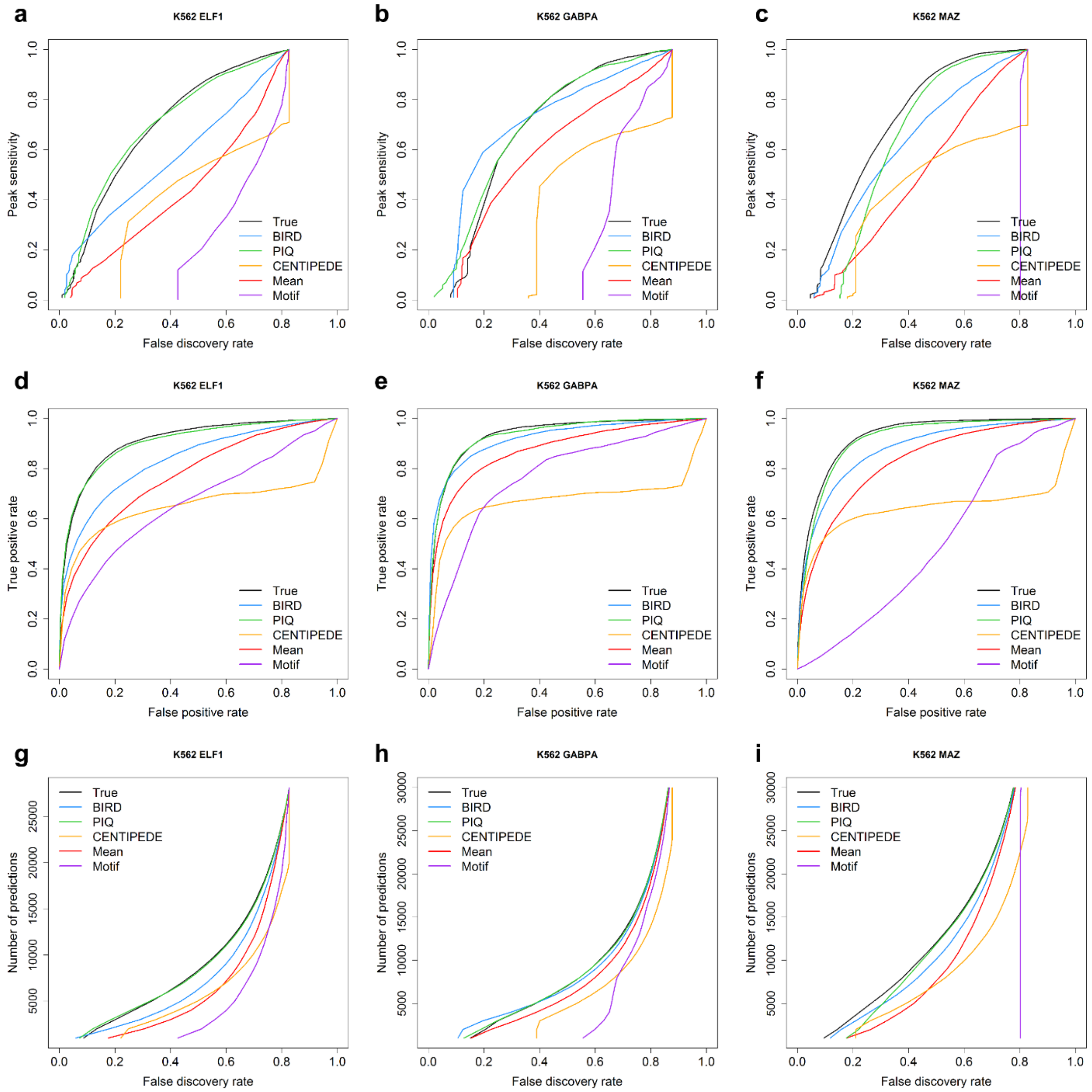

Supplementary Figure 27. Comparison of BIRD (based on gene expression from UW), PIQ, and CENTIPEDE for predicting binding sites of ELF1, GABPA, and MAZ in K562. Prediction using the true DNase-seq data ("True"), mean DH profile of training cell types ("Mean"), and the motif mapping score ("Motif") are also shown. (**a**)-(**c**) Sensitivity-FDR curves. (**d**)-(**f**) ROC curves. (**g**)-(**i**) The number of DHSs predicted to be TFBSs at different FDR levels.

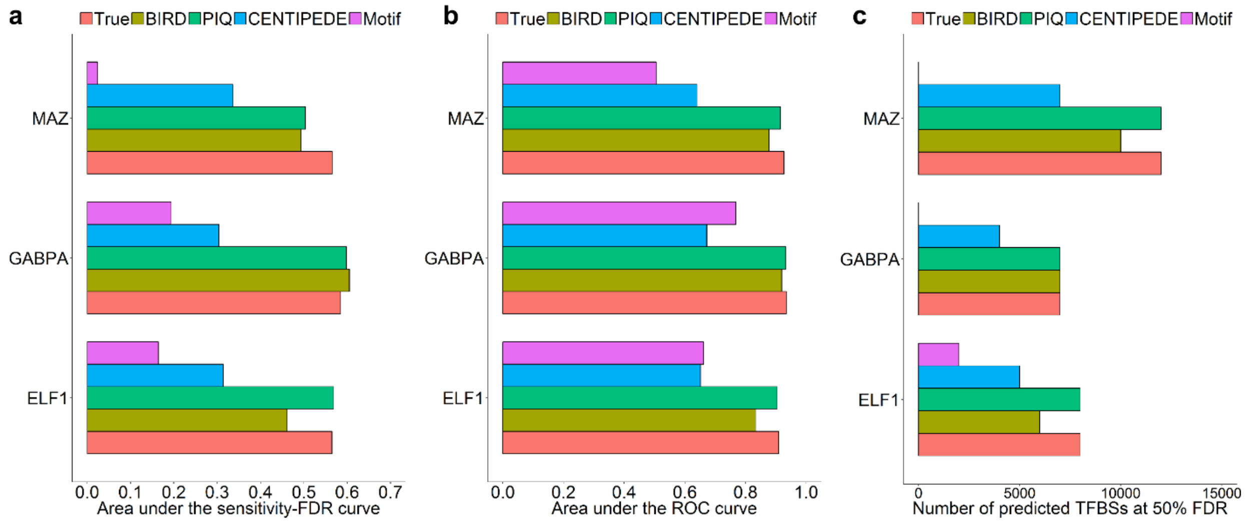

Supplementary Figure 28. Comparison of BIRD, PIQ, and CENTIPEDE in terms of (**a**) area under the sensitivity-FDR curve, (**b**) area under the ROC curve, and (**c**) number of predicted TFBSs at 50% FDR for predicting 3 TFs in K562. Results from prediction using the true DNase-seq data ("True") and the motif mapping score ("Motif") are also shown.

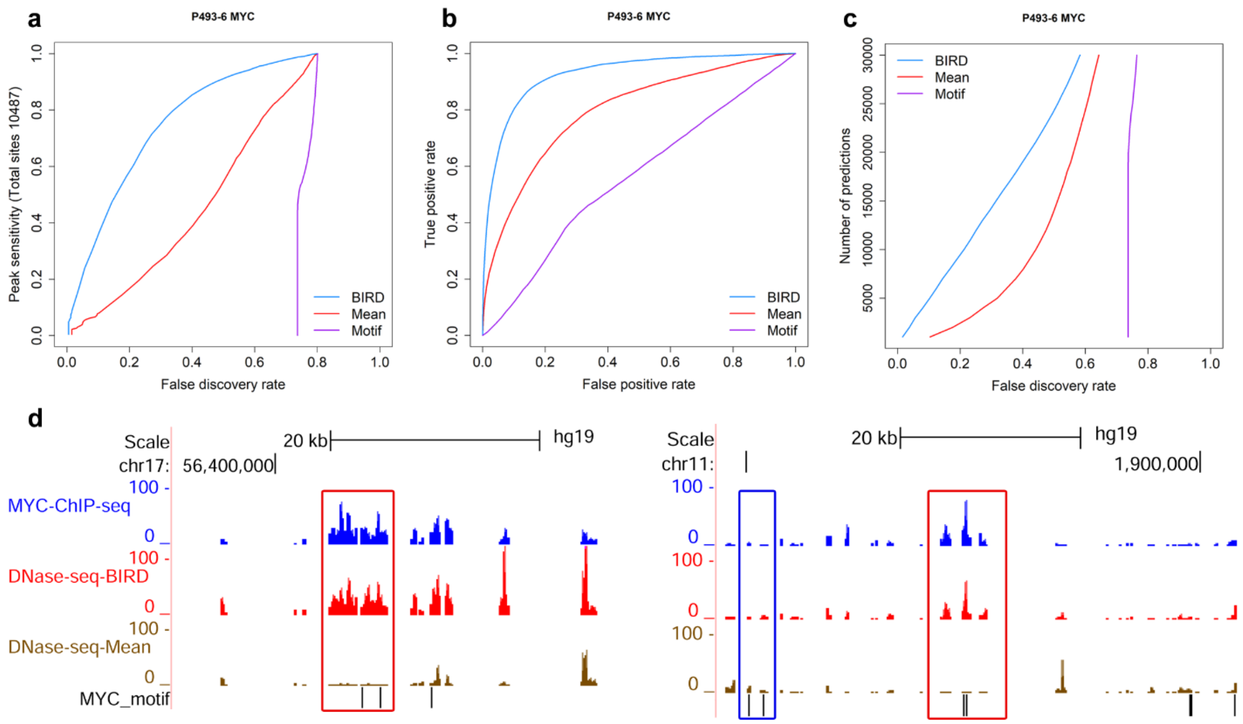

Supplementary Figure 29. Prediction of MYC binding sites in P493-6 cells using three different methods: BIRD, mean DH profile of training cell types ("Mean"), and the motif mapping score ("Motif"). (**a**) Sensitivity-FDR curve. (**b**) ROC curve. (**c**) The number of DHSs predicted to be TFBSs at different FDR levels. (**d**) Two examples showing the true MYC ChIP-seq signal (read count, blue track), the BIRD-predicted DH signal (red track), the mean DH profile of training cell types (brown track) and locations of MYC motif sites (bottom track) in two genomic regions. Highlighted in the red boxes are two MYC binding sites. Highlighted in the blue box are two motif sites not bound by MYC. BIRD accurately predicted the binding activities at MYC motif sites. The mean DH approach failed to do so. The motif only approach identified many motif sites not bound by MYC.

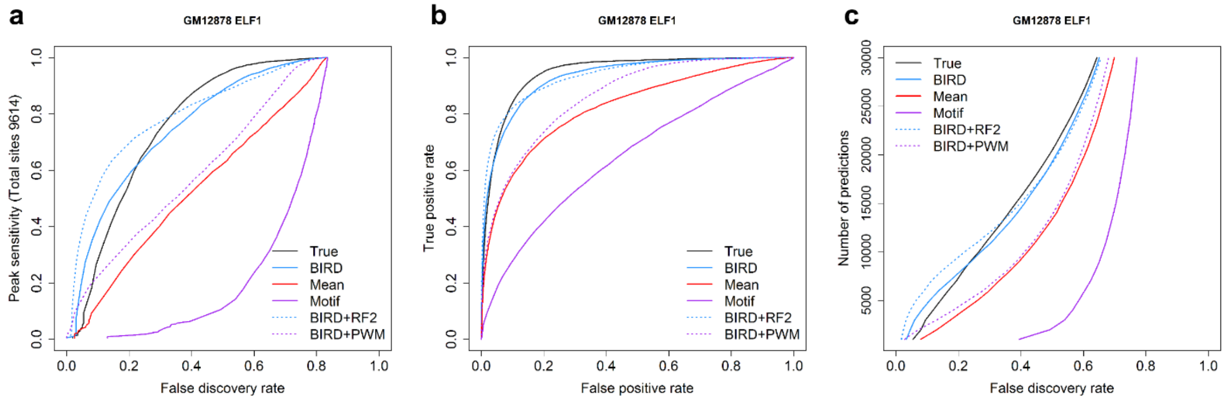

Supplementary Figure 30. Improved motif model increases the TFBS prediction performance. TFBS prediction results based on ranking motif-containing DHSs using true DH ("True"), BIRD-predicted DH ("BIRD") or mean DH of training data ("Mean"), based on motif PWM score only ("Motif"), based on the improved motif model (which accounts for intra-motif correlation) coupled with BIRD-predicted DH ("BIRD+RF2"), and based on PWM motif score coupled with BIRD-predicted DH ("BIRD+PWM") are compared. (**a**) Sensitivity-FDR curve, (**b**) ROC curve, and (**c**) the number of DHSs predicted to be TFBSs at different FDR levels for predicting ELF1 binding sites in GM12878.

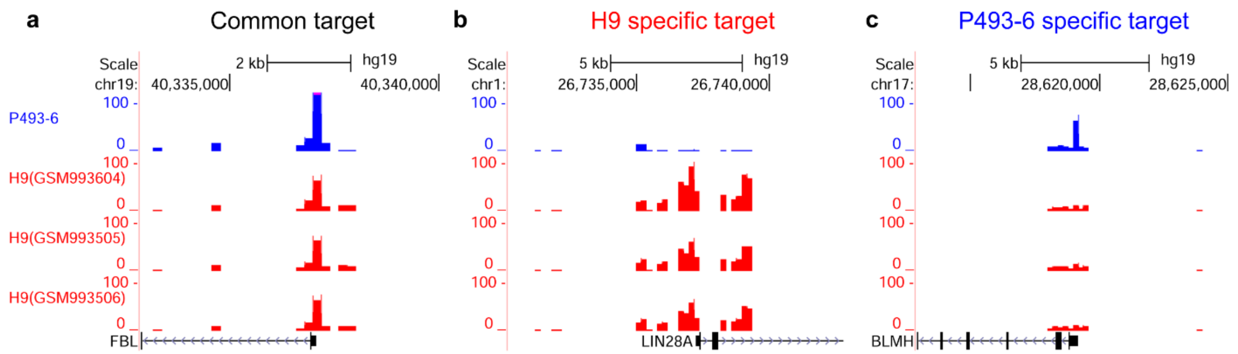

Supplementary Figure 31. Analysis of MYC targets *FBL*, *LIN28A* and *BLMH* using PDDB. Predicted DH in PDDB for different H9 samples showed similar patterns.

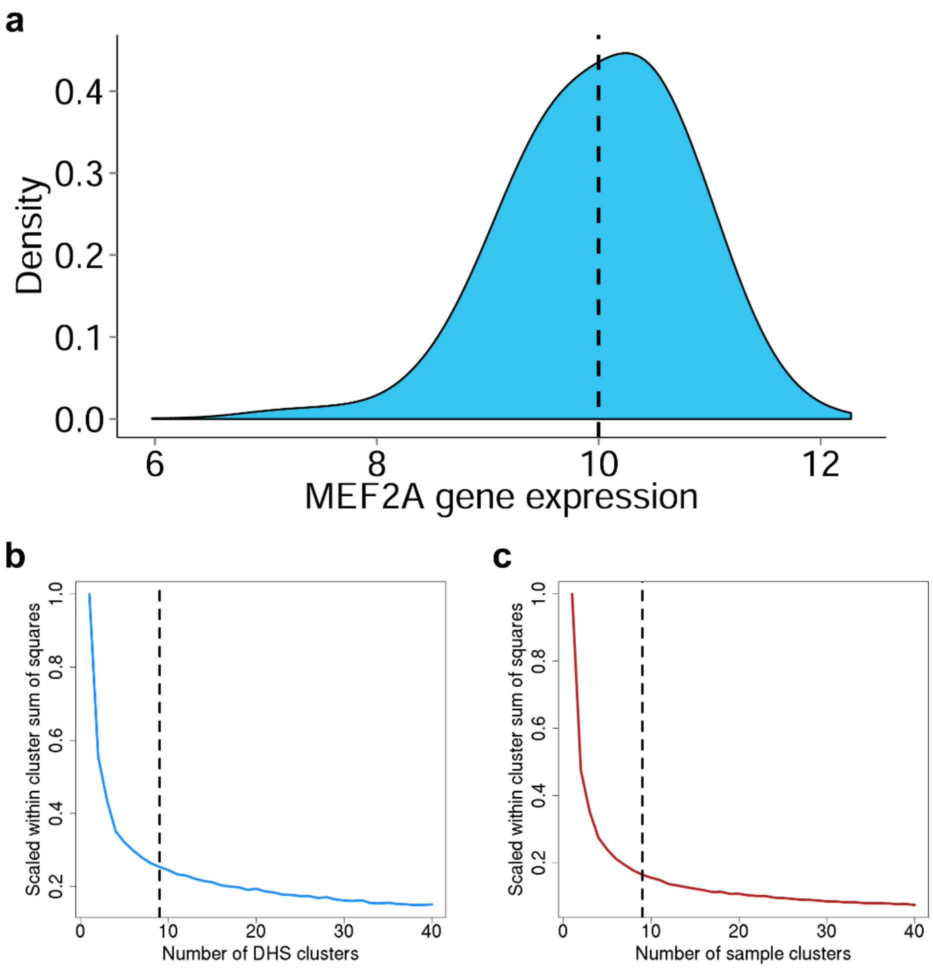

Supplementary Figure 32. Analyses of MEF2A binding sites using PDDB. (**a**) Distribution of *MEF2A* gene expression in all 2000 samples in PDDB. In the subsequent analysis, 1061 samples with *MEF2A* expression larger than the average *MEF2A* expression value (showed with dashed line) were obtained. (**b**) Selecting the cluster number (*K*) for clustering the 2011 DHSs. The curve shows the total within-cluster sum of squared error (*SSE*) for each number of *K* (=1, 2, 3, …, 40) scaled by *SSE* from *K*=1. The dashed line indicates the selected cluster number (*K*=9). (**c**) Selecting cluster number (*K*) for clustering the 1061 samples. Similarly, the curve shows the scaled *SSE* for different number of clusters. The dashed line indicates the selected cluster number (*K*=9).

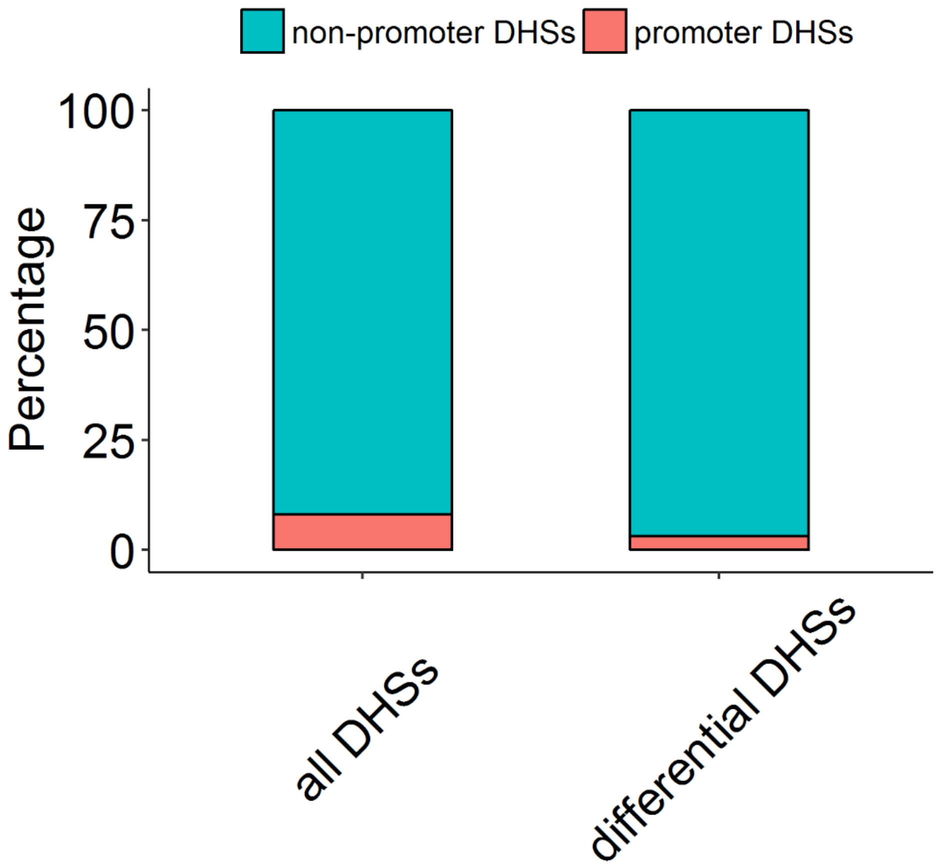

Supplementary Figure 33. Percentage of DHSs in promoter region (within ±1kb of transcription start sites) and non-promoter region (outside ±1kb of transcription start sites) calculated for all DHSs and differential DHSs in the analysis of neuron differentiation. In all DHSs, there are 88,887 promoter DHSs and 1,019,716 non-promoter DHSs. In differential DHSs, there are 2357 promoter DHSs and 74,138 non-promoter DHSs. Fisher's exact test shows that the proportions of non-promoter DHSs in differential DHSs is significantly larger than that in all DHSs (p-value < 10^{-15}).

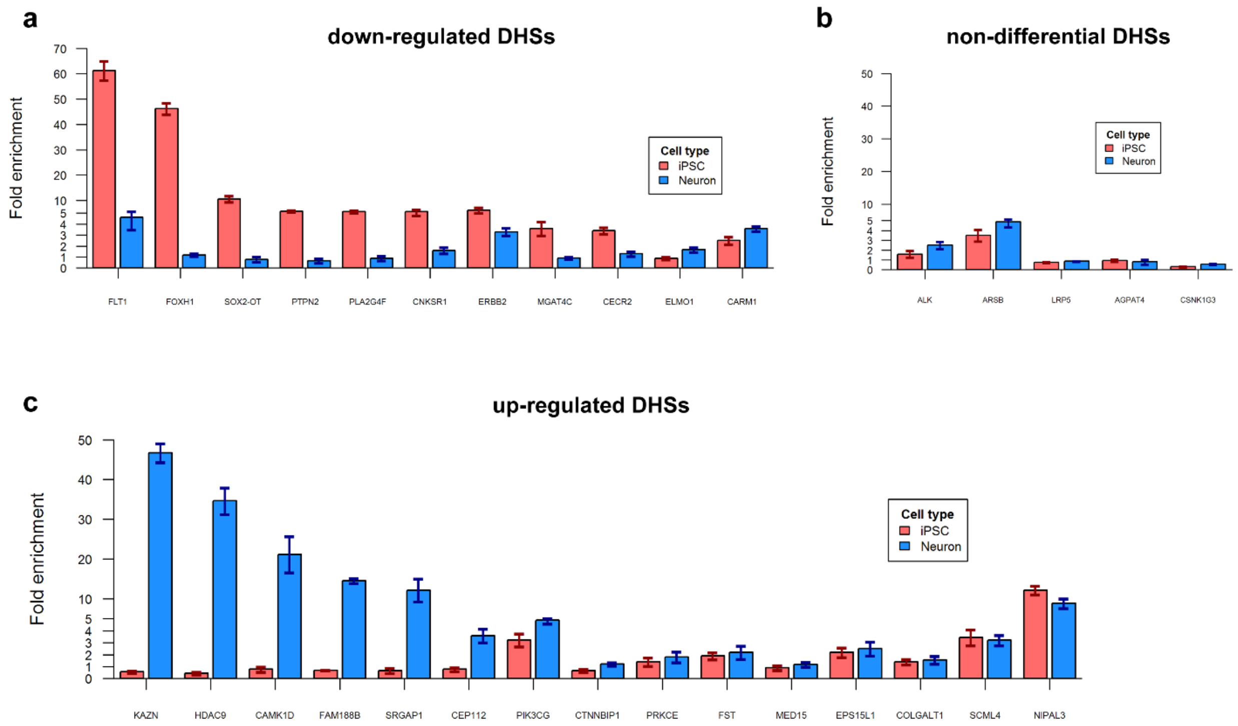

Supplementary Figure 34. H3K4me1 ChIP-qPCR result for (**a**) DHSs predicted to be down-regulated (i.e., with stronger signals in iPSCs), (**b**) non-differential DHSs (control), and (**c**) DHSs predicted to be up-regulated (i.e., with stronger signals in iPSC-derived neurons). For each locus, the mean qPCR fold enrichment relative to IgG control ($y = 2^{-(Ct_{avg}H3K4me1\ IP\ - Ct_{avg}IgG)}$) is shown for iPSCs and iPSC-derived neurons respectively. Data are presented as mean ± s.e.m (n = 3 technical replicates per condition per DHS).

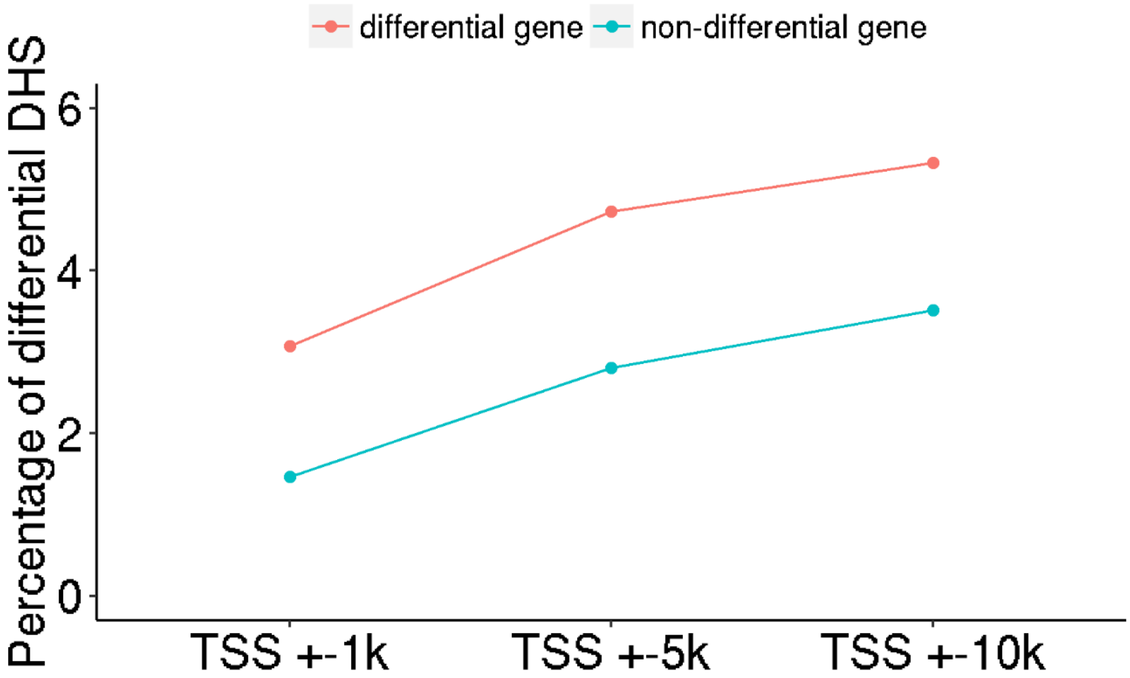

Supplementary Figure 35. Average percentage of DHSs located in the neighborhood (±10k, ±5k, or ±1k bp from TSS) of differential genes (red line) or non-differential genes (blue line) that are differential DHSs. The percentage of differential DHSs in differential gene regions is significantly larger than the percentage of differential DHSs in non-differential gene regions (One-sided Wilcoxon rank-sum test p-value $< 10^{-15}$ for comparing the two percentages within each neighborhood definition, i.e., ±10k, ±5k, or ±1k from TSS. For each neighborhood definition, the test involves comparing 4452 differential genes and 14,072 non-differential genes; see **Supplementary Methods**).

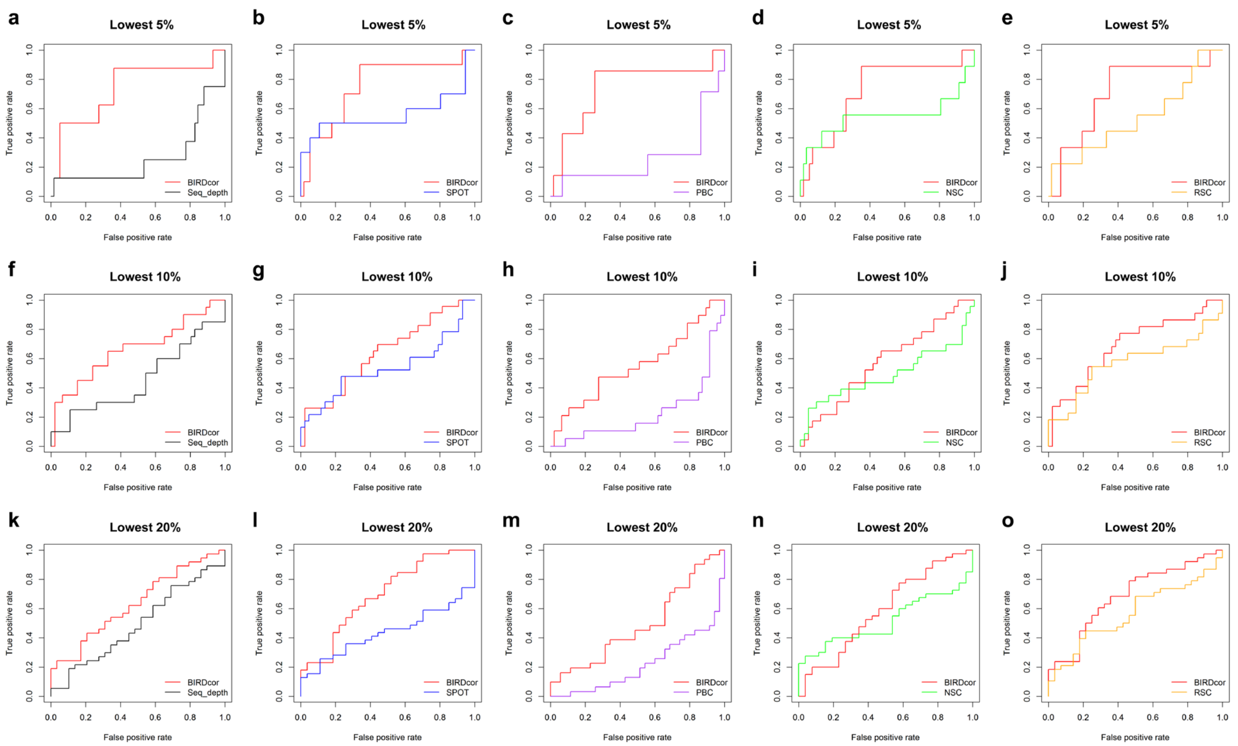

Supplementary Figure 36. ROC comparison of BIRD and each ENCODE QC metric for identifying low-quality samples. Low quality samples are defined by pooling the lowest (**a**)-(**e**) 5%, (**f**)-(**j**) 10%, and (**k**)-(**o**) 20% samples ranked by other quality metrics (**Supplementary Note 5**). Using these low quality samples as gold standard, the ROC curves of different QC metrics for detecting low quality samples are compared.

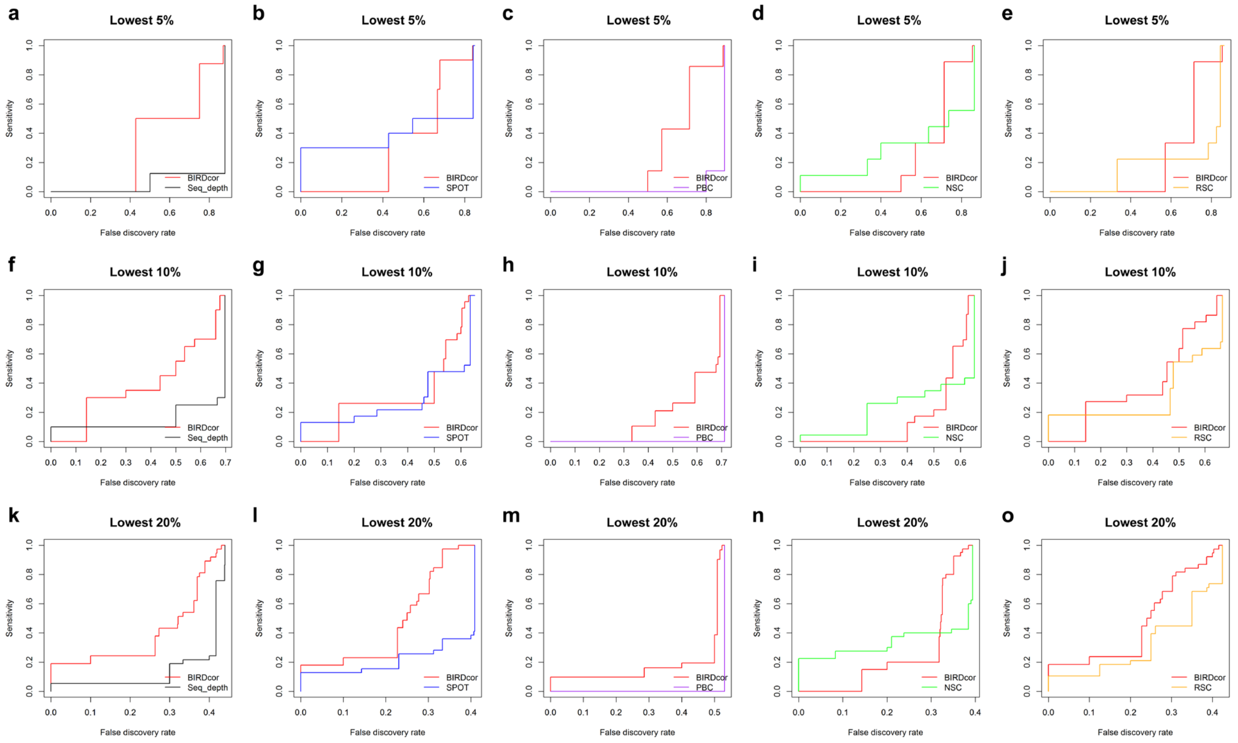

Supplementary Figure 37. Sensitivity-FDR comparison of BIRD and each ENCODE QC metric for identifying low-quality samples. Low quality samples are defined by pooling the lowest (**a**)-(**e**) 5%, (**f**)-(**j**) 10%, and (**k**)-(**o**) 20% samples ranked by other quality metrics (**Supplementary Note 5**). Using these low quality samples as gold standard, the sensitivity-FDR curves of different QC metrics for detecting low quality samples are compared.

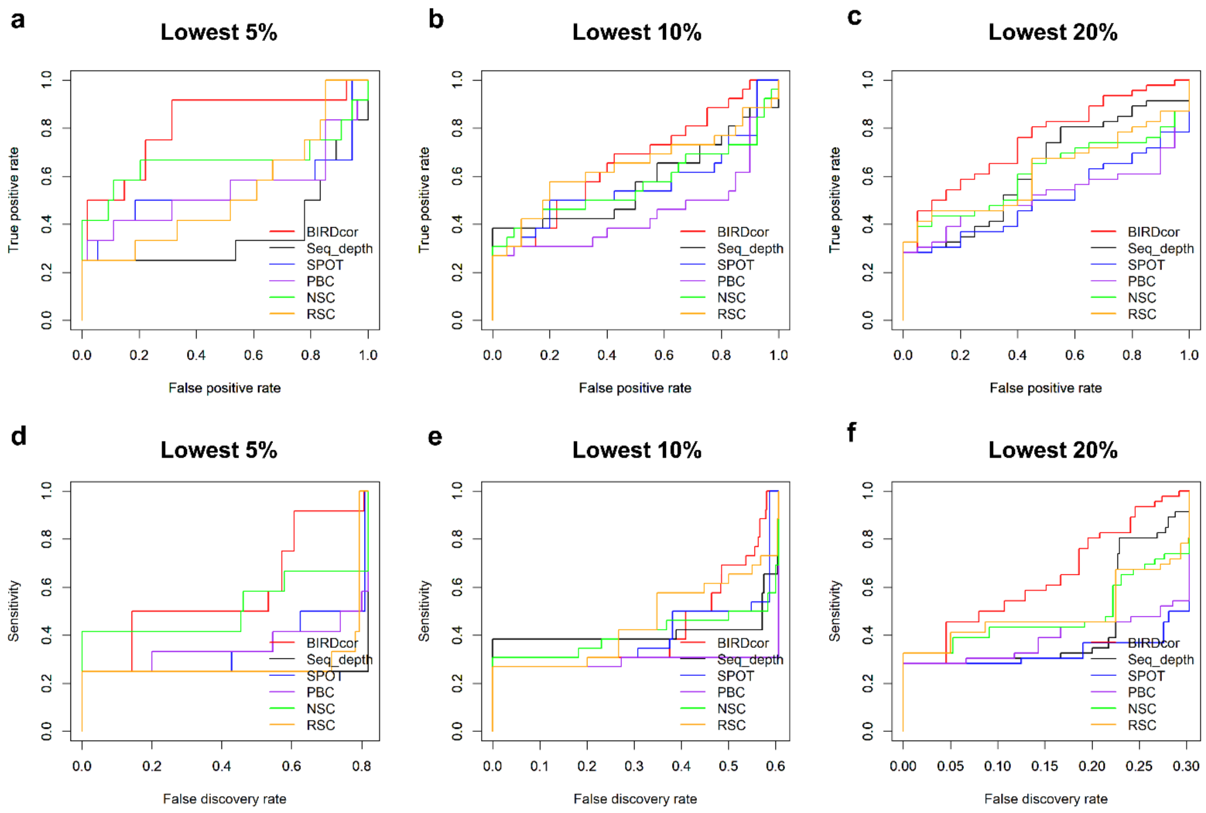

Supplementary Figure 38. Comparison of BIRD and ENCODE QC metrics to identify low-quality samples. Low quality samples are defined by pooling the lowest (**a**) 5%, (**b**) 10%, and (**c**) 20% samples ranked by each quality metric (**Supplementary Note 5**). Using these low quality samples as gold standard, the ROC curves of all QC metrics for detecting low quality samples are compared in (**a**)-(**c**). Similarly, the sensitivity-FDR curves of different QC metrics for detecting the low quality samples are compared in (**d**)-(**f**).

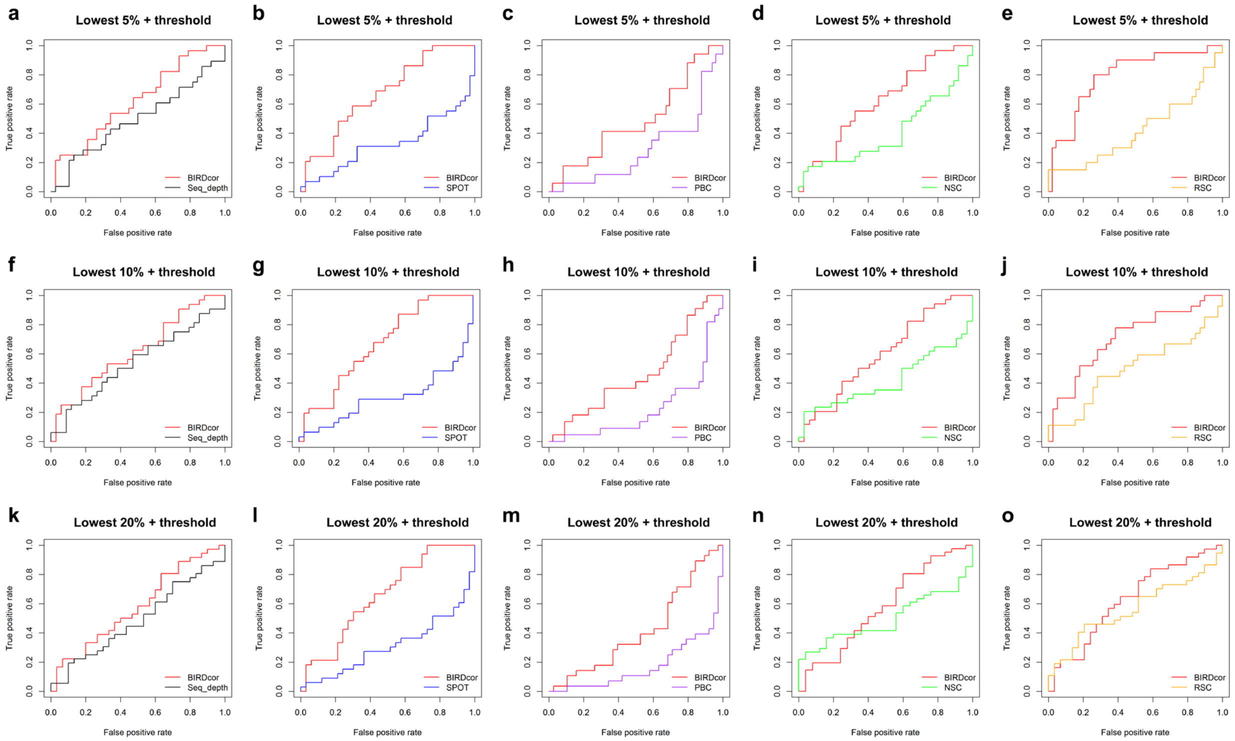

Supplementary Figure 39. ROC comparison of BIRD and each ENCODE QC metric for identifying low-quality samples. Low quality samples are defined by pooling the lowest (**a**)-(**e**) 5%, (**f**)-(**j**) 10%, and (**k**)-(**o**) 20% ranked samples from seq-depth and SPOT and samples below the ENCODE suggested threshold from PBC, NSC, and RSC after excluding the metric being tested (**Supplementary Note 5**). Using these low quality samples as gold standard, the ROC curves of different QC metrics for detecting low quality samples are compared.

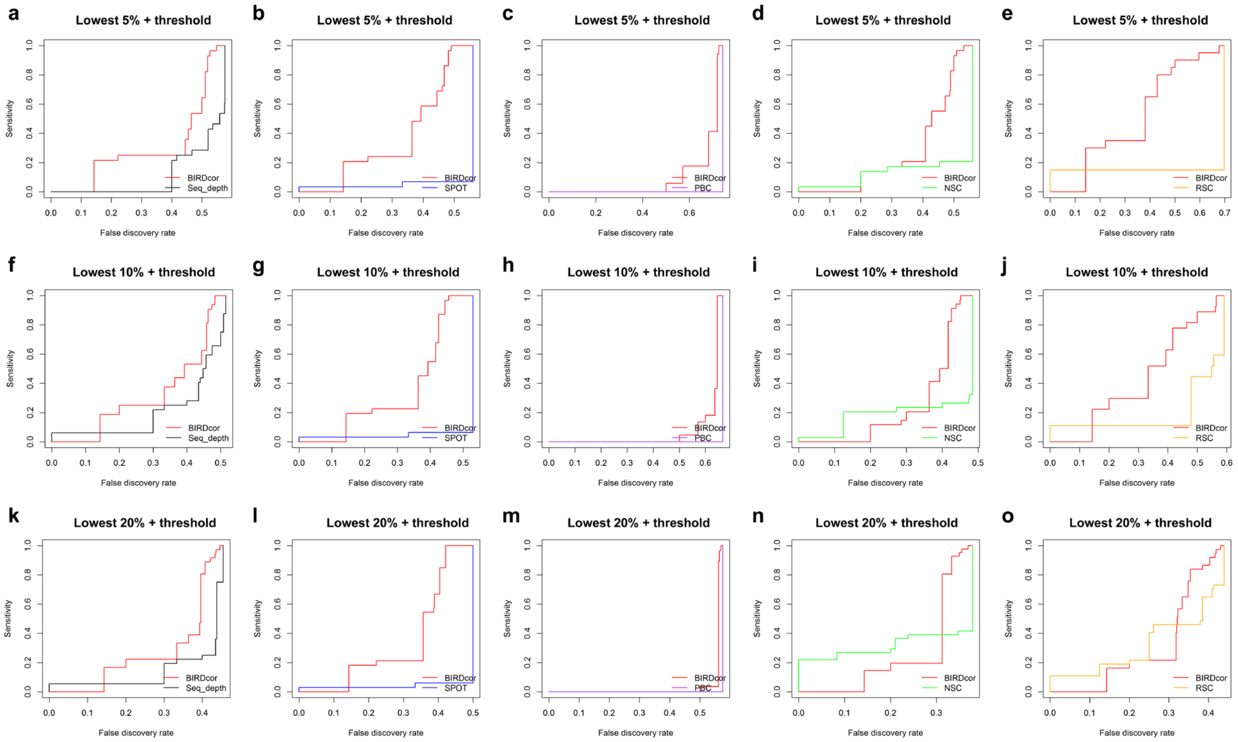

Supplementary Figure 40. Sensitivity-FDR comparison of BIRD and each ENCODE QC metric for identifying low-quality samples. Low quality samples are defined by pooling the lowest (**a**)-(**e**) 5%, (**f**)-(**j**) 10%, and (**k**)-(**o**) 20% ranked samples from seq-depth and SPOT and samples below the ENCODE suggested threshold from PBC, NSC, and RSC after excluding the metric being tested (**Supplementary Note 5**). Using these low quality samples as gold standard, the sensitivity-FDR curves of different QC metrics for detecting low quality samples are compared.

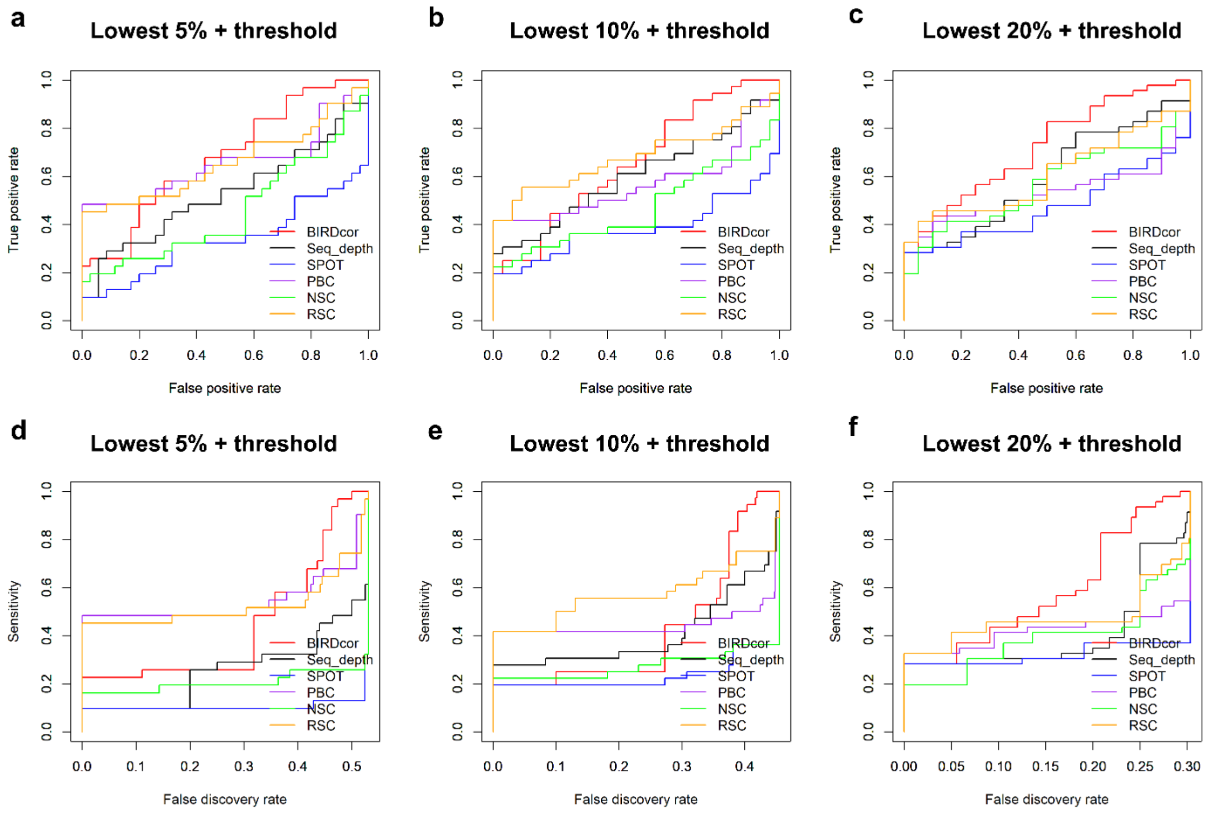

Supplementary Figure 41. Comparison of BIRD and ENCODE QC metrics to identify low-quality samples. Low quality samples are defined by pooling the lowest (**a**) 5%, (**b**) 10%, and (**c**) 20% ranked samples from seq-depth, SPOT and BIRDcor, and samples below the ENCODE suggested threshold from PBC, NSC, and RSC (**Supplementary Note 5**). Using these low quality samples as gold standard, the ROC curves of all QC metrics for detecting low quality samples are compared in (**a**)-(**c**). Similarly, the sensitivity-FDR curves of different QC metrics for detecting the low quality samples are compared in (**d**)-(**f**).

Group	Sample	BIRDcor rank	seq_depth rank	SPOT rank	PBC rank	NSC rank	RSC rank
Lowest 10% samples	Chorion_Rep1	1	59	60	23	13	40
	Osteobl_Rep2	2	52	63	5	52	1
	Hepatocytes_Rep2	3	47	16	11	4	57
	Chorion_Rep2	4	34	50	6	45	66
	Osteobl_Rep3	5	66	40	2	19	34
	Hepatocytes_Rep1	6	57	11	35	2	58
	Cll_Rep2	7	48	1	55	1	3
Random samples	Myometr_Rep2	19	31	25	24	10	33
	Hepg2_Rep2	20	3	52	54	62	23
	Hepg2_Rep3	24	11	29	59	54	37
	H1hesc_Rep2	36	56	65	10	39	18
	Gliobla_Rep2	44	49	30	41	37	24
	AosmcSerumfree_Rep2	60	18	6	49	15	20
	Gm19238_Rep2	64	24	49	22	56	30

Supplementary Figure 42. The 7 lowest ($\approx$ lowest 10%) ranked samples identified by BIRDcor and 7 randomly selected samples. Each column represents a QC metric. For each QC metric, samples with lowest quality were highlighted in color: dark red - lowest 10% (rank 1-7) samples; light red - lowest 20% (rank 8-13) samples.

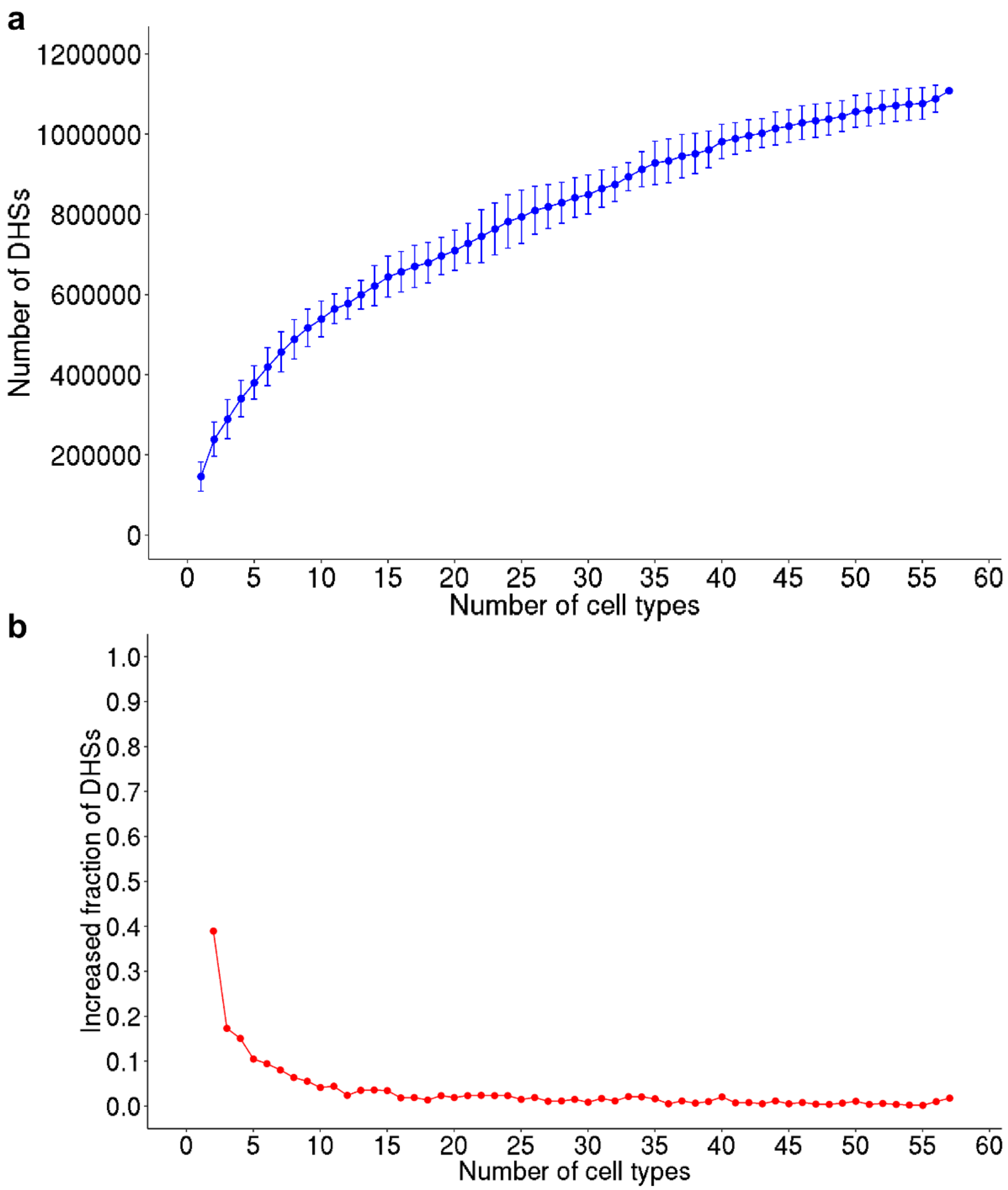

Supplementary Figure 43. The impact on the discovery of new DHSs by adding a new cell type to training data. (**a**) The number of DHSs (denoted by $Y(n)$) discovered from training data is shown as a function of the number of training cell types (denoted by n). Mean and standard deviation from 10 simulations are shown (see **Supplementary Note 6**). (**b**) Fraction of new DHSs contributed uniquely by adding a new cell type. This plot shows $[Y(n)-Y(n-1)]/Y(n)$ as a function of n. The mean from 10 simulations is shown.

Supplementary References

1. Ernst, J. & Kellis, M. Large-scale imputation of epigenomic datasets for systematic annotation of diverse human tissues. *Nat. Biotechnol.* **33**, 364-376 (2015).

2. Zhang, Y. *et al*. Model-based analysis of ChIP-Seq (MACS). *Genome Biol.* **9**, R137-2008-9-9-r137. Epub 2008 Sep 17 (2008).

3. Roadmap Epigenomics Consortium. Integrative analysis of 111 reference human epigenomes. *Nature* **518**, 317-330 (2015).

4. Thurman, R. E. *et al*. The accessible chromatin landscape of the human genome. *Nature* **489**, 75-82 (2012).

5. Wang, Z. *et al*. Combinatorial patterns of histone acetylations and methylations in the human genome. *Nat. Genet.* **40**, 897-903 (2008).

6. Matys, V. *et al*. TRANSFAC and its module TRANSCompel: transcriptional gene regulation in eukaryotes. *Nucleic Acids Res.* **34**, D108-10 (2006).

7. Mathelier, A. *et al*. JASPAR 2014: an extensively expanded and updated open-access database of transcription factor binding profiles. *Nucleic Acids Res.* **42**, D142-7 (2014).

8. Ji, H. *et al*. An integrated software system for analyzing ChIP-chip and ChIP-seq data. *Nat. Biotechnol.* **26**, 1293-1300 (2008).

9. Li, Q., Brown, J. B., Huang, H. & Bickel, P. J. Measuring reproducibility of high-throughput experiments. *The annals of applied statistics* **5**, 1752-1779 (2011).

10. Sherwood, R. I. *et al*. Discovery of directional and nondirectional pioneer transcription factors by modeling DNase profile magnitude and shape. *Nat. Biotechnol.* **32**, 171-178 (2014).

11. Pique-Regi, R. *et al*. Accurate inference of transcription factor binding from DNA sequence and chromatin accessibility data. *Genome Res.* **21**, 447-455 (2011).

12. Ji, H. *et al*. Cell-type independent MYC target genes reveal a primordial signature involved in biomass accumulation. *PloS one* **6**, e26057 (2011).

13. Sabò, A. *et al*. Selective transcriptional regulation by Myc in cellular growth control and lymphomagenesis. *Nature* **511**, 488-492 (2014).

14. Langmead, B., Trapnell, C., Pop, M. & Salzberg, S. L. Ultrafast and memory-efficient alignment of short DNA sequences to the human genome. *Genome Biol.* **10**, R25 (2009).

15. Huang, D. W., Sherman, B. T. & Lempicki, R. A. Bioinformatics enrichment tools: paths toward the comprehensive functional analysis of large gene lists. *Nucleic Acids Res.* **37**, 1-13 (2009).

16. Huang, D. W., Sherman, B. T. & Lempicki, R. A. Systematic and integrative analysis of large gene lists using DAVID bioinformatics resources. *Nature protocols* **4**, 44-57 (2008).

17. Potthoff, M. J. & Olson, E. N. MEF2: a central regulator of diverse developmental programs. *Development* **134**, 4131-4140 (2007).

18. Flavell, S. W. *et al*. Genome-wide analysis of MEF2 transcriptional program reveals synaptic target genes and neuronal activity-dependent polyadenylation site selection. *Neuron* **60**, 1022-1038 (2008).

19. Hystad, M. E. *et al*. Characterization of early stages of human B cell development by gene expression profiling. *J. Immunol.* **179**, 3662-3671 (2007).

20. Edmondson, D. G., Lyons, G. E., Martin, J. F. & Olson, E. N. Mef2 gene expression marks the cardiac and skeletal muscle lineages during mouse embryogenesis. *Development* **120**, 1251-1263 (1994).

21. Sagal, J. *et al*. Proneural transcription factor Atoh1 drives highly efficient differentiation of human pluripotent stem cells into dopaminergic neurons. *Stem Cells Transl. Med.* **3**, 888-898 (2014).

22. Kriks, S. *et al*. Dopamine neurons derived from human ES cells efficiently engraft in animal models of Parkinson's disease. *Nature* **480**, 547-551 (2011).

23. Rozen, S. & Skaletsky, H. Primer3 on the WWW for general users and for biologist programmers. *Methods in molecular biology* **132**, 365-386 (2000).

24. Ritchie, M. E. *et al*. limma powers differential expression analyses for RNA-sequencing and microarray studies. *Nucleic Acids Res.* **43**, e47-e47 (2015).

25. ENCODE Project Consortium. An integrated encyclopedia of DNA elements in the human genome. *Nature* **489**, 57-74 (2012).

26. Tibshirani, R. Regression shrinkage and selection via the lasso. *Journal of the Royal Statistical Society.Series B (Methodological)* **58**, 267-288 (1996).

27. Friedman, J., Hastie, T. & Tibshirani, R. Regularization Paths for Generalized Linear Models via Coordinate Descent. *J. Stat. Softw* **33**, 1-22 (2010).

28. Hocking, R. R. A Biometrics invited paper. The analysis and selection of variables in linear regression. *Biometrics* **32**, 1-49 (1976).

29. Altman, N. S. An introduction to kernel and nearest-neighbor nonparametric regression. *The American Statistician* **46**, 175-185 (1992).

30. Beygelzimer, A. *et al*. FNN: fast nearest neighbor search algorithms and applications. *R package version 1.1* (2013).

31. Breiman, L. Random forests. *Mach. Learning* **45**, 5-32 (2001).

32. Liaw, A. & Wiener, M. Classification and regression by randomForest. *R news* **2**, 18-22 (2002).

33. Yuan, M. & Lin, Y. Model selection and estimation in regression with grouped variables. *Journal of the Royal Statistical Society: Series B (Statistical Methodology)* **68**, 49-67 (2006).

34. Breheny, P. grpreg: Regularization paths for regression models with grouped covariates. *R package version 3.0-2* (2016).

35. Breheny, P. & Huang, J. Penalized methods for bi-level variable selection. *Stat. Interface* **2**, 369-380 (2009).

36. Zhang, C. Nearly unbiased variable selection under minimax concave penalty. *The Annals of statistics* **38**, 894-942 (2010).

37. Tibshirani, R., Saunders, M., Rosset, S., Zhu, J. & Knight, K. Sparsity and smoothness via the fused lasso. *Journal of the Royal Statistical Society: Series B (Statistical Methodology)* **67**, 91-108 (2005).

38. Goeman, J., Meijer, R. & Chaturvedi, N. penalized: L1 (lasso and fused lasso) and L2 (ridge) penalized estimation in GLMs and in the Cox model. *R package version 0.9-45* (2014).

39. Jolliffe, I. T. A note on the use of principal components in regression. *Applied Statistics* **31**, 300-303 (1982).

40. Andersson, R. *et al*. An atlas of active enhancers across human cell types and tissues. *Nature* **507**, 455-461 (2014).

41. Kodzius, R. *et al*. CAGE: cap analysis of gene expression. *Nature methods* **3**, 211-222 (2006).

42. Benjamini, Y. & Hochberg, Y. Controlling the false discovery rate: a practical and powerful approach to multiple testing. *Journal of the royal statistical society.Series B (Methodological)* **57**, 289-300 (1995).

43. Alexa, A. & Rahnenfuhrer, J. topGO: enrichment analysis for gene ontology. *R package version 2.8* (2010).

44. Siepel, A. *et al*. Evolutionarily conserved elements in vertebrate, insect, worm, and yeast genomes. *Genome Res.* **15**, 1034-1050 (2005).

45. Mukherjee, S. *et al*. Rapid analysis of the DNA-binding specificities of transcription factors with DNA microarrays. *Nat. Genet.* **36**, 1331-1339 (2004).

46. Zhou, Q. & Liu, J. S. Modeling within-motif dependence for transcription factor binding site predictions. *Bioinformatics* **20**, 909-916 (2004).

47. Ben-Gal, I. *et al*. Identification of transcription factor binding sites with variable-order Bayesian networks. *Bioinformatics* **21**, 2657-2666 (2005).

48. Kulakovskiy, I. *et al*. From binding motifs in ChIP-Seq data to improved models of transcription factor binding sites. *Journal of bioinformatics and computational biology* **11**, 1340004 (2013).
