# Supplementary material for: Genome-wide prediction of DNase I hypersensitivity using gene expression
Source: Nat Commun. 2017 Oct 19;8:1038. doi: 10.1038/s41467-017-01188-x (PMC5715040; doi:10.1038/s41467-017-01188-x)
Supplement: Supplementary file 3 — Description of Additional Supplementary Files [file 41467_2017_1188_MOESM3_ESM.pdf]

Description of Supplementary Files

File name: Supplementary Data 1
Description: Description of cell types used for training and testing. The 57 cell types were partitioned into a training set (40 cell types) and a testing set (17 cell types). We tried five different random partitions listed in the table. All these partitions produced similar results. Results from partition 1 were presented in the main manuscript.

File name: Supplementary Data 2
Description: Analysis of predictors selected by BIRD for a sample DHS pathway. This file contains multiple sheets: 1. [Active cell types] contains the active status of cell types in the DHS pathway. 2. [Predictor genes] contains the predictor selected by BIRD for the DHS pathway. 3. [Enriched motifs] contains the enriched motifs for the genomic loci within the DHS pathway. 4. [GO analysis] contains the GO terms enriched in the predictor genes of the DHS pathway.

File name: Supplementary Data 3
Description: Motif accession numbers for transcription factor binding motifs used in this study. The motifs were obtained from TRANSFAC and JASPAR.

File name: Supplementary Data 4
Description: List of SOX2 binding sites obtained from H9 human embryonic stem cell ChIP-seq data and the list of overlapped DHSs. This file contains 2 sheets: 1. [SOX2 peaks (FDR<0.01)] contains the SOX2 binding sites from two sample analysis using CisGenome. 2. [Overlapped DHS] contains the genomic loci (DHSs) that overlapped with the SOX2 binding sites.

File name: Supplementary Data 5
Description: List of DHSs for the analysis of MEF2A activity using PDDB.

File name: Supplementary Data 6
Description: Enriched functional annotations reported by DAVID for each DHS cluster in the MEF2A analysis. The 2011 DHSs were grouped into 9 clusters. This file contains 9 sheets. Each sheet contains functional annotations for one DHS cluster. For example, [DHS-cluster-1] contains the functional annotations in DHS cluster 1.

File name: Supplementary Data 7
Description: Annotation of sample types contained in each sample cluster in the MEF2A analysis. The 1061 samples were grouped into 9 clusters. This file contains 9 sheets. Each sheet contains the annotation of samples for a sample cluster. For example, [sample-cluster-1] contains the annotation of samples in sample cluster 1.

File name: Supplementary Data 8
Description: H3K4me1 ChIP-qPCR analysis for selected DHSs. This file contains 3 sheets: 1. [differential DHSs] contains the results for the 26 selected DHSs at different predicted fold change levels. 2. [control] contains the results for the 5 selected non-differential DHSs. 3. [primer sequence] contains the primers used in the experiment.

File name: Supplementary Data 9
Description: Enriched functional annotations reported by DAVID for differential genes associated with differential DHSs in neuron differentiation.

File name: Supplementary Data 10

Description: Enriched TF motifs from down-regulated and up-regulated DHSs after neuron differentiation. This file contains 2 sheets: 1. [down-regulated DHSs] contains the enriched TF motifs from the down-regulated DHSs (i.e., DHSs showed stronger signal in iPSCs). 2. [up-regulated DHSs] contains the enriched TF motifs from the up-regulated DHSs (i.e., DHSs showed stronger signal in neurons).

File name: Supplementary Data 11
Description: Comparison of QC measurements for 66 DNase-seq samples.
